# Epitranscriptomic analysis reveals clinical and molecular signatures in glioblastoma

**DOI:** 10.1186/s40478-025-01966-5

**Published:** 2025-04-11

**Authors:** Glaucia Maria de Mendonça Fernandes, Wesley Wang, Saman Seyed Ahmadian, Daniel Jones, Jing Peng, Pierre Giglio, Monica Venere, José Javier Otero

**Affiliations:** 1 Departament of Cellular and Molecular Medicine, Florida International University Herbert Wertheim College of Medicine, Miami, FL USA; 2 Department of Pathology, The Ohio State University Wexner Medical Center, Columbus, OH USA; 3 Center for Biostatistics, The Ohio State University College of Medicine, Columbus, OH USA; 4 Department of Neuro-oncology, The Ohio State University Wexner Medical Center, Columbus, OH USA; 5 Department of Radiation Oncology, James Cancer Hospital and Comprehensive Cancer Center, The Ohio State University College of Medicine, Columbus, OH USA; 6 Departament of Neuropathology and Clinical Informatics, Baptist Health South Florida, Miami, FL USA

**Keywords:** Glioblastoma, Progression disease, Pseudo-progression, Novel enhancement, Epitranscriptome

## Abstract

This study characterizes the glioblastoma (GB) epitranscriptomic landscape in patient who evolve to progressive disease (PD) or pseudo-progressive disease (psPD). Novel differences in N6-Methyladenosine (m6A) RNA methylation patterns between these groups are identified in the first biopsy. Retrospective data of patients that were eventually deemed to have progressive disease or pseudoprogressive disease was captured from the electronic health record, and RNA from the first resection specimen was utilized to evaluate N6-methyladenosine (m6A) biomarkers from FFPE samples. Molecular analysis of m6A methylation modified RNA employed ACA-based *RNase MazF* digestion. After Quantitative Normalization with ComBat to mitigate batch effects, we identifed differentially methylated transcripts and gene expression analyses, co-expression networks analyses with WGCNA, and subsequently performed gene set GO and KEGG enrichment analyses. Enrichments for metabolic biological processes and pathways were identified in our differential methylated transcripts and select module eigengene networks highlighted key co-expressed genes intricately tied to distinct phenotypes/traits in patients that would ultimately be deemed PD or psPD. Our study identified key genes and pathways modified by m6A RNA methylation associated with cell metabolism alterations, highlighting the importance of understanding m6A mechanisms leading to the oncometabolite accumulation governing PD versus psPD patients. Furthermore, these data indicate that epitranscriptomal differences between PD versus psPD are detected early in the disease course.

**Supplementary Information:**

The online version contains supplementary material available at 10.1186/s40478-025-01966-5.

## Introduction

Glioblastoma, IDH-wild type (GB) is the predominant adult malignant primary central nervous system (CNS) cancer accounting for approximately 14.2% of all primary brain tumors and 50.9% of all malignant tumors [33, 34]. GBs exhibit complex heterogeneity, posing challenges for accurate clinical and pathological evaluations [33]. Despite advancements in imaging techniques, molecular profiling, and therapeutic strategies, patient outcomes have not significantly improved over the past decade [34]. Standard treatment encompasses surgery, radiotherapy, and chemotherapy (chemoRT) [43], and querying clinicaltrials.gov for the term “Glioblastoma” yields over 1900 active research studies at the date of writing this manuscript. Thus, a major goal for diagnostic neuropathology includes identification of novel biomarkers that can match patients to optimal treatment protocols.

Following gross total resection, patients undergo non-invasive surveillance by serial magnetic resonance imaging (MRI) to detect any emerging contrast-enhancing lesions, which may indicate potential tumor recurrence. Notably, genuine cancer progression (progressive disease, or PD) is determined when the tumor persists, grows, or spreads within the brain despite undergoing therapeutic interventions. On the other hand, pseudo-progressive disease (psPD) refers to a transient worsening detected by imaging and/or clinical exam, that despite resembling tumor recurrence on imaging, actually represents a reactive process to the treatment intervention. Distinguishing between psPD and PD accurately is important to avoid unnecessary and potentially harmful changes to treatment plans. While PD may require treatment revision or palliation, psPD patients can be monitored or managed symptomatically with steroids without changing their oncological care [58].

Therefore, the growing understanding of the molecular intricacies underlying GB has underscored the pressing need to identify distinctive biomarkers capable of discerning clinically heterogeneous subgroups, such as predicting which patients experience PD versus psPD. Recent advances in epigenetic analysis, specifically exploring the N6-methyladenosine (m6A) RNA modification within the epitranscriptome, have emerged as promising areas of biomarker development in several other cancer paradigms. The m6A RNA modification is prevalent, reversible, and regulates gene expression and various biological processes within cells. This chemical modification entails adding a methyl group to the adenosine nucleotide base within RNA molecules. m6A can be found in mRNA as well as other RNA classes, including long non-coding RNAs (lncRNAs), microRNAs (miRNAs) and circular RNA (circRNAs). This modification can influence RNA metabolism by modifying RNA splicing, RNA stability, RNA translation, and RNA localization. Thus, m6A modification impacts various cellular functions and pathways [13]. Recent studies have highlighted the significant impact of the m6A modification on GB survival by regulating tumor mutation, microenvironment infiltration, and resistance to mTOR inhibitors [4, 23, 63]. Furthermore, m6A modification correlates with increased tumor aggressiveness, exhibiting variability among long non-coding RNAs. This variability impacts patient prognosis and could facilitate the identification of subtypes for prognostic predictions in glioma patients [19, 55]. In the present study, we comprehensively characterize the m6A epitranscriptomic landscape in GB patients, comparing PD and psPD groups in Cohort A and Cohort B to identify potential m6A epitranscriptome modification-derived biomarkers for future clinical neuropathology practice.

## Methods

### Patient data collection

GB patient data were retrospectively evaluated from January 2012 to March 2020 at The Ohio State University (IRB study number 2020C0062). Inclusion criteria comprised GB management by Ohio State, MGMT (O6-methylguanine-DNA methyltransferase) methylation testing, with exclusion criteria involving IDH mutation, low-grade glioma history, and poorly documented disease courses, recorded in REDcap [50]. All samples were diagnosed as glioblastoma, IDH-wild type, World Health Organization (WHO) grade 4 as per WHO 2021 diagnostic guidelines.

Following specific criteria, patients were categorized into psPD and PD groups: psPD was identified by new or progressive enhancement within 6 months of ChemoRT, confirmed treatment reaction, and no prior progression. PD classification relied on clinic-pathologically confirmed cancer recurrence with no previous progression history. The inclusion time points for psPD aligned with the recommended 6-month surveillance intervals [50]. Further demographic and clinicopathological details are presented in Table 1.

**Table 1:** Clinicopathological profile of glioblastoma patients in cohort A and cohort B cohorts

	Cohort A *N* = 56 (61%)		Cohort B *n* = 36 (39%)		*p*-value	Effect Size
**Diagnostic** ^a^						0.43 ^2^
PD	41	(37.2)	18	(50)	0.04*	
psPD	15	(37.7)	18	(50)		
**Gender** ^a^						
Male	35	(62.5)	24	(66.6)	0.85	0.04 ^1^
Female	21	(37.5)	12	(33.3)		
**Age (years)** ^b^	62	(13.7)	67	(10.0)	0.10	0.17 ^1^
**BMI** ^b^	27.9	(9.2)	29.1	(8.2)	0.35	0.11 ^1^
**Side** ^c^						
Left	28	(50.0)	21	(58.4)	0.57	0.11 ^1^
Right	28	(50.0)	15	(41.6)		
**Lesion Size (cm)** ^c^	4.1	(1.8)	4.0	(2.0)	0.71	0.07 ^1^
**Lobe Location** ^a^						0.67 ^2^
Frontal	**21**	**(37.5)**	8	(22.2)		
Occipital	**0**	**(0)**	5	(13.8)	0.02*	
Parietal	16	(28.5)	8	(22.2)		
Temporal	19	(33.9)	15	(41.6)		
**Midline Shift** ^a^						0.22 ^2^
Yes	23	(41.1)	10	(27.7)	0.28	
No	33	(58.9)	26	(72.3)		
**Ki-67 Positive Percent** ^b^	30.0	(20.0)	27.5	(22.2)	0.24	0.12 ^1^
**MGMT Status** ^a^						0.13 ^1^
Hypermethylated/ Methylated	23	(41.1)	18	(50.0)	0.53	
Not Hypermethylated/ Unmethylated	33	(58.9)	18	(50.0)		
**EGFR Amplification** ^a^						0.44 ^2^
No	29	(70.7)	19	(59.4)	0.11	
Yes	12	(29.3)	13	(40.6)		
**P53 Mutation Status (> 10% of Cells)** ^a^						0.18 ^1^
Negative	11	(22.0)	7	(20.6)	0.68	
Positive	39	(78.0)	27	(79.4)		
**WBC (Pre-surgery)** ^b^	11.2	(6.5)	9.1	(4.6)	0.16	0.14 ^1^
**Hemoglobin (Pre-surgery)** ^c^	14.1	(2.3)	14.5	(1.8)	0.55	0.06 ^1^
**Platelets (Pre-surgery)** ^c^	247.8	(71.9)	233.4	(61.1)	0.30	0.21 ^2^
**Neutrophils (Segs + Bands) (Pre-surgery)** ^c^	9.2	(4.2)	7.8	(4.4)	0.13	0.32 ^2^
**Lymphocytes (Pre-surgery)** ^c^	1.2	(0.7)	1.4	(0.6)	0.21	0.30 ^2^
**Number of Gy administered** ^a^						0.39 ^2^
40	5	(8.9)	4	(11.1)	1.00	
60	51	(91.1)	32	(88.9)		
**Number of Gy Fractions** ^a^						0.00 ^1^
15	5	(8.9)	4	(11.1)	1.00	
30	51	(91.1)	32	(88.9)		
**Cycles Completed** ^b^	5	(5.0)	5.5	(3.0)	0.69	0.04 ^1^
**Adjuvant Temodar Dose** ^b^	280	(190.0)	355	(106.2)	0.02*	0.24 ^2^

^a^ n (%), ^b^ median (IQR = interquartile range), ^c^ mean (SD = standard deviation)

^1^ small (≤ 0.2), ^2^ moderate (= 0.5), ^3^ large (≥ 0.8)

BMI = Body Mass Index, WBC = White Blood Cell

*p-value significant (*p* < 0.05) tested using Fisher exact test for Lobe location variable, chi-square for categorical variables, and t-test or Wilcoxon for continuous variables

**Fig. 1:**
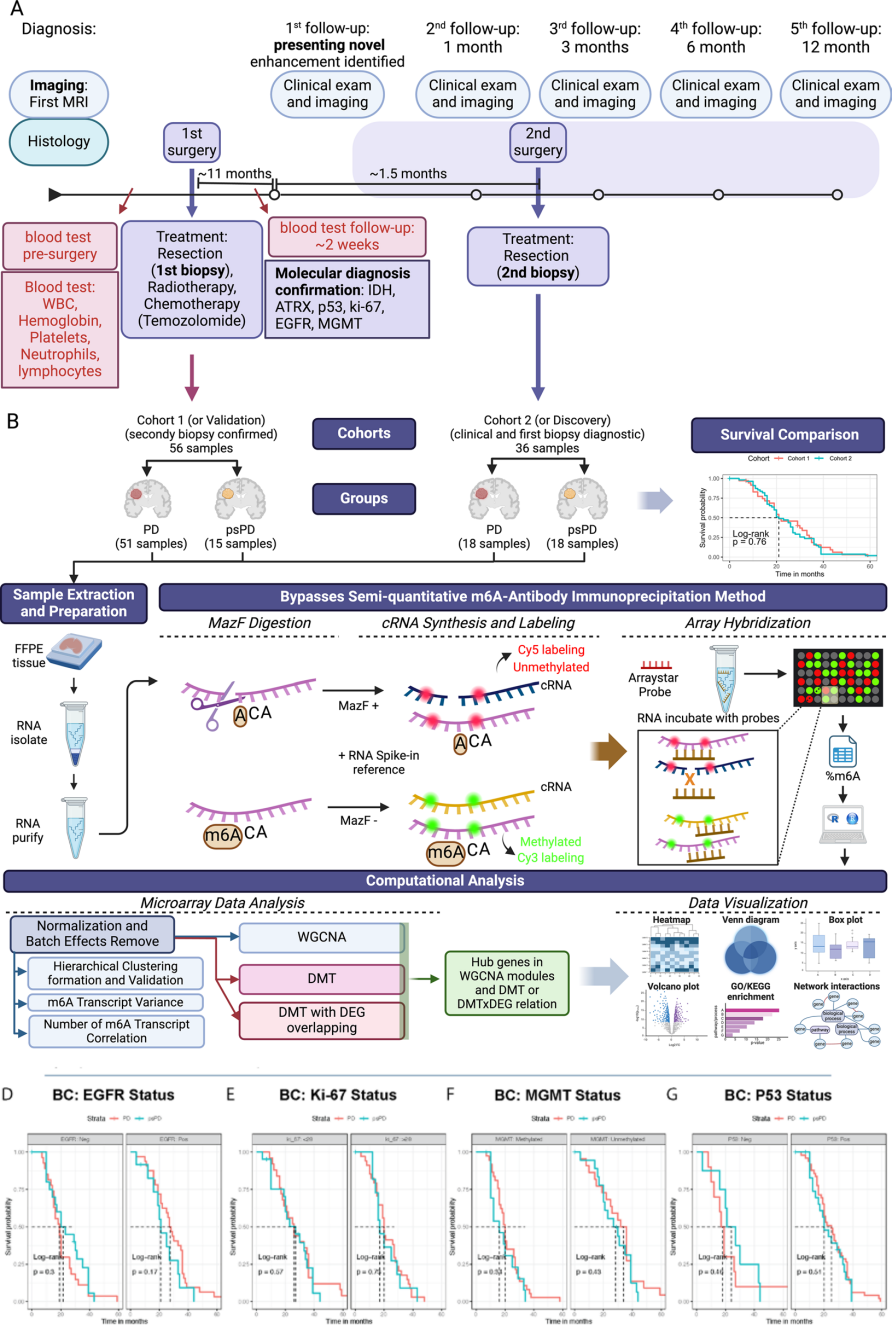
Methodology workflow and Kaplan-Meier survival analysis comparing glioblastoma patients. (**A**) The study design illustrates the clinical and imaging diagnostic process and patient monitoring, highlighting the specific points of biopsy collection and treatment initiation. This shows the time points at which our two study cohorts were sampled. Additionally, it delineates the blood testing and molecular marker assessment points utilized in this study, along with the clinical data. (**B**) The methodology workflow to access the m6A RNA methylation patters of this study., begins with retrospective Glioblastoma patient data collection, categorizing patients into progression disease (PD) and pseudo progression disease (psPD). FFPE tissue specimens was used for molecular analysis. RNA extraction, quality assessment, and m6A RNA methylation site identification was performed using the m6A Single Nucleotide Array Service using the MazF enzyme to digest the unmethylated (ACA) sites on genes and labeling with Cy5 fluorophore (red) in the microarray analysis, while the methylated sites was undigested (m6ACA) by MazF enzyme are labeling with Cy3 fluorophore (green). The data of Cohort B and Cohort A cohorts pass underwent normalization, batch adjustment, and analysis of m6A variance. Further analysis involved differential gene expression, methylation, and exploration of site-specific m6A methylation relationships. Co-expression networks were constructed using WGCNA. R software and packages was employed to analysis and visualization throughout the entire process and statistical analysis. (**C**) showcases the overall comparison between Cohort 1 and Cohort 2 survival in both Groups. The impact of (**D**) EGFR, (**E**) KI-67, (**F**) MGMT, and (**G**) P53 markers on survival outcomes. Kaplan-Meier survival curves and log-rank test for statistical analysis for assessing significance (*p* < 0.05)

Tissue specimens for molecular analysis were selected based on clinical history as PD or psPD, determined by an interdisciplinary tumor board. All samples were collected from the original second surgery and stored as archival formalin-fixed paraffin-embedded (FFPE) tissue. Specifically, 36 samples [PD: 18; psPD: 18] constituted the Cohort B (all of which had undergone a second confirmatory resection), and 56 samples [PD: 41; psPD: 15] comprised the Cohort A (none of which had undergone a second confirmatory resection). These samples fell within study-defined time points and provided adequate tissue for molecular study (Fig. 1A).

The second biopsy patients, based on clinical indication and patient tolerance, may introduce biases that affect the generalizability of the results. Additionally, repeat biopsies present significant challenges, including limitations in sample size available for analysis and variability in histopathological samples. Distinguishing between true progressive disease, residual radiated tumor, and inflammatory pseudoprogression is particularly challenging given the ongoing debate regarding appropriate histopathological criteria. We acknowledge that these limitations may influence the findings and interpretation of the data and emphasize the need for additional prospective studies to validate our observations and refine the diagnostic criteria used.

### m6A RNA analysis

RNA extraction utilized the FFPE tissue RNA Purification Kit [Cat. 25300, 25400], following the manufacturer’s guidelines. Quality assessment involved RNA concentration, purity, and integrity using ThermoFisher NanoDrop, ThermoFisher Qubit, and Agilent Tapestation [50]. Subsequently, methylation sites on RNA transcripts were identified utilizing the m6A Single Nucleotide Array Service following manufacturer instructions (Arraystar Inc.). This technique presented in Fig. 1B employs RNase MazF to cleave single-stranded RNA immediately adjacent to the unmethylated (ACA) sequence, while preserving the methylated (m6ACA) sequence. The labeled RNA fragments, representing cleaved ACA (Cy5, red) or uncleaved m6ACA (Cy3, green), along with undigested input RNA, were analyzed using a dual-color system on specialized arrays. This targeted technique employs specialized arrays to precisely identify, map and quantity specific m6A RNA modification sites at the resolution of single nucleotides, providing high-resolution, versatile detection across contexts and well-documented robustness in scientific literature. The transcripts unmethylated are used to analyze the gene expression.

### Normalization and batch adjustment

Removing batch effects and using surrogate variables have been shown to reduce dependence, stabilize error rate estimates and improve reproducibility in small samples. For the methylated and unmethylated data, five batch adjustment methods were compared in the data set [*n* = 92 samples] using RStudio: (i) Quantile Normalization (QN), (ii) pamr.batchadjust function on Pam: prediction analysis for microarrays (PamR) package [45, 46], performing gene-wise one-way ANOVA adjustments on expression values, subtracting the mean expression of a gene across all samples in a specific batch from each corresponding samples expression for that gene; (iii) combination of QN and PamR adjustment function; (iv) Combatting batch effects when combining batches of microarray data (ComBat) function in Surrogate Variable Analysis (SVA) Package [21], employing a parametric empirical Bayesian approach, effectively adjusts high-dimensional data matrices for known batches, passing the full model matrix created without any known batch variables (separate argument to the function) [14]; and (v) combination of QN and ComBat adjustment function [24].

### Purity and significance of m6A% identified clusters

To evaluate the diversity and complexity within the GB patient cluster, we quantify the percentage of methylated RNA at m6A in patient cohorts. Hierarchical clustering was employed to unveil patterns in the methylation data. The patient cohort underwent careful selection based on hierarchical cluster formation and heatmap analysis criteria.

To facilitate a comprehensive assessment of complexity differences between the GB patient diagnosis and clinical features clusters. The significance of identified clusters in m6A methylated data was confirmed using Fisher’s exact test implemented through R software.

### m6A variance

All transcripts were classified according to variability between the samples by each m6A RNA methylated transcript, revealing a subset comprising the lowest 30% or highest 30% using Z-score measures on RStudio, provided valuable insights into the pattern and stability of methylation markers within specific biological contexts, facilitating the identification of significant methylation patterns.

**Fig. 2:**
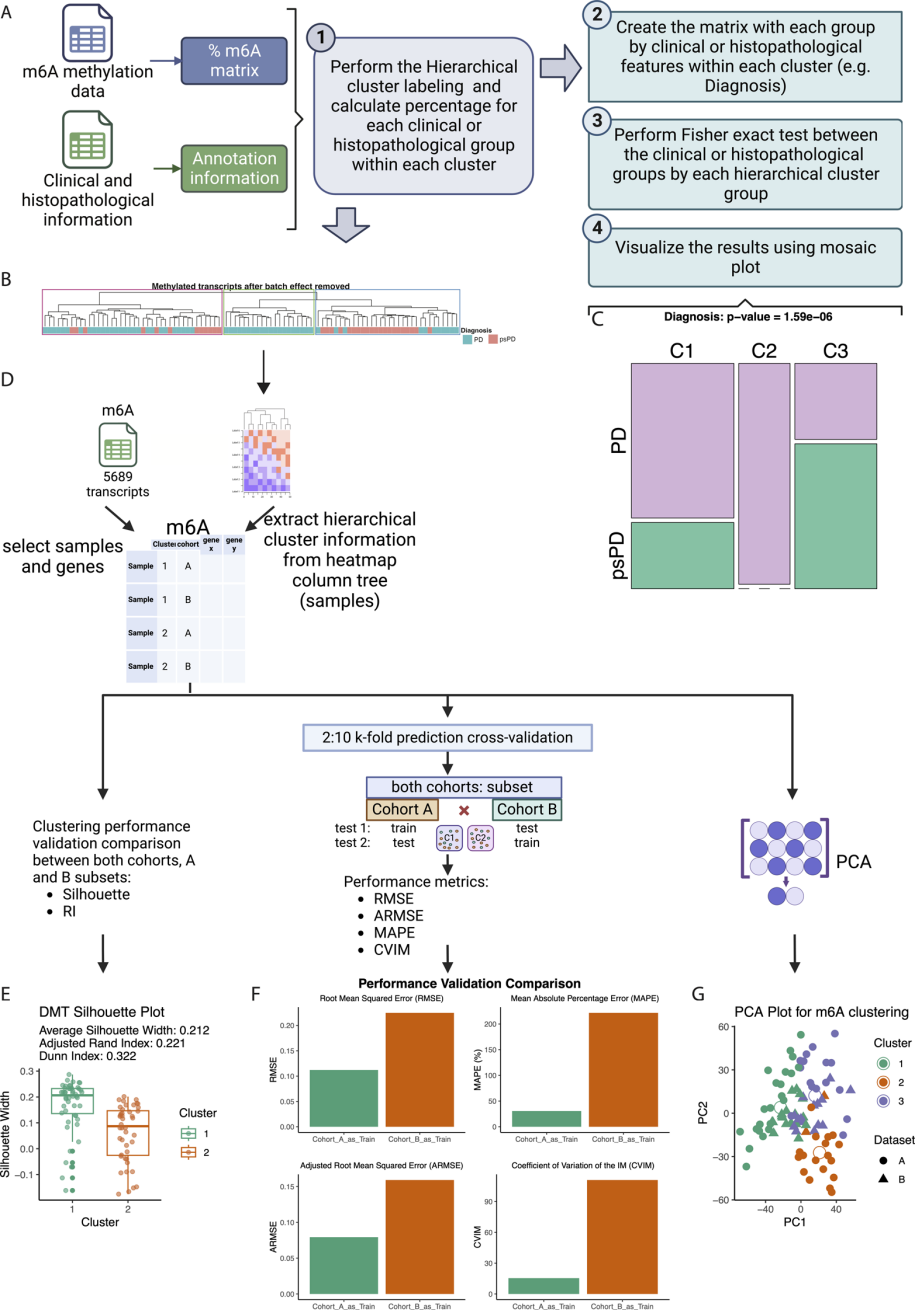
Validating the purity of hierarchical cluster labeling (**A**) Workflow outlines the sequential steps involved in the comparison of label purity in clinical and histopathological groups. (**B**) Heatmap showcasing the comprehensive array of Diagnosis variable among the PD/psPD patients with glioblastoma. (**C**) Mosaic plot compares cluster formation across the Diagnosis variable within the studied PD/psPD patient cohort by Fisher’s Exact Test. (**D**) Workflow of the performance and validation of the HC. (**E**) Boxplot showing silhouette and ARI metric. (**F**) Barplot showing RMSE, ARMSE, MAPE and CVIM metrics comparing Cohort A as a training and Cohort B as test and the inverse. (**G**) Distribution plot of the three clusters, showing the two most relevant principal component

### Hierarchical clustering correlation across m6A%, DMT and DMT with DEG overlapping

To evaluate the diversity and complexity within the GB patient cluster, we quantify the percentage of methylated RNA at m6A in patient cohorts. Hierarchical clustering was employed to unveil patterns in the differential methylation data and for DMT with DEG overlapping. The patient cohort underwent careful selection based on hierarchical cluster formation and heatmap DMT and DMT with DEG overlapping analysis criteria.

To facilitate a comprehensive assessment of complexity differences between the GB patient diagnosis and the significance of identified clusters in m6A% across DMT and DMT with DEG overlapping, we performed a k fold cross-validation to predict the clustering performance using Cohort A and Cohort B as train and test alternatively. The RMSE, ARMSE, MAPE and CVIM metrics was performed to evaluate these predicted models.

We implemented a PCA analysis using *caret* package and plotted using the alluvial plot to compare the clustering formation. Complementary, we also calculate clustering performance and quality metrics including Adjusted Rand Index (ARI) using *fpc* package. ARI range − 1 (totally randomly) to 1(perfect agreement) assesses the similarity between clustering results adjusted for chance [15].

### Bias mitigation for pathway enrichment analysis

The inclusion of probes on the detection chip provided by the manufacturer contains a selection bias as they (1) were chosen to have annotated m6A modification in other studies, and (2) had been shown to contain ACA sites amenable to our biochemical workflows. This selection bias made it difficult to perform GO enrichment analysis as the transcripts included in the assay created a pre-existing false positive enrichment [18, 48]. To address this concern, we performed a relative enrichment analysis, where we took our gene sets identified in our computational analyses and performed enrichment relative to the enrichment present on the chip using Chi-square analysis. Chi-square test was used to compare the methylated and all probes matrix (probe analyzed by Arraystar Inc) data between the groups. P-value < 0.05 indicating a significant difference in m6A RNA methylation distribution. The results are complementing the GO enrichment data with a in the Additional file 1: Table S2, this GO modified data offers a critical role of methylation status in shaping the outcomes of our study. This approach is designed to confirm the dependability and precision of the data on differential methylation, offering insights into potential biomarkers and the biological significance of the results.

### Complex relationships exist in the context of site-specific m6A RNA methylation

The methodology involved a thorough investigation of correlations between the number and each site-specific m6A methylation across genetic loci, contributing to methylation accumulation in genes. The Pearson correlation coefficient was utilized in R software to assess the relationship between expression and m6A transcript sites, designating transcripts with coefficients near − 1 and 1 as positively and negatively correlated, respectively, and those close to 0 as having no significant linear relationship. Additionally, a linear regression analysis was conducted in R software to explore the association between each m6A methylation site and the number of genes influencing expression.

### Co-Expression networks construction and computational validation

Weighted gene correlation network analysis (WGCNA) was performed with all 5690 methylated transcripts using the R WGCNA package to construct a gene co-expression network comparing the PD and psPD groups [60]. In the modules with significant enrichment (adjusted *p* > 0.05) by Fisher’s exact test of biologic processes and molecular functions based on GO and KEGG we performed the t-test to compare groups in each cohort and perform the volcano plot analysis to show the differential methylated transcripts in each module. Modules with non-significant enrichments pathways were removed in data visualization [26]. To validate the significant modules, we performed the Boruta algorithm analysis using *Boruta* function in R. Boruta uses the random forest regression to determine the methylated transcripts in each significant module could predict gene expression (7,140 transcripts in the total unmethylated data). To access this, we created a matrix to analyze the results of the Boruta analysis constructing a factor with three levels Confirmed, Rejected or Tentative and using UMAP dimensionality reduction to visualize the differences. We select only unique genes present in the Confirmed results to perform the heatmap and GO analysis.

### Differential gene expression and differential methylation transcriptions analysis and visualization

We performed differential expression analysis and differential methylation analysis. We utilize the acronym DEG (differentially expressed genes) to refer to differential gene expression analysis when comparing PD to psPD groups using as a threshold p-value < 0.05 and log 2-fold change (log2FC) ≥ 2. We utilize the acronym DMT to refer to the fold change in percentage of m6A RNA methylated transcripts (DMT) between PD and psPD groups. Subsequently, we conducted a co-joint analysis that integrated the DEG and DMT. These transcripts were further categorized based on their methylation status among PD versus psPD patients, considering the adjusted p-value < 0.05 and log 2-fold change (log2FC) ≥ 2.5 as a threshold a: hypermethylated transcripts log2FC > 2.5, indicating higher m6A methylation levels in PD Glioblastoma, and hypomethylated transcripts log2FC >-2.5, indicating lower methylation levels. Within these categories, we identified specific gene subsets, such as hypermethylated genes that were up or downregulated and hypomethylated genes that were also up or downregulated. Visualization employed EnhancedVolcano to show differential expressions in DMT, Venn diagrams to illustrate the overlap between DEG and DMT, and heatmap tools. We also conducted pathway analyses, using Gene Ontology (GO, 2021) and Kyoto Encyclopedia of Genes and Genomes (KEGG, 2021) pathways via bar plots in enrichR-Knowledgebase (enrichR-KG, 2023) online tool [2, 5, 7] and the subnetworks was constructed using igraph R package to contextualize the biological significance.

### Statistical testing

Demographic variables were summarized by descriptive statistics. Continuous variables were described using mean (standard deviation) and compared with two sample t test if they are approximately normally distributed, otherwise median (IQR) and Mann-Whitney U test were used to compare two groups. To determine the effect size, we employed Cohen’s *d* measure and *r* value is calculated as Z statistic, respectively. Categorical variables were summarized using frequency (percentages) and compared using chi-square test or Fisher’s exact test if we observed small counts in some categories the index *w* was calculated to determine the effect size. Log-rank test was used to compare the survival probabilities between Cohort A and Cohort B. Survival curves were generated using the Kaplan-Meier estimator. Boxplot comparisons were statistically validated through one-way ANOVA and pairwise comparisons using Welch’s t-test in R. Post-hoc contrasts was performed using the emmeans package in R with Bonferroni p-value correction (50). All the model and test assumptions will be checked by residual plots or histograms of variables.

### List of R packages utilized

The primary used R packages were dplyr; factoextra; caret; cluster; fpc; tidyverse; randomForest; PamR, SVA, survival; survminer; ggplot2; ggpubr; WGCNA; Boruta; EnhancedVolcano; sjstats; emmeans; org.Hs.eg.db; pheatmap; EnrichR; igraph.

## Results

### Distinguishing GB PD from PsPD cannot be achieved by standard histopathology assays of the initial resection tissue

Overall survival and on progression free survival in GB show significant variance amongst patients. Predicting GB progression from the initial resection has eluded the neuropathology community, and it is unknown if the original resection material contains any information capable of shedding light on events that occur in a patient’s disease evolution several months later. To address this knowledge gap, we generated a REDCAP database of GB patients treated at The Ohio State University and who were evaluated by neuro-oncology when a surveillance imaging showed contrast enhancing lesion, representing either PD or psPD. This database included basic clinical data as well as information from the patient’s first contrast enhancing lesion which was deemed to be either PD or psPD [49, 50]. The workflow of our experimental design is illustrated in Fig. 1. Briefly, from our database we identified a group of 36 patients composed of 18 PD and 18 psPD deemed Cohort B that had undergone a gross total resection and were biopsied for confirmation of the diagnosis at the point of the first contrast enhancing lesion. We also identified an additional 56 patients (41 PD and 15 psPD), deemed Cohort A, who were followed but who did not undergo a second biopsy. RNA was extracted from the formalin fixed, paraffin embedded tissue sections from the initial resection and submitted to m6A analysis by the MAZF digestion method [61]. Data analysis was performed in RStudio.

The demographic data, split by Cohort, are detailed in Table 1. In both cohorts, the clinical diagnosis of PD or psPD were delineated by the treating neuro-oncologist after incorporating the clinical-pathological data as delineated in Wang, et al. [50]. Note that patients included in this study required manifestation of a contrast enhancing lesion, which represents only in a subset of GB-related deaths due to tumor progression [3]. We note that these cohorts showed some differences, including a higher number of PD diagnoses in Cohort A. Tumors located in the Occipital region were absent in the Cohort A, whereas the Frontal region showed a more diverse distribution across groups. Regarding clinical biomarkers, we did not identify statistically significant differences between Cohort A vs. Cohort B and PD vs. psPD in MGMT promoter status (determined by sequencing), EGFR amplification (determined by FISH), ki-67 labelling index, and p53 mutation (determined by immunohistochemistry). These data indicate that standard molecular profiles remained consistent between these two GB patient cohorts. Additionally, to initiate our exploration of group differences, the diagnostic breakdown of GB patients into PD versus psPD groups around both Cohort A and Cohort B data sets is detailed in Additional file 1: Table S1. In summary, these data indicate that standard molecular profiles do not predict which patients would manifest a PD or a psPD.

When evaluated from the time of the initial gross total resection, the overall survival between PD and psPD patient groups did not differ significantly between Cohort A and Cohort B (*p* = 0.76) (Fig. 1C). We note that in all cohorts, the mean overall survival from the initial gross total resection was slightly over 20 months, which we attribute to having included only patients who were managed long enough for neuro-oncology to deem as PD or psPD at the point of their CEL manifestation. Furthermore, we evaluated potential associations between critical genetic markers commonly assayed in routine neuropathology practice, such as EGFR amplification, P53 mutation by immunohistochemistry, Ki-67 labeling index, and MGMT promoter methylation, with patient survival. Consistent with survival outcomes, no significant differences were observed in these genetic biomarkers between the cohorts (Fig. 1D-G). These observations highlight that genetic markers in clinical routine practice do not distinguish prognosis and overall survival between PD and psPD GB groups.

### Combination of quantile normalization and combat approach provides optimal normalization for m6A RNA methylation data to mitigate batch effects while preserving biological variability in GB

In order to delineate the extent to which epitranscriptional changes are associated with patient outcomes, we assed m6 RNA methylation in transcripts with ACA sequences amenable to detection by the MAZF procedure, hereon referred to as m6A, from archival FFPE tissue blocks that underwent RNA extractions as delineated above. As epitranscriptomics analysis represents an emerging analytical process within neuropathology, debate exists as to the optimal normalization methods. In other informatics paradigms, an extensive body of literature has delineated the challenges of high-throughput “omics” analyses due to technical variations such as noise and batch effects, all of which complicate cross-comparisons between groups [9, 14, 24]. To address these issues, several computational tools have been developed to achieve data normalization to enable group comparisons [10, 40, 52, 62]. Many of these normalization protocols do not adjust the data for batch effects, thus necessitating the implementation of additional procedures [9, 14, 32, 57, 62]. In our study, we observed distinct patterns suggesting the existence of potential group-specific differences in RNA methylation levels across the samples that could be caused by batch effects (Additional file 2: Figure S1A-B). To address this and identify the most efficient strategy to minimize batch effects while preserving biologically relevant variability, we evaluated five combinations of normalization techniques along with batch effect adjustment methods: (i) Quantile normalization, a common technique in high-throughput data analysis, such as genomics, proteomics, or transcriptomics, which aids in equalizing intensity and signal distributions among different samples [62]; (ii) PamR, which employs analysis of variance to identify and model patterns associated with batch effects, subsequently separating and mitigating these non-biological influences from the data [45]; (iii) an enhanced version of the PamR technique that combines QN to adjust each column of the m6A profiles matrix, incorporating it within the PamR approach; (iv) the Combined Batch Adjustment Tool (ComBat) method, an empirical Bayes batch regression technique elaborated by Johnson and collaborators, often employed by researchers to harmonize the RNAseq profiles. The ComBat method is particularly useful after scale adjustments have been performed independently for each gene within different batches [14, 32]; (v) we implemented a correction method that combines QN with ComBat, a method introduced by O’Rourke et al., for a Transcriptome Data methodology comparison [32]. Li, et al. proposed this pipeline approach in the online software named m6Acorr, explicitly designed to mitigate biases in m6A microarrays datasets [24]. These methods were evaluated head-to-head by RLE plot and PCA and are illustrated in Additional file 2: Figure S1. Additional file 2: Figure S1C-H illustrates the distribution before and after normalization, while PCA (Additional file 2: Figure S1I-N) exhibits distinct clustering patterns among the samples after normalization and batch correction. Combining quantile normalization with ComBat methodology effectively better reduced batch effects as evidenced by the elimination of hierarchical clustering by batch (Additional file 2: Figure S1O) while preserving biological variability of gene ranks. Similar results were obtained for the gene expression profile (Additional file 2: Figure S2). Our findings that quantile normalization plus ComBat produced optimal normalization and batch correction are in line with those presented by O’Rourke and Li [24, 32]. We conclude that combined quantile normalization with ComBat methodology removes batch effects in our m6A analysis.

### m6A methylation analysis of first resection GB tissue identifies clusters with increased purity of PD and PsPD

To determine the extent to which m6A methylation status could identify the diagnostic groups PD and psPD from the first resection specimen, we evaluated the percent methylated RNA at m6A in our patient cohorts. We evaluated this by hierarchical clustering and validated the significance through Fisher’s exact test. The general approach is shown in Fig. 2 . Through hierarchical clustering of percent methylated transcripts, we note that three general clusters can be identified with varying levels of diagnostic label purity. Distributions of PD and psPD for the different clusters were as follows: cluster 1 = 70% PD and 30% psPD; cluster 2 = 100% PD and 0% psPD, and cluster 3 = 34% PD and 66% psPD (Fig. 2B). Analysis by Fisher’s exact test showed a statically significant difference in diagnostic label by cluster (*p* = 1.59e-06) (Fig. 2C), Platelets (*p* = 0.021) and EGFR Amplification (*p* = 0.003), while various clinical and histopathologic variables did not differ statistically between clusters (Additional file 3: Figure S3). We also evaluate the hierarchical cluster performance using the silhouette plot (0.102) and ARI metric (0.162) demonstrating the consistence in the clustering performance (Fig. 2E). While using Cohort A or B as training and testing sets for the assessment of the stability and reproducibility of clusters across different populations (Fig. 2D). The RMSE, ARMSE, MAPE and CVIM metrics, all near zero, confirm the validity of the approach (Fig. 2F). Figure 2G show the distribution across the three clusters. This comparison confirms significant differences in diagnostic label purity beyond what would be expected by chance, indicating that epitranscriptional modification of the initial resection specimens is distinct in patients that evolve to PD versus psPD.

### m6A-associated methylation variability reveals gene regulation through RNA polymerase II in GB transcripts

**Fig. 3:**
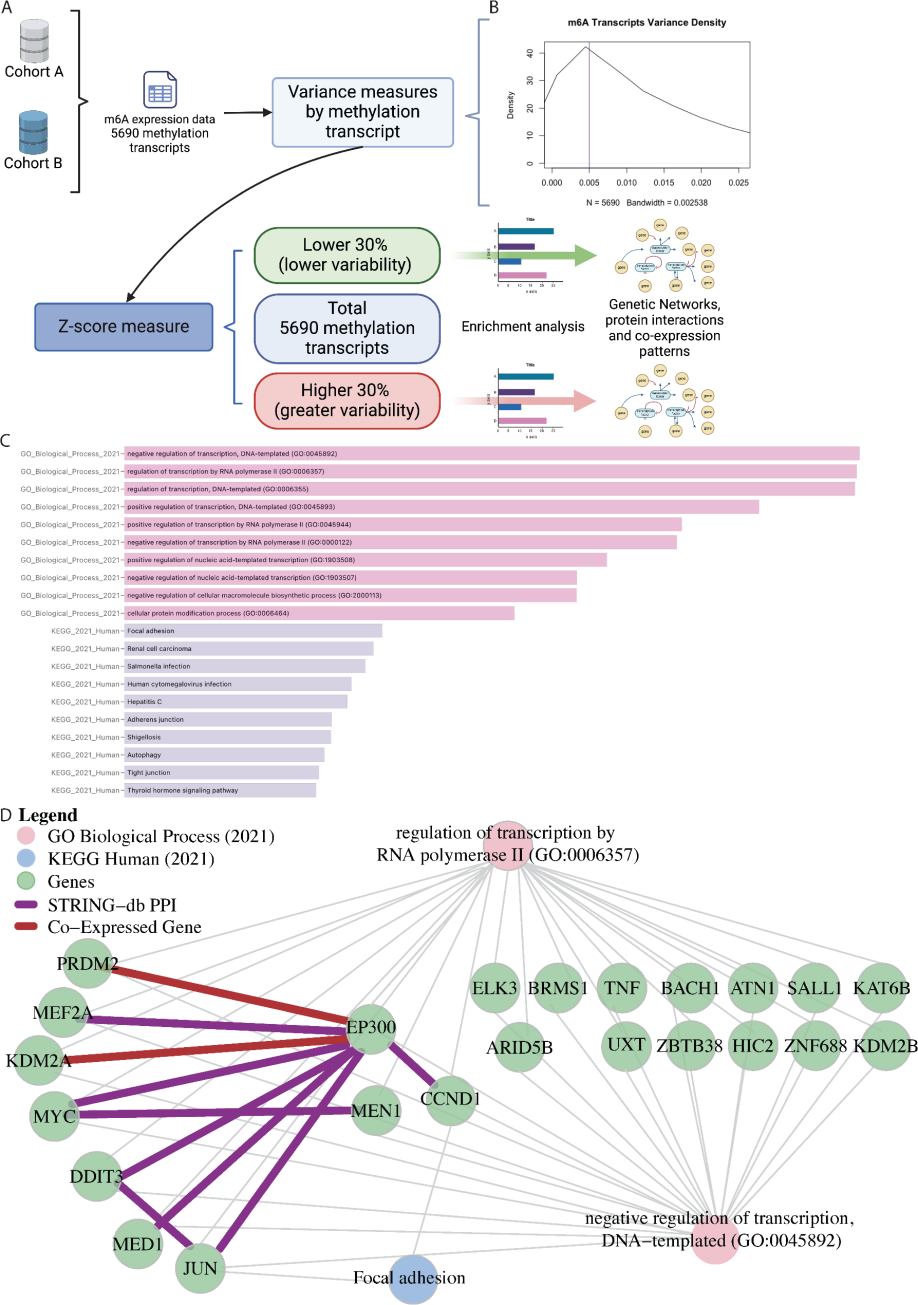
Enrichment representation of top biological pathways. (A) workflow exploring the variance within methylated transcripts and delineate their impact on biological pathways. (B) density plot showcasing the distribution of variance among methylated transcripts. (C) A bar graph is presented illustrating the most highly enriched GO and KEGG pathways, ranked according to their p-values (p < 0.05). (D) A specific subnetwork is depicted, comprising of 23 critical genes, placing emphasis on the two primary GO pathways and the most prominent KEGG pathway((D(

Clustering of our samples is driven by m6A transcript variance. However, as reviewed by Wolf, et al. [54], genes with low variance across samples are often associated with basal cell processes and tend to be associated with unique heterochromatin states. Thus, additional biological insights can be obtained from analyses low variance transcripts. To our knowledge, no documentation of low variance m6A transcripts have been reported in GB, permitting, for the first time, to explore potentially core biological processes common to all GB tumors. To further explore the variation in our 5690 m6A methylated transcripts data in our entire GB population (including both PD and psPD patients), we calculated Z-scores of transcript variance on Fig. 3A followed by an enrichment analysis to reveal distinct genetic networks and associated pathways. Additional file 4: Figure S4A-C shows the variation results for all variance before Z-score analysis. This approach effectively categorized methylated transcripts into two distinct groups based on their variability levels. The transcripts within the lower 30%’tile of Z scores indicates consistent methylation patterns across all patients therefore revealing a gene set of methylated transcripts with low m6A variability. Conversely, transcripts in the highest 30%’tile of Z scores indicated greater variability across all patients. To our knowledge, no documentation of the low variance m6A transcripts have been reported in GB. The lowest 30%’tile of m6A variance (Fig. 3B-C) showed enrichment for a focused subnetwork comprising 23 genes. This network displays interactions among key genes such as EP300, JUN, CCND1, amongst others, with significant protein-protein interactions identified through the STRING-db database. Notably, co-expression patterns between EP300 and KDM2A, as well as PRDM2 and EP300, emphasize functional relationships within these biological processes. After our modified enrichment analysis we demonstrated associations primarily within the processes of “regulation of transcription, DNA-templated” and “regulation of transcription by RNA polymerase II” as defined by the GO database (GO: 0006355 and 0006357) and included the following genes: EP300, PEDM2, MEF2A, KDM2A, MYC, DDIT3, MED1, JUN, MEN1, CCND1, ELK3, ARID5B, BRMS1, TNF, UXT, BACH1, ZBTB38, ATN1, HIC2, SALLI, ZNF688, KAT6B and KDM2B. Furthermore, analysis of KEGG data identified the “focal adhesion” pathway as an enriched pathway.

In contrast, the highest 30%’tile of variance (Additional file 4: Figure S4B-D) comprised a larger subnetwork consisting of 52 genes, showing protein-protein interactions among genes such as MYC, SMAD3, and CAV1, along with multiple co-expressions such as MAML1 with SRCAP and HCFC1, highlight a distinct set of genetic interactions and pathways within this group. The KEGG enrichment included ARRB1, CAV1 and SMAD3, constituents of the “Endocytosis” pathway. In this gene set of high m6A variance, we also identified a GO pathway enrichment for GO: 0006357 and 0006355. Although these are the same GO pathways identified in the low m6A variance gene, the genes that created the pathway enrichment were distinct from the genes of the low variance m6A gene set. This gene set is illustrated in Additional file 4: Figure S4D. We conclude that regulators of transcriptional processes undergo marked m6A regulation relative to other metabolic pathways in GB. Our data also suggest that some transcriptional regulators constitute a core pathway under tight m6A regulation in GB.

### Complex relationships exist in the context of Site-Specific m6A RNA methylation

The gene set of our probes that are queried include a large quantity of transcript that contain more than one probed ACA site for m6A analysis. This offered us the opportunity to evaluate how correlated the methylation on one site would be relative to another site on the same gene product. To achieve this, we performed Pearson’s correlations and linear modelling of the methylation at one m6A site to other m6A sites of the same transcript, which we deem intra-transcript m6A correlations. These data are tabulated in Additional file 1: Table S3. For instance, the RNA metabolism gene AGO2 shows a Pearson’s correlation of 0.55 of the sites at 2591 and 2960 (p value < 0.001). This weakly positive correlation implies that both sites in general undergo m6A modification concurrently in GB patients. ADNP2 has an intra-transcript m6A Pearson’s correlation of 0.17 of the sites at 1698, 3300 and 2989 (p value < 0.001), indicating that these sites are methylated independent of each other. DNAAF5’s Pearson’s correlation equals − 0.56 of sites at 2746 and 2808 (p value < 0.001), indicates that these sites are rarely concurrently methylated. These data suggest that m6A modification on different sites of the same gene can be co-regulated, independent, or dependent of each other. 

### Epitranscriptomic profiling reveals module-specific gene expression signatures associated with PD vs. psPD

**Fig. 4:**
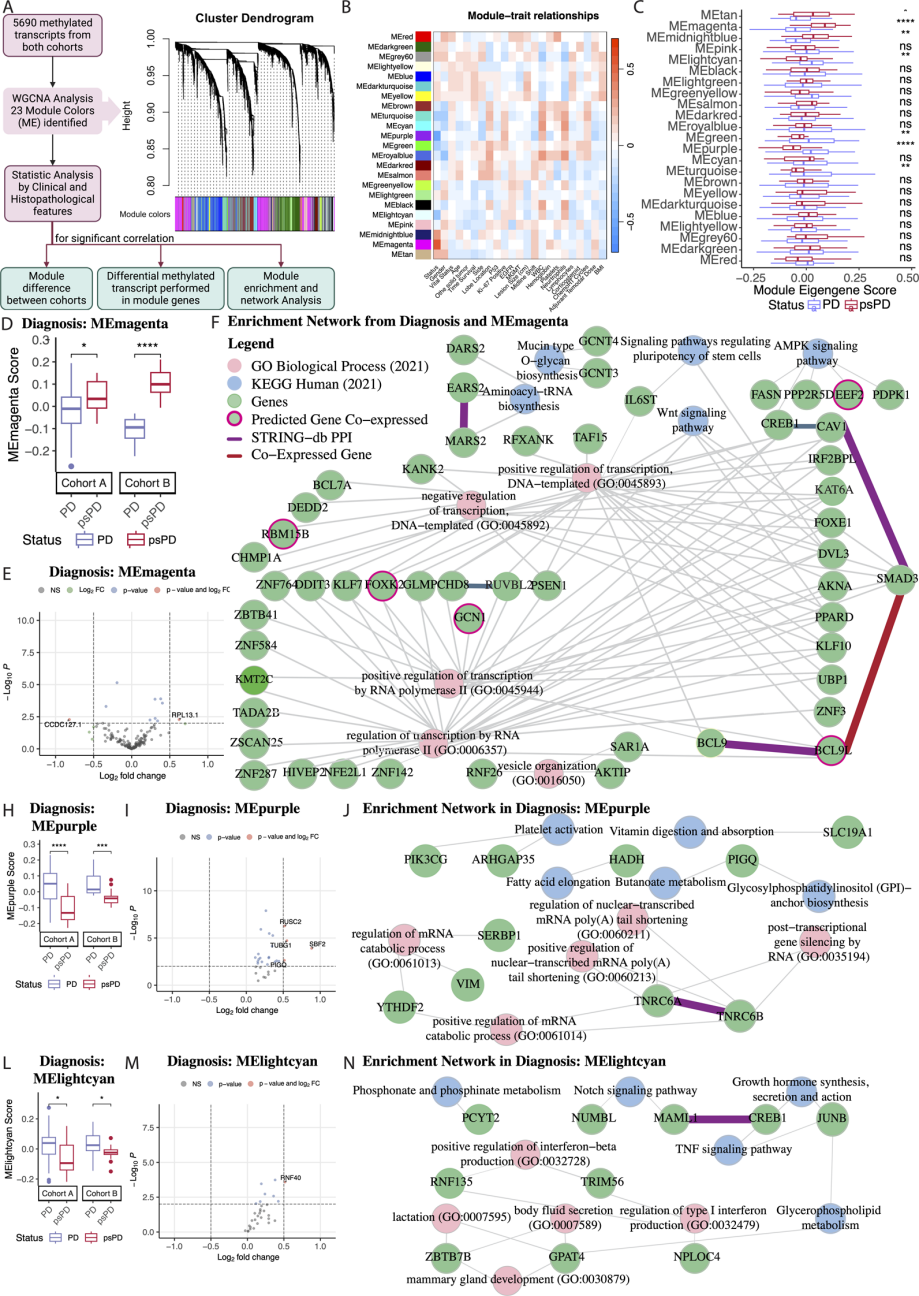
Analytical workflow employed in the WGCNA. (**A**) Workflow and Cluster Dendrogram derived from WGCNA, revealing the subdivision of genetic data into 23 distinct modules represented by unique colors. (**B**) Heatmap detailing the correlation patterns between these modules and various clinical and histopathological traits of the patients. (**C**) Boxplot compares Diagnostic PD versus psPD by each module colors. (**D**,** H**, and **L**) illustrate the differences in each Cohort A or Cohort B batch analyses of diagnostic data compared to modules colored in magenta, purple, and light cyan, respectively. (**E**, **I**, and **M**) highlight genes displaying differential methylation patterns within the magenta, purple, and light cyan modules, respectively. (**F**,** J**, and **N**) represent the enriched gene-gene associations and showcase the top 5 GO processes and KEGG pathways within augmented networks specific to the magenta, purple, and light cyan modules, respectively. All pathways present p-value < 0.05. Initial comparisons were screened by ANOVA and t test were used for specific groupings and significant effects are indicated by *p < 0.05; **p < 0.01; ***p < 0.001; **** *p* < 0.0001

Recent studies have demonstrated that WGCNA followed by DEG analysis results permits functional enrichment and network analysis that enables superior biological insight [44]. Furthermore, to our knowledge, we have not identified neuropathological studies that have shown a tendency for DMT genes to show functional enrichment in correlational approaches such as those deployed in a WGCNA. WGCNA has enabled identification of co-expression modules among genes, revealing functional clusters, and has provided invaluable insights for functional and clinical studies using genomic datasets. Figure 4A shows the cluster dendrogram demonstrating that our m6A RNA methylated data segregates into 23 distinct modules. This delineation offered a comprehensive overview of the patterns of gene co-expression in different clinical contexts. Additionally, the heatmap was used to illustrate the correlation between these modules and different clinical parameters, presenting the correlation patterns between genetic modules and clinical phenotype/trait (Fig. 4B). We note that the module eigengene (ME) magenta showed the highest correlation to the psPD clinical phenotype/trait, whereas module eigengenes MEpurple and MElightcyan were more correlated with PD clinical phenotype/trait (Fig. 4C). Furthermore, comparisons between Cohort A and Cohort B present significant differences when analyzed as separate batches (Fig. 4D, H, L). We present data between patient groups based on other clinical parameters in Additional file 5: Figure S5F, I, L and O. Our analytical pipeline focused on seven significantly distinct modules correlated with clinical phenotype/traits of PD versus psPD: MEmagenta, MEpurple, MElghtcyan, MEmidnightblue, MEgreen, MEturquoise, and MEtan. Furthermore, a comprehensive analysis of the clinical and histopathological factors, including age, life status, BMI (body mass index), other tumors, and MGMT, was conducted to elucidate any relationship between these patient groups within each module (Additional file 5: Figure S5A-E). We also document associations among clinical and histopathological factors—such as Gender, Time Survival, Midline Shift, Side, Lobe Location, Platelets, Lymphocytes, Hemoglobin, White Blood Count (WBC), EGFR, Ki-67, and p53 expression by immunohistochemistry. These associations did not meet our threshold of significance in both Cohort A and Cohort B (Additional file 5: Figures S5-10 and Additional file 1: Table S4). To validate this first-time methylated profile based on ACA-based RNA endonuclease digestion, we performed the Boruta algorithm analysis to identify the methylated transcripts in module magenta. Magenta Module contain 154 methylated transcripts can predict 1510 gene in the gene expression data. Selecting the identified confirmed genes expressed in the Boruta algorithm results, we performed the heatmap and GO analysis (Additional file 5: Figures S11 and Additional file 1: Table S4), confirming our results for the WGCNA analysis. We conclude that WGCNA workflows demonstrate functional enrichment of correlated methylated transcripts. We further conclude that at the first resection, patients who ultimately present with PD versus psPD show different levels of methylation of transcripts implicated in RNA metabolic functional pathways.

### m6A-Associated differentially methylated transcripts (DMTs) reveals regulation of histone H2B ubiquitination in GB

**Fig. 5:**
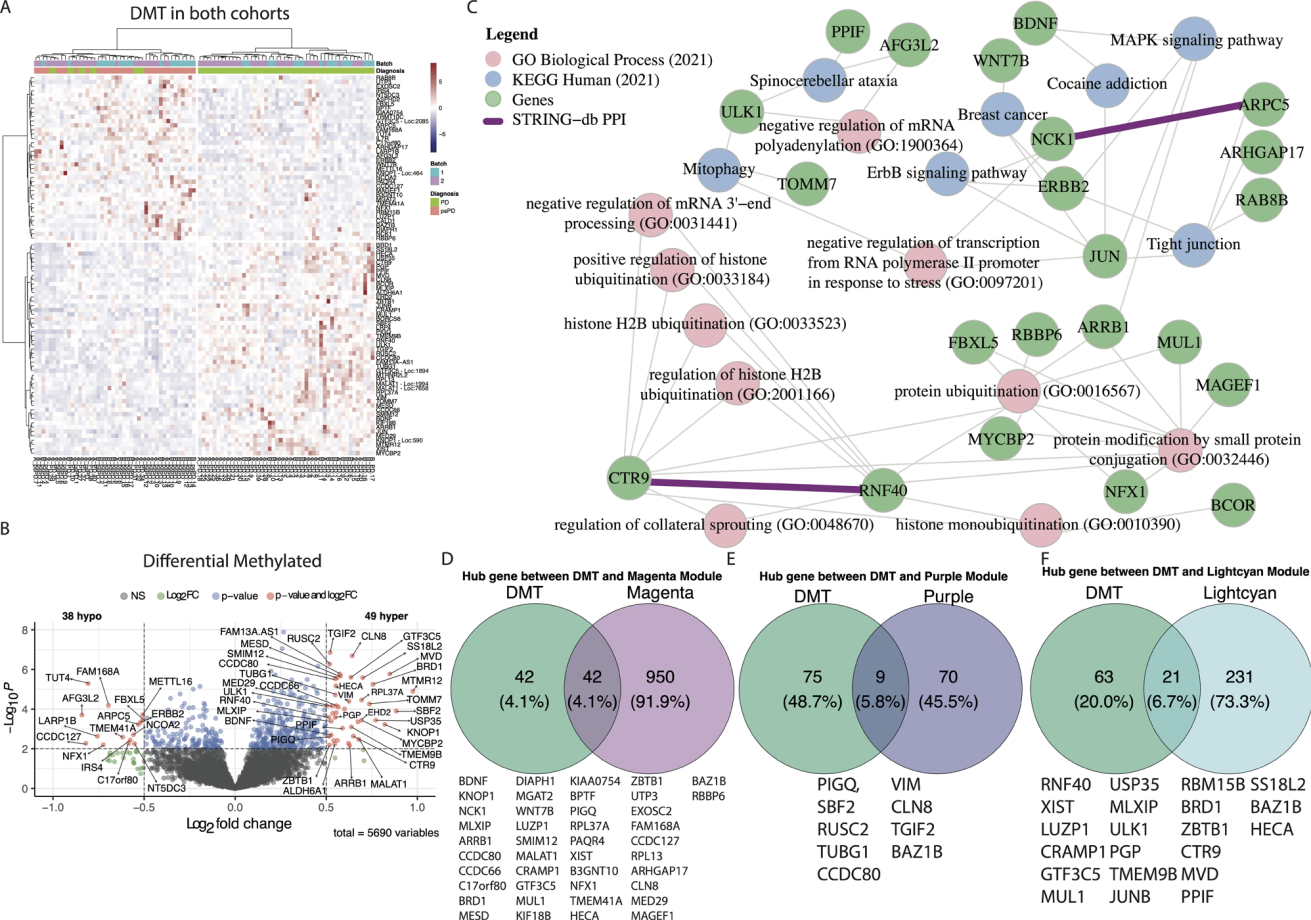
Comprehensive insight into differential methylation transcripts. Figure (**A**) showcases a heatmap depicting the distribution of 87 DMTs among patients. (**B**) the volcano plot delineates 38 hypomethylated and 49 hypermethylated DMTs. (**C**) Demonstrates a network diagram featuring key genes (depicted as green circles) with hyper- or hypomethylated status, their gene co-expressions (indicated by red lines), or protein-protein interactions (indicated by purple lines). These genes are associated with the top eight GO pathways (depicted as pink circles) and KEGG pathways (depicted as blue circles). All pathways present p-value < 0.05. Venn diagram showing the hub genes between DMT and (**D**) Magenta, (**E**) Purple, and (**F**) Lightcyan module

We next aimed to determine which m6A RNA methylated transcripts were correlated to functional outcomes in the PD group. To explore the distribution of DMTs among PD versus psPD patients, we employed differential analysis in the methylated transcripts and visualized the results by heatmap organized by hierarchical clustering as well as a volcano plot. The distribution pattern and directionality of 87 DMTs are presented in Fig. 5A by three clusters in the heatmap and in Fig. 5B by PD and psPD groups in the volcano plot. Of these, 38 DMT were identified as hypomethylated in PD, while 49 DMT were hypermethylated in PD.

To establish the biological functional significance of the observed methylation changes, we carried out a modified GO and KEGG enrichment analysis as delineated in the Methods section, the results of which are illustrated in Fig. 5D. The GO enrichment reveals various significant associations emerged such as ULK1 (Hyper) and AFG3L2 (Hypo) influencing “regulation of mRNA polyadenylation (GO: 1900364)”; CTR9 (Hyper) and RNF4 (Hyper) affecting “regulation of collateral (axonal) sprouting (GO:0048670)”, “negative regulation of mRNA 3’-end processing (GO:0031441)”, and histone ubiquitination process (GO:0033184, 0033523 and 2001166). Importantly, the findings from the GO analysis were confirmed by protein-protein interaction (PPI) inference data from the STRING-db, which revealed a direct physical interaction between RNF40 and CTR9. By broadening our perspective to incorporate the KEGG pathways, we also observed that ERBB2 (Hypo), NCK1 (Hypo), and JUN (Hyper) are members of the ErbB signaling pathway, while BDNF (Hyper) and JUN (Hyper) are constituents of the Cocaine addiction pathway. We illustrate by Venn Diagram the overlap between DMT and genes composing modules magenta, purple, and lightcyan (Fig. 5D-F). The magenta module is correlated with psPD diagnosis are more related with genes involved in “Various types of N-glycan biosynthesis” and “MAPK signaling pathway” KEGG pathway while the purple and lightcyan module is correlated with PD diagnosis. The DMT in purple module are involved in “regulation of mRNA catabolic” GO process while DMT in lightcyan module are more involved in “Histone ubiquitination” GO process and “Tight junction” KEGG pathway. We conclude that analysis of the original resection of GB patients shows distinct RNA regulation and ubiquitination pathways that correlate with psPD and PD diagnoses later in their clinical course.

### Hyper m6A modulated transcripts correlate with high expression genes of key metabolic processes in PD GB patients

**Fig. 6:**
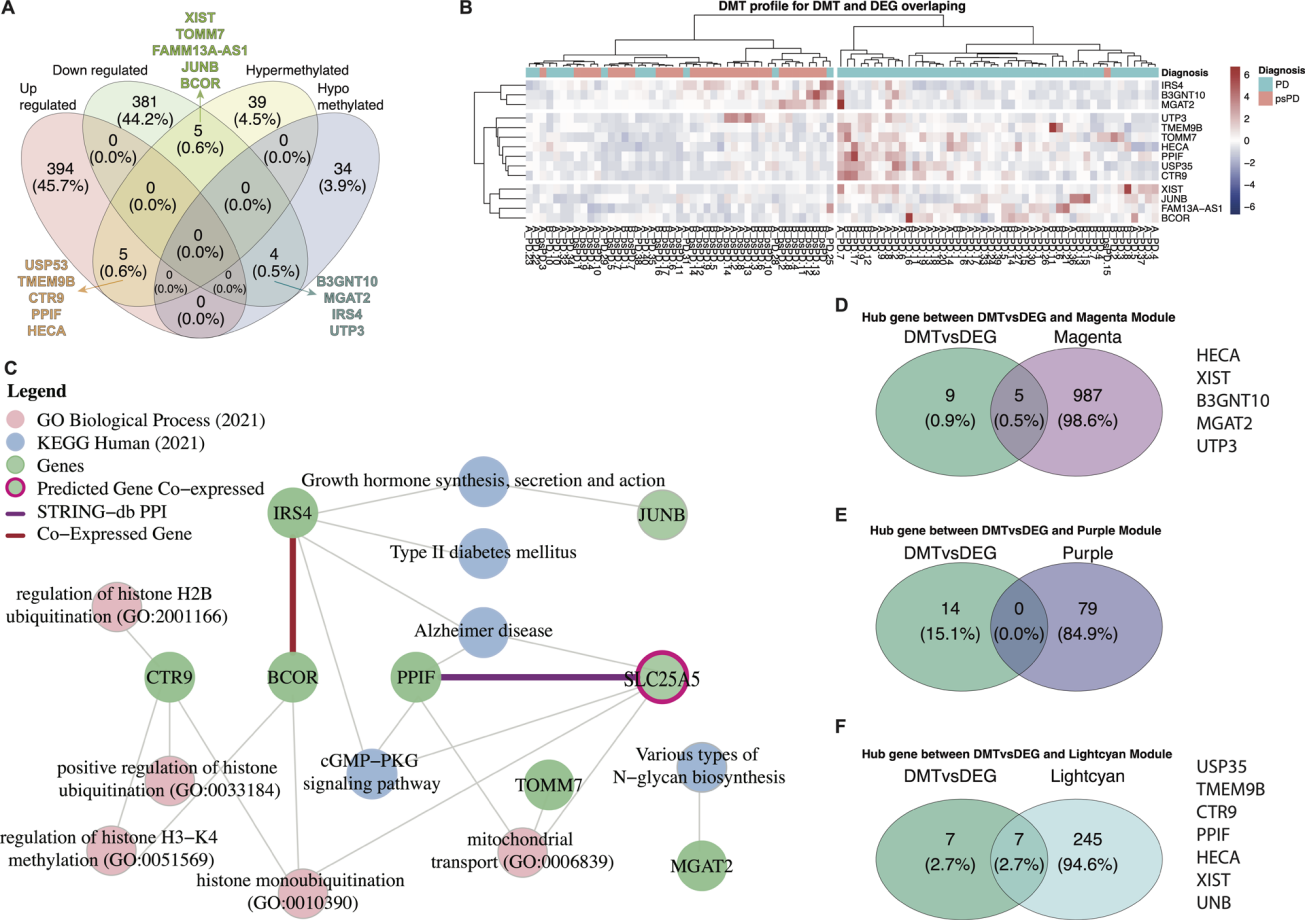
Integrated analysis of gene expression and DNA methylation. (**A**) Venn diagram illustrates the overlap of differentially expressed genes with hyper- or hypomethylated status. (**B**) Depicts a heatmap displaying differentially methylation patterns of the 14 genes that are overlapping DMT and DEG. Matrix represents the m6A percentage 0 = 0% to 5 = 1200%. (**C**) Demonstrates a network diagram featuring key genes (depicted as green circles) with hyper- or hypomethylated status, their gene co-expressions (indicated by red lines), or protein-protein interactions (indicated by purple lines). These genes are associated with the top eight GO pathways (depicted as pink circles) and KEGG pathways (depicted as blue circles). Additionally, a node representing another gene (depicted as a green circle with a red border) not included in our primary analysis is shown, which typically exhibits co-expression with PPIF. All pathways present p-value < 0.05. Venn diagram showing the hub genes between DMT and (**D**) Magenta, (**E**) Purple, and (**F**) Lightcyan module

Addressing the interplay between gene expression and m6A RNA methylation, we performed a co-joint analysis to delineate the intersections between DEG and DMT. The Venn diagram show 789 DEGs and 87 DMTs in PD and psPD patients (Fig. 6A). We note that the majority of the DMT genes are not differentially expressed at our threshold of significance, indicating that transcriptional regulation and epitranscriptional regulation may provide two different modes of gene expression control. Of the remaining that were both DMT and DEG at our threshold of significance, 14 transcripts overlapped in the following manner: 5 hypermethylated/downregulated, 4 hypomethylated/downregulated, and 5 hypermethylated/upregulated genes (Fig. 6A). These data are visualized in the heatmap detailing the expression patterns (Fig. 6B). To unravel biological implications, we identified top GO and KEGG pathways associated with DEGs and DMTs intersection, as illustrated in the network diagram (Fig. 6C) providing significant associations across diverse molecular processes. Our modified GO enrichment analysis showed enrichments for “cell fate commitment involved in formation of primary germ layer (GO:0060795)” associated with CTR9 (Hyper/Up) and “negative regulation of cellular respiration (GO:1901856)” related with PPIF (Hyper/Up). Our KEGG pathway analysis indicated that PPIF (Hyper/Up) physically interacts with SLC25A5 (STRING-db PPI) and is implicated in the cGMP-PKG signaling pathway and Alzheimer’s disease pathway. Additionally, the Venn Diagram show the hub genes relation with the magenta (5 genes), and lightcyan (7 genes) modules (Fig. 6D-F). The magenta module is correlated with psPD diagnosis are more related with genes that are Hypomethylated and down-regulated and involved in “Varios types of N-glycan biosynthesis” KEGG pathway while the lightcyan module is correlated with PD diagnosis are more related with Hypermethylated genes in up-regulated genes and involved in “Histone ubiquitination” GO process and “Growth hormone synthesis, secretion and action” KEGG pathway. We conclude that for most transcripts in GB, that differential methylation is regulated distinctly from gene expression changes. We also conclude that in genes that undergo both differential methylation and differential gene expression, that these genes are often implicated in metabolic processes.

So as to validate and compare the clustering consistency and performance between different sets of data derived from the same samples, we compared the hierarchical clustering from DMT landscape (87 genes) within the samples with the new hierarchical clustering based on an overlapping set from DMT with DEG (14 genes) (Additional file 6: Figure S12). The PCA plots and metric comparisons enable us to visually and quantitatively assess the alignment and segregation of clusters within each dataset, as well as across the combined dataset. This approach allows us to establish a connection between the methylation profiles and gene expression patterns, thereby highlighting the biological relevance and reliability of the hierarchical clustering results. Ultimately, this comprehensive analysis ensures that the identified clusters are not artifacts of a single data type but are reflective of underlying biological phenomena captured by both methylation and expression data.

## Discussion

Our analysis was focused on evaluating samples from patients designated of psPD or PD by the neuro-oncologist. As these patients needed to survive to the point where a contrast enhancing lesion on the MRI generated a psPD/PD differential diagnosis, our inclusion criteria biased these data towards a subset of patients with longer survival relative to the entire GB population. Our inclusion criteria would exclude therefore patients showing so-called rapid early progression (REP) in GB from our study cohort. REP, characterized by recurrent tumor growth on MRI scans detected in the interval from the post-operative MRI (typically utilized to evaluate the extent of gross total resection) to the pre-radiotherapy MRI (typically utilized for radiation planning), represents an aggressive disease phenotype associated with poor prognosis and shorter survival times [35, 51]. Future studies exploring m6A analysis on an REP versus non-REP population are under way.

Our study is the first to our knowledge implementing ACA-based RNA endonuclease digestion to evaluate RNA methylation in Glioblastoma. This ACA-based digestion we found to be fully compatible with a spectrum of archival pathology samples ranging in FFPE storage time of 7–8 years. Our bioinformatics workflow enabled us to describe several novel aspects of GB epitranscriptome. Though hierarchical cluster analysis of %methylation we can identify three clusters with significant enrichment of PD or psPD (Fig. 2B). Wen, et al. delineates some inclusion/exclusion criteria for the designation of PD versus psPD [53]. However, objective, quantifiable biomarkers to distinguish these two entities has eluded the neuropathology community. Furthermore, there is debate within the neuro-oncology clinical community regarding the extent to which PD and psPD patients have ultimately distinct etiologies and mechanisms underlying their disease progression (see, Jia et al., for review and perspective [12]). Our findings that distinct RNA metabolic pathways are enriched AND detectable at the point of the first resection supports conjectures proposed in Jia et al. [12]and Nishino et al. [31], amongst others, that PD and psPD patients may have distinct underlying etiologies and clinical evolution.

Through application of WGCNA, we were able to identify module of % methylated transcripts that showed enrichment in PD versus psPD (Fig. 4). Of note, the GO enrichment of the % methylated transcript modules were primarily of RNA regulation pathways, indicating that methylation regulation may the distinct in patients who evolve a PD versus psPD course. The post-transcriptional m6A RNA modification mechanism has emerged as an important regulator in the formation of oncometabolites in different cancer types, including glioblastoma. Recent research has emphasized the impact of m6A on the control of gene expression linked to metabolic pathways that produce oncometabolites like 2-hydroxyglutarate (2HG), glutamine, succinate, fumarate, fatty acid synthesis, cholesterol metabolism, which are essential molecules involved in Warburg effect, nucleotide synthesis, redox balance, and epigenetic regulation metabolism, which contribute significantly to cell proliferation, growth and survival [8, 22, 25–27].

Our study identified important biological processes regulated by m6A RNA methylation associated with cell metabolism alterations beyond distinguishing GB patients that have PD versus psPD. Some of these transcripts encode enzymes that were related to promotion of 2HG oncometabolite affecting redox homeostasis by MAPK and PI3K/Akt pathways in mitochondria, negatively related with potential activation of ErbB signaling pathway, and influence cell differentiation in GB. The redox environment can change the glycolytic flux leading to glucose reduction, and lactate and fumarate elevation regulating the Warburg effect indirectly affecting the regulation of cellular respiration in GB [39]. These pathways are crucial for cellular signaling processes integral to cancer progression, survival, and treatment resistance [1, 11, 17, 29, 36, 38, 47]. Ultimately, in GB contexts, various processes like mRNA polyadenylation, 3’-end, collateral (axonal) sprouting, and histone ubiquitination are regulated through downstream effectors such as MAPK and PI3K/Akt pathways. This could impact gene transcription and epigenetic regulation in RNA processing, synaptic plasticity, which potentially modify chromatin structure and cellular metabolism [6, 16, 20, 28, 30, 37, 41, 42, 56, 59].

Our data further provide insights into m6A modification in GB cells specifically, we demonstrate that transcripts with more than one m6A site can have positive, negative or no correlation, suggesting that some transcripts can be coregulates undergo m6A modification independently or dependently. We further note that the differential expression of the genes usually is not reflected by changes in m6A methylation. This permits a non-transcriptional mechanism by which key metabolic genes can be further regulated in GB.

## Conclusion

In conclusion, our study delves on potential implications of these findings and their extension to targeted interventions, envisioning the development of innovative m6A-based diagnostic tools and therapeutic targets for managing glioblastoma. We highlight distinct hub co-methylated transcripts related to cancer metabolism intricately linked to PD versus psPD phenotypes/traits in GB, impacting on disease progression. Importantly, our results emphasize the importance of future research efforts to embrace epitranscriptome and metabolome integration, roles of non-coding RNAs, and strategically designed clinical trials. These initiatives are crucial steps towards translating emerging molecular insights into transformative therapeutic approaches, contributing neuropathology diagnosis and neuro-oncology decision underlying PD versus psPD patients in GB.

## Electronic supplementary material

Below is the link to the electronic supplementary material.

Supplementary Material 1

Supplementary Material 2

Supplementary Material 3

Supplementary Material 4

Supplementary Material 5

Supplementary Material 6

Supplementary Material 7

## Data Availability

Full code and packages used for R analysis can be found on Github: <https://github.com/GlauciaMMFernandes/m6A-landscape-Glioblastoma-subgroups>.

## Abbreviations

GB: Glioblastoma, IDH-wild type
IDH: Isocitrate dehydrogenases family
CNS: Central nervous system
chemoRT: Radiotherapy, and chemotherapy
MRI: Magnetic resonance imaging
PD: Progressive disease, genuine cancer progression
psPD: Pseudo-progressive disease
m6A: N6-methyladenosine
lncRNAs: Long non-coding RNAs
miRNAs: Micrornas
circRNAs: Circular RNA
MGMT: O6-methylguanine-DNA methyltransferase
WHO: World health organization
FFPE: Formalin-fixed paraffin-embedded
QN: Quantile normalization
PamR: Pam: prediction analysis for microarrays
ANOVA: Analysis of variance
ComBat: Combatting batch effects when combining batches of microarray
SVA: Surrogate variable analysis
DEG: Differentially expressed genes
DMT: Differentially m6a RNA methylated transcripts
log2FC: Log 2-fold change
GO: Gene ontology
KEGG: Kyoto Encyclopedia of Genes and Genomes
WGCNA: Weighted gene correlation network analysis
STRING-db: Protein-protein interaction networks functional enrichment analysis
PPI: Protein-protein interaction
EGFR: Epidermal growth factor receptor
EP300: E1a binding protein p300
JUN: Jun proto-oncogene, AP-1 transcription factor subunit
CCND1: Cyclin D1
KDM2A: Lysine demethylase 2 A
PRDM2: PR/SET domain 2
ME: Module eigengene
WBC: White blood count
Ki-67: Antigen identified by monoclonal antibody Ki 67
p53: Tumor protein p53
BMI: Body mass index
REP: Rapid early progression
2HG: 2-hydroxyglutarate
MAPK: Mitogen activated kinase-like protein
PI3K: Phosphatidylinositol 3-kinase, putative
Akt: Akt kinase
ErbB: Erb-b2 receptor tyrosine kinase

## References

[1] Alowaidi F, Hashimi SM, Alqurashi N, Wood SA, Wei MQ (2019) Cripto-1 overexpression in U87 glioblastoma cells activates MAPK, focal adhesion and erbb pathways. Oncol Lett 18:3399–3406. 10.3892/ol.2019.10626 31452820 10.3892/ol.2019.10626 PMC6676405

[2] Ashburner M, Ball CA, Blake JA, Botstein D, Butler H, Cherry JM, Davis AP, Dolinski K, Dwight SS, Eppig JT, Harris MA, Hill DP, Issel-Tarver L, Kasarskis A, Lewis S, Matese JC, Richardson JE, Ringwald M, Rubin GM, Sherlock G (2000) Gene ontology: tool for the unification of biology. The gene ontology consortium. Nat Genet 25:25–29. 10.1038/75556 10802651 10.1038/75556 PMC3037419

[3] Barbaro M, Blinderman CD, Iwamoto FM, Kreisl TN, Welch MR, Odia Y, Donovan LE, Joanta-Gomez AE, Evans KA, Lassman AB (2022) Causes of death and End-of-Life care in patients with intracranial High-Grade gliomas: A retrospective observational study. Neurology 98:e260–e266. 10.1212/WNL.0000000000013057 34795049 10.1212/WNL.0000000000013057 PMC8792811

[4] Benavides-Serrato A, Saunders JT, Kumar S, Holmes B, Benavides KE, Bashir MT, Nishimura RN, Gera J (2023) m. Cancer Lett 562:216178. 10.1016/j.canlet.2023.216178 37061119 10.1016/j.canlet.2023.216178 PMC10805108

[5] Blighe KRS, Lewis M (2023) EnhancedVolcano: Publication-ready volcano plots with enhanced colouring and labeling. 10.18129/B9.bioc.EnhancedVolcano

[6] De Silva MI, Stringer BW, Bardy C (2023) Neuronal and tumourigenic boundaries of glioblastoma plasticity. Trends Cancer 9:223–236. 10.1016/j.trecan.2022.10.010 36460606 10.1016/j.trecan.2022.10.010

[7] Evangelista JE, Xie Z, Marino GB, Nguyen N, Clarke DJB, Ma’ayan A (2023) Enrichr-KG: bridging enrichment analysis across multiple libraries. Nucleic Acids Res 51:W168–W179. 10.1093/nar/gkad393 37166973 10.1093/nar/gkad393 PMC10320098

[8] Fitzsimmons CM, Mandler MD, Lunger JC, Chan D, Maligireddy SS, Schmiechen AC, Gamage ST, Link C, Jenkins LM, Chan K, Andresson T, Crooks DR, Meier JL, Linehan WM, Batista PJ (2024) Rewiring of RNA methylation by the oncometabolite fumarate in renal cell carcinoma. NAR Cancer 6:zcae004. 10.1093/narcan/zcae004 38328795 10.1093/narcan/zcae004 PMC10849186

[9] Goh WWB, Wang W, Wong L (2017) Why batch effects matter in omics data, and how to avoid them. Trends Biotechnol 35:498–507. 10.1016/j.tibtech.2017.02.012 28351613 10.1016/j.tibtech.2017.02.012

[10] Heumos L, Schaar AC, Lance C, Litinetskaya A, Drost F, Zappia L, Lücken MD, Strobl DC, Henao J, Curion F, Schiller HB, Theis FJ, Consortium S-cBP (2023) Best practices for single-cell analysis across modalities. Nat Rev Genet 24:550–572. 10.1038/s41576-023-00586-w 37002403 10.1038/s41576-023-00586-w PMC10066026

[11] Huang L, Li X, Ye H, Liu Y, Liang X, Yang C, Hua L, Yan Z, Zhang X (2020) Long non-coding RNA NCK1-AS1 promotes the tumorigenesis of glioma through sponging microRNA-138-2-3p and activating the TRIM24/Wnt/β-catenin axis. J Exp Clin Cancer Res 39:63. 10.1186/s13046-020-01567-1 32293515 10.1186/s13046-020-01567-1 PMC7158134

[12] Jia W, Gao Q, Han A, Zhu H, Yu J (2019) The potential mechanism, recognition and clinical significance of tumor pseudoprogression after immunotherapy. Cancer Biol Med 16:655–670. 10.20892/j.issn.2095-3941.2019.0144 31908886 10.20892/j.issn.2095-3941.2019.0144 PMC6936240

[13] Jiang X, Liu B, Nie Z, Duan L, Xiong Q, Jin Z, Yang C, Chen Y (2021) The role of m6A modification in the biological functions and diseases. Signal Transduct Target Ther 6:74. 10.1038/s41392-020-00450-x 33611339 10.1038/s41392-020-00450-x PMC7897327

[14] Johnson WE, Li C, Rabinovic A (2007) Adjusting batch effects in microarray expression data using empirical Bayes methods. Biostatistics 8:118–127. 10.1093/biostatistics/kxj037 16632515 10.1093/biostatistics/kxj037

[15] Karatzas E, Gkonta M, Hotova J, Baltoumas FA, Kontou PI, Bobotsis CJ, Bagos PG, Pavlopoulos GA (2021) VICTOR: A visual analytics web application for comparing cluster sets. Comput Biol Med 135:104557. 10.1016/j.compbiomed.2021.104557 34139436 10.1016/j.compbiomed.2021.104557

[16] Kersh AE, Sasaki M, Cooper LA, Kissick HT, Pollack BP (2016) Understanding the impact of erbb activating events and signal transduction on antigen processing and presentation: MHC expression as a model. Front Pharmacol 7:327. 10.3389/fphar.2016.00327 27729860 10.3389/fphar.2016.00327 PMC5052536

[17] Khabibov M, Garifullin A, Boumber Y, Khaddour K, Fernandez M, Khamitov F, Khalikova L, Kuznetsova N, Kit O, Kharin L (2022) Signaling pathways and therapeutic approaches in glioblastoma multiforme (Review). Int J Oncol 60. 10.3892/ijo.2022.5359 10.3892/ijo.2022.5359 PMC9084550 35445737

[18] Kofler R, Schlötterer C (2012) Gowinda: unbiased analysis of gene set enrichment for genome-wide association studies. Bioinformatics 28:2084–2085. 10.1093/bioinformatics/bts315 22635606 10.1093/bioinformatics/bts315 PMC3400962

[19] Krusnauskas R, Stakaitis R, Steponaitis G, Almstrup K, Vaitkiene P (2023) Identification and comparison of m6A modifications in glioblastoma non-coding RNAs with MeRIP-seq and nanopore dRNA-seq. Epigenetics 18:2163365. 10.1080/15592294.2022.2163365 36597408 10.1080/15592294.2022.2163365 PMC9980576

[20] Lee CY, Chooi WH, Ng SY, Chew SY (2023) Modulating neuroinflammation through molecular, cellular and biomaterial-based approaches to treat spinal cord injury. Bioeng Transl Med 8:e10389. 10.1002/btm2.10389 36925680 10.1002/btm2.10389 PMC10013833

[21] Leek JT, Johnson WE, Parker HS, Jaffe AE, Storey JD (2012) The Sva package for removing batch effects and other unwanted variation in high-throughput experiments. Bioinformatics 28:882–883. 10.1093/bioinformatics/bts034 22257669 10.1093/bioinformatics/bts034 PMC3307112

[22] Li C, Li B, Wang H, Qu L, Liu H, Weng C, Han J, Li Y (2023) Role of N6-methyladenosine methylation in glioma: recent insights and future directions. Cell Mol Biol Lett 28:103. 10.1186/s11658-023-00514-0 38072944 10.1186/s11658-023-00514-0 PMC10712162

[23] Li C, Liu W, Liu C, Luo Q, Luo K, Wei C, Li X, Qin J, Zheng C, Lan C, Wei S, Tan R, Chen J, Chen Y, Huang H, Zhang G, Wang X (2023) Integrating machine learning and bioinformatics analysis to m6A regulator-mediated methylation modification models for predicting glioblastoma patients’ prognosis and immunotherapy response. Aging 15:4051–4070. 10.18632/aging.204495 37244287 10.18632/aging.204495 PMC10257999

[24] Li J, Huang Y, Cui Q, Zhou Y (2020) m6Acorr: an online tool for the correction and comparison of m. BMC Bioinformatics 21:31. 10.1186/s12859-020-3380-6 31996134 10.1186/s12859-020-3380-6 PMC6988237

[25] Liu J, Huang H, Zhang M, Qing G, Liu H (2023) Intertwined regulation between RNA m. Cell Insight 2:100075. 10.1016/j.cellin.2022.100075 37192910 10.1016/j.cellin.2022.100075 PMC10120304

[26] Lv D, Zhong C, Dixit D, Yang K, Wu Q, Godugu B, Prager BC, Zhao G, Wang X, Xie Q, Bao S, He C, Heiland DH, Rosenfeld MG, Rich JN (2023) EGFR promotes ALKBH5 nuclear retention to attenuate N6-methyladenosine and protect against ferroptosis in glioblastoma. Mol Cell 83:4334–4351e4337. 10.1016/j.molcel.2023.10.025 37979586 10.1016/j.molcel.2023.10.025 PMC10842222

[27] Gagné M, Boulay L, Topisirovic K, Huot I, Mallette M FA (2017) Oncogenic activities of IDH1/2 mutations: from epigenetics to cellular signaling. Trends Cell Biol 27:738–752. 10.1016/j.tcb.2017.06.002 28711227 10.1016/j.tcb.2017.06.002

[28] Mabb AM, Ehlers MD (2010) Ubiquitination in postsynaptic function and plasticity. Annu Rev Cell Dev Biol 26:179–210. 10.1146/annurev-cellbio-100109-104129 20604708 10.1146/annurev-cellbio-100109-104129 PMC3163670

[29] Marallano VJ, Ughetta ME, Tejero R, Nanda S, Ramalingam R, Stalbow L, Sattiraju A, Huang Y, Ramakrishnan A, Shen L, Wojcinski A, Kesari S, Zou H, Tsankov AM, Friedel RH (2024) Hypoxia drives shared and distinct transcriptomic changes in two invasive glioma stem cell lines. Sci Rep 14:7246. 10.1038/s41598-024-56102-5 38538643 10.1038/s41598-024-56102-5 PMC10973515

[30] Mitschka S, Mayr C (2022) Context-specific regulation and function of mRNA alternative polyadenylation. Nat Rev Mol Cell Biol 23:779–796. 10.1038/s41580-022-00507-5 35798852 10.1038/s41580-022-00507-5 PMC9261900

[31] Nishino M, Giobbie-Hurder A, Manos MP, Bailey N, Buchbinder EI, Ott PA, Ramaiya NH, Hodi FS (2017) Immune-Related tumor response dynamics in melanoma patients treated with pembrolizumab: identifying markers for clinical outcome and treatment decisions. Clin Cancer Res 23:4671–4679. 10.1158/1078-0432.CCR-17-0114 28592629 10.1158/1078-0432.CCR-17-0114 PMC5559305

[32] O’Rourke MB, Town SEL, Dalla PV, Bicknell F, Koh Belic N, Violi JP, Steele JR, Padula MP (2019) What is normalization?? The strategies employed in Top-Down and Bottom-Up proteome analysis workflows. Proteomes 7. 10.3390/proteomes7030029 10.3390/proteomes7030029 PMC6789750 31443461

[33] Obara-Michlewska M, Szeliga M (2020) Targeting glutamine addiction in gliomas. Cancers (Basel) 12. 10.3390/cancers12020310 10.3390/cancers12020310 PMC7072559 32013066

[34] Ostrom QT, Price M, Neff C, Cioffi G, Waite KA, Kruchko C, Barnholtz-Sloan JS (2023) CBTRUS statistical report: primary brain and other central nervous system tumors diagnosed in the united States in 2016–2020. Neuro Oncol 25:iv1–iv99. 10.1093/neuonc/noad149 37793125 10.1093/neuonc/noad149 PMC10550277

[35] Palmer JD, Bhamidipati D, Shukla G, Sharma D, Glass J, Kim L, Evans JJ, Judy K, Farrell C, Andrews DW, Wang ZW, Peiper SC, Werner-Wasik M, Shi W (2019) Rapid early tumor progression is prognostic in glioblastoma patients. Am J Clin Oncol 42:481–486. 10.1097/COC.0000000000000537 30973372 10.1097/COC.0000000000000537

[36] Papa S, Choy PM, Bubici C (2019) The ERK and JNK pathways in the regulation of metabolic reprogramming. Oncogene 38:2223–2240. 10.1038/s41388-018-0582-8 30487597 10.1038/s41388-018-0582-8 PMC6398583

[37] Parker MI, Nikonova AS, Sun D, Golemis EA (2020) Proliferative signaling by ERBB proteins and RAF/MEK/ERK effectors in polycystic kidney disease. Cell Signal 67:109497. 10.1016/j.cellsig.2019.109497 31830556 10.1016/j.cellsig.2019.109497 PMC6957738

[38] Pitcher JL, Alexander N, Miranda PJ, Johns TG (2022) ErbB4 in the brain: focus on high grade glioma. Front Oncol 12:983514. 10.3389/fonc.2022.983514 36119496 10.3389/fonc.2022.983514 PMC9471956

[39] Poteet E, Choudhury GR, Winters A, Li W, Ryou MG, Liu R, Tang L, Ghorpade A, Wen Y, Yuan F, Keir ST, Yan H, Bigner DD, Simpkins JW, Yang SH (2013) Reversing the Warburg effect as a treatment for glioblastoma. J Biol Chem 288:9153–9164. 10.1074/jbc.M112.440354 23408428 10.1074/jbc.M112.440354 PMC3610988

[40] Roca CP, Gomes SI, Amorim MJ, Scott-Fordsmand JJ (2017) Variation-preserving normalization unveils blind spots in gene expression profiling. Sci Rep 7:42460. 10.1038/srep42460 28276435 10.1038/srep42460 PMC5343588

[41] Ruta V, Pagliarini V, Sette C (2021) Coordination of RNA processing regulation by signal transduction pathways. Biomolecules 11. 10.3390/biom11101475 10.3390/biom11101475 PMC8533259 34680108

[42] Scholz N, Kurian KM, Siebzehnrubl FA, Licchesi JDF (2020) Targeting the ubiquitin system in glioblastoma. Front Oncol 10:574011. 10.3389/fonc.2020.574011 33324551 10.3389/fonc.2020.574011 PMC7724090

[43] Stupp R, Mason WP, van den Bent MJ, Weller M, Fisher B, Taphoorn MJ, Belanger K, Brandes AA, Marosi C, Bogdahn U, Curschmann J, Janzer RC, Ludwin SK, Gorlia T, Allgeier A, Lacombe D, Cairncross JG, Eisenhauer E, Mirimanoff RO, Groups EOfRaToCBTaR (2005) Radiotherapy plus concomitant and adjuvant Temozolomide for glioblastoma. N Engl J Med 352:987–996. 10.1056/NEJMoa043330. Group NCIoCCT 15758009 10.1056/NEJMoa043330

[44] Sánchez-Baizán N, Ribas L, Piferrer F (2022) Improved biomarker discovery through a plot twist in transcriptomic data analysis. BMC Biol 20:208. 10.1186/s12915-022-01398-w 36153614 10.1186/s12915-022-01398-w PMC9509653

[45] Hastie T, Narasimhan RTB, Chu G (2019) Pamr: Pam: prediction analysis for microarrays. R package version 1.56.1 Edn. CRAN

[46] Tibshirani R, Hastie T, Narasimhan B, Chu G (2002) Diagnosis of multiple cancer types by shrunken centroids of gene expression. Proc Natl Acad Sci U S A 99:6567–6572. 10.1073/pnas.082099299 12011421 10.1073/pnas.082099299 PMC124443

[47] Torrini C, Nguyen TTT, Shu C, Mela A, Humala N, Mahajan A, Seeley EH, Zhang G, Westhoff MA, Karpel-Massler G, Bruce JN, Canoll P, Siegelin MD (2022) Lactate is an epigenetic metabolite that drives survival in model systems of glioblastoma. Mol Cell 82:3061–3076e3066. 10.1016/j.molcel.2022.06.030 35948010 10.1016/j.molcel.2022.06.030 PMC9391294

[48] Wang K, Li M, Hakonarson H (2010) Analysing biological pathways in genome-wide association studies. Nat Rev Genet 11:843–854. 10.1038/nrg2884 21085203 10.1038/nrg2884

[49] Wang W, Kumm ZT, Ho C, Zanesco-Fontes I, Texiera G, Reis RM, Martinetto H, Khan J, McCandless MG, Baker KE, Anderson MD, Chohan MO, Beyer S, Elder JB, Giglio P, Otero JJ (2024) Unsupervised machine learning models reveal predictive clinical markers of glioblastoma patient survival using white blood cell counts prior to initiating chemoradiation. Neurooncol Adv 6:vdad140. 10.1093/noajnl/vdad140 38405202 10.1093/noajnl/vdad140 PMC10894654

[50] Wang W, Tugaoen JD, Fadda P, Toland AE, Ma Q, Elder JB, Giglio P, Otero JJ, Team JCCIN-O (2023) Glioblastoma pseudoprogression and true progression reveal spatially variable transcriptional differences. Acta Neuropathol Commun 11:192. 10.1186/s40478-023-01587-w 38049893 10.1186/s40478-023-01587-w PMC10694987

[51] Waqar M, Roncaroli F, Lehrer EJ, Palmer JD, Villanueva-Meyer J, Braunstein S, Hall E, Aznar M, De Witt Hamer PC, D’Urso PI, Trifiletti D, Quiñones-Hinojosa A, Wesseling P, Borst GR (2022) Rapid early progression (REP) of glioblastoma is an independent negative prognostic factor: results from a systematic review and meta-analysis. Neurooncol Adv 4:vdac075. 10.1093/noajnl/vdac075 35769410 10.1093/noajnl/vdac075 PMC9234755

[52] Welsh H, Batalha CMPF, Li W, Mpye KL, Souza-Pinto NC, Naslavsky MS, Parra EJ (2023) A systematic evaluation of normalization methods and probe replicability using infinium EPIC methylation data. Clin Epigenetics 15:41. 10.1186/s13148-023-01459-z 36906598 10.1186/s13148-023-01459-z PMC10008016

[53] Wen PY, Macdonald DR, Reardon DA, Cloughesy TF, Sorensen AG, Galanis E, Degroot J, Wick W, Gilbert MR, Lassman AB, Tsien C, Mikkelsen T, Wong ET, Chamberlain MC, Stupp R, Lamborn KR, Vogelbaum MA, van den Bent MJ, Chang SM (2010) Updated response assessment criteria for high-grade gliomas: response assessment in neuro-oncology working group. J Clin Oncol 28:1963–1972. 10.1200/JCO.2009.26.3541 20231676 10.1200/JCO.2009.26.3541

[54] Wolf S, Melo D, Garske KM, Pallares LF, Lea AJ, Ayroles JF (2023) Characterizing the landscape of gene expression variance in humans. PLoS Genet 19:e1010833. 10.1371/journal.pgen.1010833 37410774 10.1371/journal.pgen.1010833 PMC10353820

[55] Wu WJ, Xiao F, Xiong Y, Sun GF, Guo Y, Zhou X, Hu GW, Huang K, Guo H (2023) N6-methyladenosine (m6A)-connected LncRNAs are linked to survival and immune infiltration in glioma patients. Biosci Rep 43. 10.1042/BSR20222100 10.1042/BSR20222100 PMC10170299 37083719

[56] Yadav P, Subbarayalu P, Medina D, Nirzhor S, Timilsina S, Rajamanickam S, Eedunuri VK, Gupta Y, Zheng S, Abdelfattah N, Huang Y, Vadlamudi R, Hromas R, Meltzer P, Houghton P, Chen Y, Rao MK (2022) M6A RNA methylation regulates histone ubiquitination to support Cancer growth and progression. Cancer Res 82:1872–1889. 10.1158/0008-5472.CAN-21-2106 35303054 10.1158/0008-5472.CAN-21-2106 PMC9336196

[57] Yosef A, Shnaider E, Schneider M, Gurevich M (2023) Normalization of Large-Scale transcriptome data using heuristic methods. Bioinform Biol Insights 17:11779322231160397. 10.1177/11779322231160397 37020503 10.1177/11779322231160397 PMC10068970

[58] Young JS, Al-Adli N, Scotford K, Cha S, Berger MS (2023) Pseudoprogression versus true progression in glioblastoma: what neurosurgeons need to know. J Neurosurg 139:748–759. 10.3171/2022.12.JNS222173 36790010 10.3171/2022.12.JNS222173 PMC10412732

[59] Yuan F, Hankey W, Wagner EJ, Li W, Wang Q (2021) Alternative polyadenylation of mRNA and its role in cancer. Genes Dis 8:61–72. 10.1016/j.gendis.2019.10.011 33569514 10.1016/j.gendis.2019.10.011 PMC7859462

[60] Zhang B, Horvath S (2005) A general framework for weighted gene co-expression network analysis. Stat Appl Genet Mol Biol 4:Article17. 10.2202/1544-6115.1128 16646834 10.2202/1544-6115.1128

[61] Zhang Z, Chen LQ, Zhao YL, Yang CG, Roundtree IA, Ren J, Xie W, He C, Luo GZ (2019) Single-base mapping of m. Sci Adv 5:eaax0250. 10.1126/sciadv.aax0250 31281898 10.1126/sciadv.aax0250 PMC6609220

[62] Zhao Y, Wong L, Goh WWB (2020) How to do quantile normalization correctly for gene expression data analyses. Sci Rep 10:15534. 10.1038/s41598-020-72664-6 32968196 10.1038/s41598-020-72664-6 PMC7511327

[63] Zheng X, Li S, Yu J, Dai C, Yan S, Chen G, Sun C (2023) N Transl Cancer Res 12:992–1005. 10.21037/tcr-23-449 37180667 10.21037/tcr-23-449 PMC10174994

