# Supplementary material for: Epitranscriptomic analysis reveals clinical and molecular signatures in glioblastoma
Source: Acta Neuropathol Commun. 2025 Apr 11;13:74. doi: 10.1186/s40478-025-01966-5 (PMC11987271; doi:10.1186/s40478-025-01966-5)
Supplement: Supplementary file 1 — Supplementary Material 1 [file 40478_2025_1966_MOESM1_ESM.docx]

**Table S1: Clinicopathological Comparison between Progression and Pseudoprogression in Glioblastoma Patients**

|  | **PD**  **n=33(36%)** | | | **psPD**  **n=59(64%)** | | | **p-value** | | **Effect Size** | |  |
| --- | --- | --- | --- | --- | --- | --- | --- | --- | --- | --- | --- |
| **Gender** ^a^ |  |  |  | |  |  | |  | | 0.00 ^1^ | |
| Female | 21 | (35.6) | 12 | | (36.4) | 1.00 | |  | |  | |
| Male | 38 | (64.4) | 21 | | (63.6) |  | |  | |  | |
| **Age (years)** ^b^ | 63 | (15.5) | 67 | | (11.0) | 0.85 | |  | | 0.02 ^1^ | |
| **Time Survival** ^b^ | 21 | (18.0) | 20 | | (19.0) | 0.74 | |  | | 0.06 ^1^ | |
| **BMI** ^b^ | 29.1 | (8.1) | 28.3 | | (9.8) | 0.73 | |  | | 0.03 ^1^ | |
| **Lesion Size (cm)** ^b^ | 4.1 | (1.5) | 4.0 | | (1.5) | 0.08 | |  | | 0.05 ^1^ | |
| **Side** ^c^ |  |  |  | |  |  | |  | |  | |
| Left | 33 | (55.9) | 16 | | (58.5) | 0.64 | |  | | 0.09 ^1^ | |
| Right | 26 | (44.1) | 17 | | (51.5) |  | |  | |  | |
| **Lobe Location** ^a^ |  |  |  | |  |  | |  | | 0.68 ^2^ | |
| Frontal | 19 | (32.2) | 10 | | (30.3) |  | |  | |  | |
| Occipital | **0** | **(0.0)** | 5 | | (15.2) | 0.03 | | * | |  | |
| Parietal | 17 | (28.8) | 7 | | (21.2) |  | |  | |  | |
| Temporal | 23 | (39.0) | 11 | | (33.3) |  | |  | |  | |
| **Midline Shift** ^a^ |  |  |  | |  |  | |  | | 0.41 ^2^ | |
| Yes | 33 | (28.2) | 26 | | (7.6) |  | |  | |  | |
| No | 26 | (35.9) | 7 | | (28.2) |  | |  | |  | |
| **Ki-67 Positive Percent** ^b^ | 30 | (20.0) | 23 | | (15.5) | 0.04 | | * | | 0.22 ^2^ | |
| **MGMT Status** ^a^ |  |  |  | |  |  | |  | | 0.35 ^2^ | |
| Hypermethylated/ Methylated | 22 | (37.3) | 19 | | (57.6) | 0.10 | |  | |  | |
| Not Hypermethylated/ Unmethylated | 37 | (62.7) | 14 | | (42.4) |  | |  | |  | |
| **EGFR Amplification** ^a^ |  |  |  | |  |  | |  | | 0.45 ^2^ | |
| No | 27 | (62.8) | 21 | | (70.0) | 0.10 | |  | |  | |
| Yes | 16 | (37.2) | 9 | | (30.0) |  | |  | |  | |
| **P53 Mutation Status (>10% of Cells)** ^a^ |  |  |  | |  |  | |  | | 0.18 ^1^ | |
| Negative | 10 | (18.5) | 8 | | (26.7) | 0.68 | |  | |  | |
| Positive | 44 | (82.5) | 22 | | (73.3) |  | |  | |  | |
| **WBC (Pre-surgery)** ^b^ | 9.8 | (6.3) | 10.5 | | (6.3) | 0.88 | |  | | 0.01 ^1^ | |
| **Hemoglobin (Pre-surgery)** ^c^ | 14.1 | (1.7) | 14.6 | | (2.7) | 0.63 | |  | | 0.05 ^1^ | |
| **Platelets (Pre-surgery)** ^c^ | 235.6 | (61.8) | 254.0 | | (77.2) | 0.25 | |  | | 0.26 ^2^ | |
| **Neutrophils (Segs + Bands) (Pre-surgery)** ^c^ | 8.7 | (4.3) | 8.6 | | (4.4) | 0.93 | |  | | 0.01 ^1^ | |
| **Lymphocytes (Pre-surgery)** ^c^ | 1.2 | (0.7) | 1.3 | | (1.0) | 0.42 | |  | | 0.15 ^1^ | |
| **Number of Gy administered** ^a^ |  |  |  | |  |  | |  | | 0.41^2^ | |
| 40 | 5 | (8.5) | 4 | | (12.1) | 0.84 | |  | |  | |
| 60 | 54 | (91.5) | 29 | | (87.9) |  | |  | |  | |
| **Number of Gy Fractions** ^a^ |  |  |  | |  |  | |  | | 0.04 ^1^ | |
| 15 | 5 | (8.5) | 4 | | (12.1) | 0.84 | |  | |  | |
| 30 | 54 | (91.5) | 29 | | (87.9) |  | |  | |  | |
| **Cycles Completed** ^b^ | 5 | (4.0) | 6 | | (4.0) | 0.85 | |  | | 0.02 ^1^ | |
| **Adjuvant Temodar Dose** ^b^ | 340 | (165.0) | 320 | | (120.0) | 0.15 | |  | | 0.09 ^1^ | |

^a^ n (%), ^b^ median (IQR = interquartile range), ^c^ mean (SD = standard deviation)

^1^ small (≤ 0.2), ^2^ moderate (= 0.5), ^3^ large (≥ 0.8)

**BMI** = Body Mass Index, **WBC** = White Blood Cell

*p-value significant (p<0.05) tested using Fisher exact test for Lobe location variable, chi-square for categorical variables, and t-test or Wilcoxon for continuous variables.

**Table S2**: Enriched GO Processes and KEGG Pathways Associated with Clinical and Histopathological Significant in Glioblastoma

| **Term** | **p-value** | **q-value** | **z-score** | **combined score** | **Chi-square comparison p-value** |
| --- | --- | --- | --- | --- | --- |
| **All variance between methylated transcripts** |  |  |  |  |  |
| regulation of transcription by RNA polymerase II (GO:0006357) | 4.51E-29 | 2.38E-25 | 1.844 | 120.4 | 3.51E-50 |
| regulation of transcription, DNA-templated (GO:0006355) | 2.44E-28 | 6.43E-25 | 1.823 | 115.9 | 1.56E-51 |
| positive regulation of transcription, DNA-templated (GO:0045893) | 1.16E-26 | 2.05E-23 | 2.118 | 126.5 | 2.45E-06 |
| negative regulation of transcription, DNA-templated (GO:0045892) | 8.41E-22 | 1.11E-18 | 2.107 | 102.2 | 0.205298817 |
| positive regulation of transcription by RNA polymerase II (GO:0045944) | 1.80E-20 | 1.90E-17 | 2.087 | 94.86 | 0.56830193 |
| negative regulation of transcription by RNA polymerase II (GO:0000122) | 1.28E-15 | 9.67E-13 | 2.063 | 70.72 | 0.002857103 |
| transcription by RNA polymerase II (GO:0006366) | 2.11E-12 | 1.01E-09 | 2.443 | 65.67 | 3.66E-18 |
| regulation of gene expression (GO:0010468) | 3.99E-10 | 1.75E-07 | 1.605 | 34.74 | 0.682855322 |
| transcription, DNA-templated (GO:0006351) | 3.38E-09 | 1.37E-06 | 2.453 | 47.86 | 2.75E-27 |
| protein ubiquitination (GO:0016567) | 1.59E-08 | 5.98E-06 | 1.801 | 32.34 | 6.84E-11 |
| mitotic cell cycle phase transition (GO:0044772) | 2.27E-08 | 7.62E-06 | 2.396 | 42.17 | 6.23E-29 |
| ubiquitin-dependent protein catabolic process (GO:0006511) | 2.88E-08 | 8.44E-06 | 1.99 | 34.55 | 3.55E-19 |
| protein phosphorylation (GO:0006468) | 8.66E-08 | 2.40E-05 | 1.774 | 28.84 | 1.36E-12 |
| phosphorylation (GO:0016310) | 1.16E-07 | 3.06E-05 | 1.867 | 29.81 | 3.48E-17 |
| regulation of Rho protein signal transduction (GO:0035023) | 2.47E-07 | 6.20E-05 | 3.648 | 55.51 | 3.69E-43 |
| chromatin remodeling (GO:0006338) | 2.95E-07 | 7.07E-05 | 3.015 | 45.34 | 9.22E-40 |
| positive regulation of mRNA catabolic process (GO:0061014) | 4.50E-07 | 0.0001031 | 4.936 | 72.13 | 3.29E-47 |
| cellular response to DNA damage stimulus (GO:0006974) | 4.70E-07 | 0.0001032 | 1.881 | 27.41 | 1.71E-20 |
| positive regulation of cellular response to transforming growth factor beta stimulus (GO:1903846) | 7.26E-07 | 0.0001367 | 8.216 | 116.1 | 1.57E-50 |
| positive regulation of transforming growth factor beta receptor signaling pathway (GO:0030511) | 7.26E-07 | 0.0001367 | 8.216 | 116.1 | 1.57E-50 |
|  |  |  |  |  |  |
| **Term** | **p-value** | **q-value** | **z-score** | **combined score** | **Chi-square comparasion p-value** |
| **30% lower variance between methylated transcripts** |  |  |  |  |  |
| negative regulation of transcription, DNA-templated (GO:0045892) | 9.29E-14 | 1.47E-10 | 2.246 | 67.38 | 2.14E-73 |
| regulation of transcription by RNA polymerase II (GO:0006357) | 1.02E-13 | 1.47E-10 | 1.804 | 53.97 | 2.80E-218 |
| regulation of transcription, DNA-templated (GO:0006355) | 1.09E-13 | 1.47E-10 | 1.797 | 53.62 | 9.45E-223 |
| positive regulation of transcription, DNA-templated (GO:0045893) | 5.55E-12 | 5.59E-09 | 2.003 | 51.9 | 6.69E-92 |
| positive regulation of transcription by RNA polymerase II (GO:0045944) | 1.31E-10 | 1.06E-07 | 2.061 | 46.9 | 2.38E-59 |
| negative regulation of transcription by RNA polymerase II (GO:0000122) | 1.62E-10 | 1.09E-07 | 2.235 | 50.38 | 6.92E-39 |
| regulation of small GTPase mediated signal transduction (GO:0051056) | 1.91E-06 | 0.0007003 | 3.212 | 42.29 | 0.194729654 |
| phosphorylation (GO:0016310) | 2.83E-06 | 0.0009512 | 2.139 | 27.32 | 1.60E-10 |
| transcription by RNA polymerase II (GO:0006366) | 3.80E-06 | 0.001179 | 2.276 | 28.41 | 9.92E-07 |
| regulation of Rho protein signal transduction (GO:0035023) | 4.49E-06 | 0.001293 | 4.297 | 52.9 | 0.699435114 |
| regulation of transforming growth factor beta receptor signaling pathway (GO:0017015) | 8.10E-06 | 0.001921 | 3.542 | 41.52 | 0.98172392 |
| protein-containing complex assembly (GO:0065003) | 7.65E-06 | 0.001921 | 2.365 | 27.86 | 0.000191989 |
| regulation of apoptotic process (GO:0042981) | 7.33E-06 | 0.001921 | 1.764 | 20.86 | 2.83E-30 |
| protein phosphorylation (GO:0006468) | 1.06E-05 | 0.002363 | 1.934 | 22.16 | 1.75E-14 |
| histone H3-K4 methylation (GO:0051568) | 1.46E-05 | 0.002932 | 7.053 | 78.55 | 0.051519585 |
| regulation of I-kappaB kinase/NF-kappaB signaling (GO:0043122) | 1.44E-05 | 0.002932 | 2.458 | 27.39 | 0.006267702 |
| Ras protein signal transduction (GO:0007265) | 2.17E-05 | 0.00416 | 2.884 | 30.98 | 0.380698141 |
| positive regulation of intracellular signal transduction (GO:1902533) | 2.68E-05 | 0.004906 | 1.834 | 19.31 | 2.38E-16 |
| regulation of gene expression (GO:0010468) | 3.23E-05 | 0.00565 | 1.572 | 16.26 | 3.33E-53 |
| regulation of cell cycle (GO:0051726) | 3.54E-05 | 0.005705 | 2.161 | 22.15 | 8.65E-05 |
|  |  |  |  |  |  |
| **Term** | **p-value** | **q-value** | **z-score** | **combined score** | **Chi-square comparasion p-value** |
| **30% higher variance between methylated transcripts** |  |  |  |  |  |
| regulation of transcription by RNA polymerase II (GO:0006357) | 1.89E-13 | 7.46E-10 | 1.766 | 51.73 | 2.05E-64 |
| regulation of transcription, DNA-templated (GO:0006355) | 1.84E-11 | 3.62E-08 | 1.681 | 41.56 | 4.33E-61 |
| positive regulation of transcription, DNA-templated (GO:0045893) | 2.45E-09 | 3.23E-06 | 1.812 | 35.93 | 3.30E-11 |
| transcription by RNA polymerase II (GO:0006366) | 7.25E-09 | 5.72E-06 | 2.645 | 49.57 | 0.011153819 |
| negative regulation of transcription, DNA-templated (GO:0045892) | 5.85E-09 | 5.72E-06 | 1.887 | 35.77 | 2.18E-05 |
| protein ubiquitination (GO:0016567) | 2.88E-07 | 0.0001897 | 2.054 | 30.93 | 0.537086711 |
| regulation of telomere maintenance (GO:0032204) | 6.66E-07 | 0.0003758 | 9.611 | 136.7 | 7.02E-14 |
| transcription, DNA-templated (GO:0006351) | 2.70E-06 | 0.001146 | 2.572 | 32.99 | 6.64E-06 |
| negative regulation of transcription by RNA polymerase II (GO:0000122) | 2.40E-06 | 0.001146 | 1.82 | 23.55 | 0.556288614 |
| positive regulation of transcription by RNA polymerase II (GO:0045944) | 2.90E-06 | 0.001146 | 1.697 | 21.63 | 0.005798296 |
| regulation of microtubule-based process (GO:0032886) | 2.67E-05 | 0.008768 | 5.764 | 60.7 | 7.02E-14 |
| chromatin remodeling (GO:0006338) | 3.47E-05 | 0.01055 | 3.167 | 32.51 | 1.54E-10 |
| modification-dependent protein catabolic process (GO:0019941) | 3.89E-05 | 0.01098 | 2.404 | 24.41 | 3.53E-07 |
| chromatin organization (GO:0006325) | 6.52E-05 | 0.01429 | 2.675 | 25.79 | 3.17E-09 |
| ubiquitin-dependent protein catabolic process (GO:0006511) | 5.79E-05 | 0.01429 | 1.975 | 19.27 | 0.000831727 |
| regulation of chromosome organization (GO:0033044) | 8.51E-05 | 0.01753 | 7.474 | 70.04 | 4.06E-15 |
| mitotic sister chromatid segregation (GO:0000070) | 9.73E-05 | 0.01753 | 3.006 | 27.77 | 6.99E-11 |
| proteasome-mediated ubiquitin-dependent protein catabolic process (GO:0043161) | 0.0001022 | 0.01753 | 1.988 | 18.27 | 0.000141722 |
| cellular response to DNA damage stimulus (GO:0006974) | 8.93E-05 | 0.01753 | 1.95 | 18.18 | 0.000545113 |
| protein polyubiquitination (GO:0000209) | 0.0001285 | 0.02114 | 1.983 | 17.77 | 8.81E-05 |
|  |  |  |  |  |  |
| **Term** | **p-value** | **q-value** | **z-score** | **combined score** | **Chi-square comparasion p-value** |
| **Differential Methylated transcripts** |  |  |  |  |  |
| histone monoubiquitination (GO:0010390) | 0.0001357 | 0.04846 | 35.09 | 312.5 | 1.73E-17 |
| protein modification by small protein conjugation (GO:0032446) | 0.0003209 | 0.05768 | 5.123 | 41.21 | 1.18E-13 |
| protein ubiquitination (GO:0016567) | 0.0003545 | 0.05768 | 4.512 | 35.84 | 5.97E-13 |
| negative regulation of mRNA polyadenylation (GO:1900364) | 0.0004801 | 0.05768 | 80.93 | 618.5 | 2.58E-18 |
| regulation of collateral sprouting (GO:0048670) | 0.0004801 | 0.05768 | 80.93 | 618.5 | 2.58E-18 |
| negative regulation of mRNA 3'-end processing (GO:0031441) | 0.0006156 | 0.06454 | 69.37 | 512.8 | 2.58E-18 |
| negative regulation of transcription from RNA polymerase II promoter in response to stress (GO:0097201) | 0.0007674 | 0.06454 | 60.7 | 435.3 | 2.58E-18 |
| histone H2B ubiquitination (GO:0033523) | 0.0007674 | 0.06454 | 60.7 | 435.3 | 2.58E-18 |
| ncRNA processing (GO:0034470) | 0.001584 | 0.111 | 6.368 | 41.06 | 6.80E-16 |
| mRNA methylation (GO:0080009) | 0.001766 | 0.1143 | 37.34 | 236.7 | 2.58E-18 |
| RNA methylation (GO:0001510) | 0.002058 | 0.1236 | 12.9 | 79.82 | 1.73E-17 |
| regulation of mitochondrial membrane permeability (GO:0046902) | 0.002845 | 0.1408 | 28.55 | 167.4 | 2.58E-18 |
| regulation of histone H3-K4 methylation (GO:0051569) | 0.002845 | 0.1408 | 28.55 | 167.4 | 2.58E-18 |
| cell junction organization (GO:0034330) | 0.00346 | 0.1538 | 10.65 | 60.37 | 1.73E-17 |
| histone ubiquitination (GO:0016574) | 0.003475 | 0.1538 | 25.54 | 144.6 | 2.58E-18 |
| mRNA modification (GO:0016556) | 0.003813 | 0.1603 | 24.26 | 135.1 | 2.58E-18 |
|  |  |  |  |  |  |
| **Term** | **p-value** | **q-value** | **z-score** | **combined score** | **Chi-square comparasion p-value** |
| **intersection of Differential Methylated transcripts Differential Expressed Genes** | |  |  |  |  |
| regulation of histone H3-K4 methylation (GO:0051569) | 7.73E-05 | 0.008154 | 195.8 | 1854 | 0.012554919 |
| histone monoubiquitination (GO:0010390) | 0.0001245 | 0.008154 | 151.2 | 1360 | 0.012554919 |
| mitochondrial transport (GO:0006839) | 0.0006857 | 0.02994 | 61.52 | 448.2 | 0.012554919 |
| cell fate commitment involved in formation of primary germ layer (GO:0060795) | 0.003495 | 0.0513 | 384.3 | 2174 | 0.002281937 |
| negative regulation of cellular respiration (GO:1901856) | 0.003495 | 0.0513 | 384.3 | 2174 | 0.002281937 |
| regulation of tooth mineralization (GO:0070170) | 0.004193 | 0.0513 | 307.4 | 1683 | 0.002281937 |
| positive regulation of autophagy of mitochondrion in response to mitochondrial depolarization (GO:1904925) | 0.004193 | 0.0513 | 307.4 | 1683 | 0.002281937 |
| negative regulation of transporter activity (GO:0032410) | 0.004193 | 0.0513 | 307.4 | 1683 | 0.002281937 |

**GO**: Gene Ontology

**Table S3:** Overview of Correlation and Linear Model Analysis of m6A Methylation Sites with Genes in Glioblastoma Patients.

| **N** | **Gene** | **Number of m6A sites** | **Corr: Max** | **Corr: p value: Max** | **Corr: Min** | **Corr: p value: Min** |
| --- | --- | --- | --- | --- | --- | --- |
| 1 | A4GALT | 4 | 0.1607 | 0.8381 | -0.0822 | 0.1260 |
| 2 | AARS | 2 | 0.0773 | 0.4639 | 0.0773 | 0.4639 |
| 3 | ABCC1 | 3 | 0.3361 | 0.9664 | -0.0045 | 0.0011 |
| 4 | ABCF1 | 2 | -0.2576 | 0.0132 | -0.2576 | 0.0132 |
| 5 | ABCF3 | 3 | 0.5334 | 0.4696 | -0.0763 | 0.0000 |
| 6 | ABHD13 | 2 | -0.2002 | 0.0557 | -0.2002 | 0.0557 |
| 7 | ABHD14A | 2 | 0.5004 | 0.0000 | 0.5004 | 0.0000 |
| 8 | ABHD15 | 3 | 0.4173 | 0.4750 | 0.0754 | 0.0000 |
| 9 | ABL1 | 6 | 0.4234 | 0.9467 | -0.2131 | 0.0000 |
| 10 | ABL2 | 2 | 0.3066 | 0.0030 | 0.3066 | 0.0030 |
| 11 | ABR | 2 | -0.1346 | 0.2010 | -0.1346 | 0.2010 |
| 12 | ABT1 | 2 | 0.0108 | 0.9189 | 0.0108 | 0.9189 |
| 13 | ACIN1 | 2 | 0.1699 | 0.1053 | 0.1699 | 0.1053 |
| 14 | ACP2 | 3 | 0.2345 | 0.1214 | 0.1626 | 0.0245 |
| 15 | ACSF3 | 2 | -0.0801 | 0.4476 | -0.0801 | 0.4476 |
| 16 | ACTG1 | 3 | 0.0282 | 0.7895 | -0.0715 | 0.4985 |
| 17 | ADAM23 | 2 | 0.1763 | 0.0928 | 0.1763 | 0.0928 |
| 18 | ADAR | 5 | 0.1584 | 0.8925 | -0.1136 | 0.1315 |
| 19 | ADCY3 | 2 | -0.0219 | 0.8360 | -0.0219 | 0.8360 |
| 20 | ADCY9 | 4 | 0.2387 | 0.8994 | -0.1224 | 0.0219 |
| 21 | ADGRL2 | 2 | 0.2565 | 0.0136 | 0.2565 | 0.0136 |
| 22 | ADNP2 | 3 | 0.3912 | 0.0983 | 0.1734 | 0.0001 |
| 23 | ADO | 2 | 0.2448 | 0.0187 | 0.2448 | 0.0187 |
| 24 | ADPRHL2 | 2 | 0.6088 | 0.0000 | 0.6088 | 0.0000 |
| 25 | AES | 2 | -0.2721 | 0.0087 | -0.2721 | 0.0087 |
| 26 | AFF1 | 6 | 0.2516 | 0.9527 | -0.2138 | 0.0156 |
| 27 | AFF4 | 4 | 0.2141 | 0.9361 | -0.0159 | 0.0404 |
| 28 | AFG3L2 | 2 | 0.2413 | 0.0205 | 0.2413 | 0.0205 |
| 29 | AGAP1 | 2 | -0.3645 | 0.0004 | -0.3645 | 0.0004 |
| 30 | AGAP2 | 2 | -0.1565 | 0.1364 | -0.1565 | 0.1364 |
| 31 | AGAP2.AS1 | 2 | -0.1356 | 0.1975 | -0.1356 | 0.1975 |
| 32 | AGO2 | 2 | 0.5518 | 0.0000 | 0.5518 | 0.0000 |
| 33 | AHDC1 | 7 | 0.3226 | 0.9722 | -0.5363 | 0.0000 |
| 34 | AHNAK | 10 | 0.7932 | 0.9458 | -0.1420 | 0.0000 |
| 35 | AHNAK2 | 2 | -0.1214 | 0.2492 | -0.1214 | 0.2492 |
| 36 | AHRR | 3 | 0.1439 | 0.9841 | -0.0488 | 0.1713 |
| 37 | AKAP1 | 6 | 0.6129 | 0.4613 | 0.0778 | 0.0000 |
| 38 | AKAP13 | 3 | 0.5927 | 0.8101 | 0.0254 | 0.0000 |
| 39 | ALDH1B1 | 3 | 0.4240 | 0.5366 | -0.2725 | 0.0000 |
| 40 | ALKBH4 | 2 | 0.0479 | 0.6500 | 0.0479 | 0.6500 |
| 41 | ALMS1 | 2 | -0.2176 | 0.0372 | -0.2176 | 0.0372 |
| 42 | AMBRA1 | 6 | 0.3302 | 0.9421 | -0.4078 | 0.0001 |
| 43 | AMIGO3 | 3 | 0.1360 | 0.3124 | -0.1065 | 0.1962 |
| 44 | AMMECR1L | 2 | -0.1386 | 0.1877 | -0.1386 | 0.1877 |
| 45 | AMOTL1 | 3 | -0.1154 | 0.2735 | -0.1719 | 0.1013 |
| 46 | AMOTL2 | 5 | 0.1298 | 0.8337 | -0.3925 | 0.0001 |
| 47 | ANAPC7 | 2 | 0.2274 | 0.0293 | 0.2274 | 0.0293 |
| 48 | ANKRD10 | 2 | -0.1055 | 0.3168 | -0.1055 | 0.3168 |
| 49 | ANKRD11 | 10 | 0.4601 | 0.9661 | -0.3627 | 0.0000 |
| 50 | ANKRD17 | 4 | 0.2282 | 0.8950 | -0.2427 | 0.0198 |
| 51 | ANKRD40 | 2 | 0.1679 | 0.1097 | 0.1679 | 0.1097 |
| 52 | ANKRD50 | 2 | 0.0720 | 0.4955 | 0.0720 | 0.4955 |
| 53 | ANKS6 | 2 | 0.0842 | 0.4251 | 0.0842 | 0.4251 |
| 54 | ANTXR1 | 2 | -0.1700 | 0.1051 | -0.1700 | 0.1051 |
| 55 | AP1G1 | 3 | -0.0423 | 0.6888 | -0.1812 | 0.0840 |
| 56 | AP3D1 | 2 | 0.2491 | 0.0166 | 0.2491 | 0.0166 |
| 57 | AP5B1 | 2 | 0.0794 | 0.4517 | 0.0794 | 0.4517 |
| 58 | APC | 3 | 0.1350 | 0.7946 | -0.0545 | 0.1993 |
| 59 | APEX1 | 2 | 0.1787 | 0.0883 | 0.1787 | 0.0883 |
| 60 | APLP2 | 3 | 0.2379 | 0.5557 | -0.0622 | 0.0224 |
| 61 | APPL2 | 2 | -0.0447 | 0.6721 | -0.0447 | 0.6721 |
| 62 | AREL1 | 2 | -0.0794 | 0.4519 | -0.0794 | 0.4519 |
| 63 | ARF6 | 2 | -0.2380 | 0.0223 | -0.2380 | 0.0223 |
| 64 | ARFGAP1 | 3 | 0.1729 | 0.6360 | 0.0500 | 0.0993 |
| 65 | ARFIP2 | 2 | 0.0914 | 0.3863 | 0.0914 | 0.3863 |
| 66 | ARHGAP17 | 4 | 0.0573 | 0.8547 | -0.0651 | 0.5377 |
| 67 | ARHGAP32 | 3 | -0.1026 | 0.3305 | -0.2168 | 0.0379 |
| 68 | ARHGAP35 | 8 | 0.4765 | 0.9916 | -0.3807 | 0.0000 |
| 69 | ARHGAP45 | 2 | 0.1380 | 0.1897 | 0.1380 | 0.1897 |
| 70 | ARHGAP5 | 3 | 0.2016 | 0.0540 | -0.5338 | 0.0000 |
| 71 | ARHGDIA | 2 | 0.4215 | 0.0000 | 0.4215 | 0.0000 |
| 72 | ARHGEF11 | 2 | 0.3806 | 0.0002 | 0.3806 | 0.0002 |
| 73 | ARHGEF17 | 6 | 0.3531 | 0.9115 | -0.3060 | 0.0006 |
| 74 | ARHGEF2 | 3 | 0.1932 | 0.5205 | -0.1132 | 0.0650 |
| 75 | ARHGEF40 | 2 | -0.3727 | 0.0003 | -0.3727 | 0.0003 |
| 76 | ARID1A | 4 | 0.2500 | 0.4243 | -0.1543 | 0.0163 |
| 77 | ARID1B | 3 | 0.2095 | 0.4713 | -0.1602 | 0.0450 |
| 78 | ARID5B | 2 | 0.0546 | 0.6054 | 0.0546 | 0.6054 |
| 79 | ARPC5 | 2 | -0.1862 | 0.0755 | -0.1862 | 0.0755 |
| 80 | ARSJ | 2 | -0.2735 | 0.0083 | -0.2735 | 0.0083 |
| 81 | ASB13 | 3 | 0.1236 | 0.7970 | -0.1601 | 0.1274 |
| 82 | ASB6 | 2 | 0.2433 | 0.0195 | 0.2433 | 0.0195 |
| 83 | ASB8 | 3 | 0.1511 | 0.3915 | -0.3930 | 0.0001 |
| 84 | ASCC3 | 2 | -0.0976 | 0.3549 | -0.0976 | 0.3549 |
| 85 | ASH1L | 4 | 0.2450 | 0.9568 | -0.0057 | 0.0186 |
| 86 | ASXL1 | 6 | 0.2860 | 0.9061 | -0.2618 | 0.0057 |
| 87 | ASXL2 | 3 | 0.2968 | 0.8547 | -0.0194 | 0.0041 |
| 88 | ATF7 | 2 | -0.2931 | 0.0046 | -0.2931 | 0.0046 |
| 89 | ATG2A | 2 | 0.1248 | 0.2360 | 0.1248 | 0.2360 |
| 90 | ATG3 | 2 | -0.1590 | 0.1300 | -0.1590 | 0.1300 |
| 91 | ATMIN | 4 | 0.7165 | 0.3733 | -0.3337 | 0.0000 |
| 92 | ATN1 | 2 | 0.5052 | 0.0000 | 0.5052 | 0.0000 |
| 93 | ATP1A1 | 3 | 0.5205 | 0.4314 | -0.1143 | 0.0000 |
| 94 | ATP5MF | 3 | 0.3755 | 0.4219 | 0.0847 | 0.0002 |
| 95 | ATRN | 2 | 0.6412 | 0.0000 | 0.6412 | 0.0000 |
| 96 | ATXN1L | 3 | 0.1387 | 0.6910 | -0.1548 | 0.1407 |
| 97 | AUNIP | 2 | 0.0143 | 0.8925 | 0.0143 | 0.8925 |
| 98 | AUTS2 | 2 | -0.0630 | 0.5508 | -0.0630 | 0.5508 |
| 99 | AVPI1 | 2 | 0.0447 | 0.6725 | 0.0447 | 0.6725 |
| 100 | AZI2 | 2 | 0.0126 | 0.9048 | 0.0126 | 0.9048 |
| 101 | B3GALT6 | 3 | 0.3828 | 0.4333 | -0.3210 | 0.0002 |
| 102 | B3GNT10 | 2 | -0.0419 | 0.6919 | -0.0419 | 0.6919 |
| 103 | BACH2 | 2 | 0.1494 | 0.1553 | 0.1494 | 0.1553 |
| 104 | BAG3 | 2 | -0.1165 | 0.2686 | -0.1165 | 0.2686 |
| 105 | BAZ1B | 4 | 0.0683 | 0.8822 | -0.1320 | 0.2099 |
| 106 | BCAR1 | 4 | 0.2441 | 0.7134 | -0.2051 | 0.0190 |
| 107 | BCAR3 | 2 | -0.1401 | 0.1830 | -0.1401 | 0.1830 |
| 108 | BCL2L1 | 2 | -0.1231 | 0.2422 | -0.1231 | 0.2422 |
| 109 | BCL7A | 5 | 0.2374 | 0.9912 | -0.2426 | 0.0198 |
| 110 | BCL9 | 3 | 0.1493 | 0.4584 | -0.3262 | 0.0015 |
| 111 | BCL9L | 5 | 0.4891 | 0.9015 | -0.1260 | 0.0000 |
| 112 | BCOR | 3 | 0.1919 | 0.8869 | -0.2053 | 0.0496 |
| 113 | BCR | 3 | 0.4618 | 0.0329 | 0.2227 | 0.0000 |
| 114 | BDKRB2 | 2 | 0.0709 | 0.5018 | 0.0709 | 0.5018 |
| 115 | BET1L | 2 | 0.0741 | 0.4827 | 0.0741 | 0.4827 |
| 116 | BIN3 | 2 | 0.1724 | 0.1003 | 0.1724 | 0.1003 |
| 117 | BIRC2 | 2 | -0.0810 | 0.4428 | -0.0810 | 0.4428 |
| 118 | BMP2K | 2 | 0.0407 | 0.7001 | 0.0407 | 0.7001 |
| 119 | BOD1L1 | 4 | 0.1970 | 0.9429 | -0.0588 | 0.0598 |
| 120 | BOK | 5 | 0.3985 | 0.8501 | -0.3842 | 0.0001 |
| 121 | BOLA1 | 2 | 0.5485 | 0.0000 | 0.5485 | 0.0000 |
| 122 | BORCS6 | 2 | 0.1607 | 0.1258 | 0.1607 | 0.1258 |
| 123 | BPTF | 3 | 0.2572 | 0.2787 | -0.1141 | 0.0133 |
| 124 | BRAT1 | 2 | 0.2990 | 0.0038 | 0.2990 | 0.0038 |
| 125 | BRD1 | 2 | -0.1962 | 0.0608 | -0.1962 | 0.0608 |
| 126 | BRD2 | 4 | 0.5029 | 0.7616 | -0.1875 | 0.0000 |
| 127 | BRD4 | 3 | 0.3631 | 0.2494 | -0.1810 | 0.0004 |
| 128 | BRD8 | 3 | 0.0082 | 0.9382 | -0.5806 | 0.0000 |
| 129 | BRI3BP | 3 | 0.6121 | 0.2059 | -0.1562 | 0.0000 |
| 130 | BTBD6 | 3 | 0.2095 | 0.7778 | -0.0298 | 0.0451 |
| 131 | BTBD7 | 2 | 0.3365 | 0.0010 | 0.3365 | 0.0010 |
| 132 | BTN3A2 | 2 | 0.0907 | 0.3901 | 0.0907 | 0.3901 |
| 133 | BUB3 | 2 | -0.0395 | 0.7083 | -0.0395 | 0.7083 |
| 134 | BUD13 | 2 | 0.2811 | 0.0066 | 0.2811 | 0.0066 |
| 135 | BYSL | 5 | 0.3879 | 0.8649 | -0.2168 | 0.0001 |
| 136 | C12orf43 | 3 | 0.1063 | 0.7800 | -0.0878 | 0.3131 |
| 137 | C15orf39 | 5 | 0.5017 | 0.5707 | -0.0599 | 0.0000 |
| 138 | C17orf80 | 2 | 0.0595 | 0.5734 | 0.0595 | 0.5734 |
| 139 | C19orf24 | 3 | 0.0824 | 0.6952 | -0.0580 | 0.4347 |
| 140 | C19orf48 | 5 | 0.5322 | 0.9804 | -0.5179 | 0.0000 |
| 141 | C1orf159 | 2 | 0.0094 | 0.9291 | 0.0094 | 0.9291 |
| 142 | C1orf43 | 2 | 0.2792 | 0.0070 | 0.2792 | 0.0070 |
| 143 | C1RL | 2 | -0.0416 | 0.6941 | -0.0416 | 0.6941 |
| 144 | C22orf46 | 3 | 0.1493 | 0.5558 | 0.0622 | 0.1554 |
| 145 | C2orf68 | 2 | 0.0339 | 0.7486 | 0.0339 | 0.7486 |
| 146 | C3orf38 | 2 | 0.3117 | 0.0025 | 0.3117 | 0.0025 |
| 147 | C6orf106 | 3 | 0.1453 | 0.3602 | -0.1059 | 0.1670 |
| 148 | C6orf47 | 3 | 0.1921 | 0.5674 | -0.0604 | 0.0665 |
| 149 | C6orf89 | 3 | 0.0827 | 0.7495 | -0.4090 | 0.0001 |
| 150 | CA12 | 3 | 0.2475 | 0.0317 | -0.2753 | 0.0079 |
| 151 | CABIN1 | 2 | 0.1454 | 0.1667 | 0.1454 | 0.1667 |
| 152 | CACUL1 | 2 | -0.1074 | 0.3082 | -0.1074 | 0.3082 |
| 153 | CALD1 | 2 | 0.0664 | 0.5296 | 0.0664 | 0.5296 |
| 154 | CAMKK1 | 2 | -0.2248 | 0.0312 | -0.2248 | 0.0312 |
| 155 | CAMSAP3 | 2 | -0.1786 | 0.0886 | -0.1786 | 0.0886 |
| 156 | CANT1 | 3 | -0.0430 | 0.6840 | -0.0898 | 0.3949 |
| 157 | CANX | 3 | 0.4663 | 0.8349 | -0.0220 | 0.0000 |
| 158 | CARD8 | 2 | 0.3067 | 0.0029 | 0.3067 | 0.0029 |
| 159 | CARS | 2 | -0.0293 | 0.7815 | -0.0293 | 0.7815 |
| 160 | CASC3 | 3 | 0.1470 | 0.6866 | -0.1214 | 0.1621 |
| 161 | CASKIN2 | 4 | 0.5114 | 0.9808 | -0.1060 | 0.0000 |
| 162 | CAVIN1 | 2 | 0.0375 | 0.7225 | 0.0375 | 0.7225 |
| 163 | CBR1 | 2 | -0.1435 | 0.1722 | -0.1435 | 0.1722 |
| 164 | CBX2 | 4 | 0.2953 | 0.6845 | -0.3077 | 0.0028 |
| 165 | CBX4 | 2 | 0.2797 | 0.0069 | 0.2797 | 0.0069 |
| 166 | CBX8 | 2 | -0.0730 | 0.4890 | -0.0730 | 0.4890 |
| 167 | CCDC127 | 2 | -0.2851 | 0.0059 | -0.2851 | 0.0059 |
| 168 | CCDC130 | 3 | 0.4351 | 0.1207 | -0.2207 | 0.0000 |
| 169 | CCDC14 | 2 | -0.0350 | 0.7407 | -0.0350 | 0.7407 |
| 170 | CCDC51 | 2 | 0.3532 | 0.0006 | 0.3532 | 0.0006 |
| 171 | CCDC57 | 4 | 0.1815 | 0.8688 | -0.2203 | 0.0349 |
| 172 | CCDC59 | 2 | -0.2682 | 0.0097 | -0.2682 | 0.0097 |
| 173 | CCDC71 | 2 | -0.1012 | 0.3371 | -0.1012 | 0.3371 |
| 174 | CCDC80 | 4 | 0.1719 | 0.4925 | -0.2995 | 0.0037 |
| 175 | CCDC88C | 3 | 0.2291 | 0.9000 | 0.0133 | 0.0280 |
| 176 | CCNT1 | 2 | -0.1831 | 0.0806 | -0.1831 | 0.0806 |
| 177 | CCNT2 | 2 | 0.3374 | 0.0010 | 0.3374 | 0.0010 |
| 178 | CD320 | 3 | 0.2019 | 0.9778 | -0.1294 | 0.0536 |
| 179 | CD3EAP | 2 | -0.2670 | 0.0101 | -0.2670 | 0.0101 |
| 180 | CD81 | 2 | 0.0535 | 0.6128 | 0.0535 | 0.6128 |
| 181 | CD99L2 | 2 | -0.0445 | 0.6739 | -0.0445 | 0.6739 |
| 182 | CDC37 | 2 | -0.0494 | 0.6401 | -0.0494 | 0.6401 |
| 183 | CDC42EP3 | 4 | 0.4782 | 0.4999 | -0.2178 | 0.0000 |
| 184 | CDC42EP4 | 2 | 0.1866 | 0.0749 | 0.1866 | 0.0749 |
| 185 | CDCA2 | 2 | -0.1117 | 0.2893 | -0.1117 | 0.2893 |
| 186 | CDK12 | 6 | 0.2786 | 0.9823 | -0.5514 | 0.0000 |
| 187 | CDK13 | 4 | 0.7665 | 0.6991 | -0.1662 | 0.0000 |
| 188 | CDYL | 4 | 0.7498 | 0.4318 | -0.1897 | 0.0000 |
| 189 | CELSR1 | 4 | 0.1723 | 0.8964 | -0.2906 | 0.0050 |
| 190 | CELSR2 | 3 | -0.0124 | 0.9064 | -0.2052 | 0.0498 |
| 191 | CELSR3 | 2 | -0.3586 | 0.0004 | -0.3586 | 0.0004 |
| 192 | CENPB | 2 | 0.2992 | 0.0038 | 0.2992 | 0.0038 |
| 193 | CENPBD1 | 3 | -0.0353 | 0.7381 | -0.1207 | 0.2518 |
| 194 | CENPBD1P1 | 2 | 0.4382 | 0.0000 | 0.4382 | 0.0000 |
| 195 | CENPO | 2 | -0.0107 | 0.9195 | -0.0107 | 0.9195 |
| 196 | CENPX | 3 | 0.2061 | 0.8697 | 0.0173 | 0.0487 |
| 197 | CEP152 | 2 | -0.0325 | 0.7583 | -0.0325 | 0.7583 |
| 198 | CEP170B | 4 | 0.0854 | 0.7688 | -0.0350 | 0.4181 |
| 199 | CEP250 | 6 | 0.1951 | 0.9461 | -0.2120 | 0.0425 |
| 200 | CERS2 | 2 | -0.0097 | 0.9271 | -0.0097 | 0.9271 |
| 201 | CHAF1A | 2 | -0.0155 | 0.8831 | -0.0155 | 0.8831 |
| 202 | CHAMP1 | 3 | -0.0070 | 0.9472 | -0.1373 | 0.1919 |
| 203 | CHD6 | 2 | 0.2081 | 0.0465 | 0.2081 | 0.0465 |
| 204 | CHD7 | 5 | 0.2173 | 0.8442 | -0.2157 | 0.0375 |
| 205 | CHD9 | 2 | 0.3762 | 0.0002 | 0.3762 | 0.0002 |
| 206 | CHERP | 2 | -0.1392 | 0.1856 | -0.1392 | 0.1856 |
| 207 | CHID1 | 2 | -0.0441 | 0.6765 | -0.0441 | 0.6765 |
| 208 | CHML | 4 | 0.3384 | 0.7884 | -0.0643 | 0.0010 |
| 209 | CHMP1A | 4 | 0.1333 | 0.9139 | -0.0653 | 0.2054 |
| 210 | CHPF | 5 | 0.6727 | 0.9515 | -0.2454 | 0.0000 |
| 211 | CHPF2 | 4 | 0.5770 | 0.9106 | -0.1908 | 0.0000 |
| 212 | CHRAC1 | 2 | 0.6211 | 0.0000 | 0.6211 | 0.0000 |
| 213 | CHST10 | 3 | 0.1556 | 0.3246 | -0.1123 | 0.1385 |
| 214 | CHST11 | 2 | 0.2919 | 0.0048 | 0.2919 | 0.0048 |
| 215 | CHST15 | 2 | -0.1186 | 0.2603 | -0.1186 | 0.2603 |
| 216 | CHST2 | 3 | 0.0715 | 0.6603 | -0.3694 | 0.0003 |
| 217 | CHST3 | 4 | 0.1422 | 0.3595 | -0.2001 | 0.0559 |
| 218 | CHSY1 | 4 | 0.2252 | 0.7327 | -0.1276 | 0.0309 |
| 219 | CIC | 6 | 0.0993 | 0.9661 | -0.2353 | 0.0239 |
| 220 | CIZ1 | 2 | -0.1542 | 0.1423 | -0.1542 | 0.1423 |
| 221 | CKAP4 | 3 | 0.1915 | 0.8494 | -0.0843 | 0.0675 |
| 222 | CLEC16A | 3 | -0.0946 | 0.3696 | -0.1851 | 0.0773 |
| 223 | CLIP2 | 4 | 0.1468 | 0.9565 | -0.1214 | 0.1627 |
| 224 | CLN8 | 2 | 0.0432 | 0.6825 | 0.0432 | 0.6825 |
| 225 | CLP1 | 2 | 0.1355 | 0.1978 | 0.1355 | 0.1978 |
| 226 | CLSPN | 2 | 0.3127 | 0.0024 | 0.3127 | 0.0024 |
| 227 | CLU | 5 | 0.4984 | 0.8709 | -0.0662 | 0.0000 |
| 228 | CLUH | 2 | 0.2470 | 0.0176 | 0.2470 | 0.0176 |
| 229 | CMTM1 | 2 | -0.1024 | 0.3312 | -0.1024 | 0.3312 |
| 230 | CNDP2 | 2 | 0.9345 | 0.0000 | 0.9345 | 0.0000 |
| 231 | CNPY3 | 3 | -0.0573 | 0.5878 | -0.2658 | 0.0105 |
| 232 | COA1 | 2 | 0.0483 | 0.6478 | 0.0483 | 0.6478 |
| 233 | COA4 | 2 | 0.1159 | 0.2711 | 0.1159 | 0.2711 |
| 234 | COBL | 2 | -0.3131 | 0.0024 | -0.3131 | 0.0024 |
| 235 | COL5A1 | 2 | 0.4140 | 0.0000 | 0.4140 | 0.0000 |
| 236 | COL6A1 | 2 | -0.1192 | 0.2576 | -0.1192 | 0.2576 |
| 237 | COLGALT1 | 2 | 0.2077 | 0.0469 | 0.2077 | 0.0469 |
| 238 | COMMD5 | 2 | 0.0230 | 0.8281 | 0.0230 | 0.8281 |
| 239 | CORO1C | 2 | -0.2964 | 0.0041 | -0.2964 | 0.0041 |
| 240 | CPTP | 3 | 0.2193 | 0.7861 | -0.0326 | 0.0357 |
| 241 | CRAMP1 | 5 | 0.3130 | 0.9206 | -0.2344 | 0.0024 |
| 242 | CREB1 | 2 | -0.1273 | 0.2267 | -0.1273 | 0.2267 |
| 243 | CREBBP | 2 | -0.5112 | 0.0000 | -0.5112 | 0.0000 |
| 244 | CREG1 | 2 | 0.2588 | 0.0128 | 0.2588 | 0.0128 |
| 245 | CRK | 2 | -0.1139 | 0.2797 | -0.1139 | 0.2797 |
| 246 | CRKL | 4 | 0.2566 | 0.8616 | -0.3382 | 0.0010 |
| 247 | CRTC3 | 3 | 0.2836 | 0.5969 | 0.0559 | 0.0062 |
| 248 | CRYBG1 | 2 | -0.2488 | 0.0168 | -0.2488 | 0.0168 |
| 249 | CSF1 | 6 | 0.4663 | 0.9827 | -0.3171 | 0.0000 |
| 250 | CSGALNACT1 | 2 | 0.1457 | 0.1657 | 0.1457 | 0.1657 |
| 251 | CSNK1D | 4 | 0.4627 | 0.2380 | -0.3132 | 0.0000 |
| 252 | CSNK1G1 | 4 | 0.3440 | 0.9374 | -0.0693 | 0.0008 |
| 253 | CSRNP1 | 3 | 0.1597 | 0.6408 | -0.0516 | 0.1283 |
| 254 | CSRNP2 | 2 | 0.1313 | 0.2121 | 0.1313 | 0.2121 |
| 255 | CSTF2T | 4 | 0.1497 | 0.9968 | -0.0212 | 0.1542 |
| 256 | CTDP1 | 2 | -0.2234 | 0.0323 | -0.2234 | 0.0323 |
| 257 | CTR9 | 3 | 0.1308 | 0.7895 | -0.1812 | 0.0839 |
| 258 | CTSB | 2 | -0.1220 | 0.2468 | -0.1220 | 0.2468 |
| 259 | CTTN | 2 | 0.0605 | 0.5671 | 0.0605 | 0.5671 |
| 260 | CTTNBP2NL | 2 | 0.3960 | 0.0001 | 0.3960 | 0.0001 |
| 261 | CUL4A | 2 | -0.0915 | 0.3859 | -0.0915 | 0.3859 |
| 262 | CUX1 | 4 | 0.1494 | 0.7221 | -0.1934 | 0.0648 |
| 263 | CYBC1 | 4 | 0.1701 | 0.9254 | -0.2633 | 0.0112 |
| 264 | CYREN | 3 | 0.0143 | 0.8927 | -0.4285 | 0.0000 |
| 265 | DAB2 | 2 | 0.0873 | 0.4082 | 0.0873 | 0.4082 |
| 266 | DAG1 | 5 | 0.3510 | 0.9492 | -0.1871 | 0.0006 |
| 267 | DAGLB | 2 | -0.0450 | 0.6703 | -0.0450 | 0.6703 |
| 268 | DAP | 2 | -0.2537 | 0.0147 | -0.2537 | 0.0147 |
| 269 | DAXX | 2 | 0.2732 | 0.0084 | 0.2732 | 0.0084 |
| 270 | DBNDD1 | 3 | -0.0205 | 0.8464 | -0.0751 | 0.4765 |
| 271 | DBT | 2 | 0.2976 | 0.0040 | 0.2976 | 0.0040 |
| 272 | DCAF5 | 3 | 0.1699 | 0.8208 | -0.0391 | 0.1054 |
| 273 | DCAF8 | 2 | 0.2328 | 0.0256 | 0.2328 | 0.0256 |
| 274 | DCBLD1 | 4 | 0.2285 | 0.9739 | -0.0673 | 0.0285 |
| 275 | DCTN4 | 2 | -0.1085 | 0.3031 | -0.1085 | 0.3031 |
| 276 | DCTN5 | 2 | 0.2398 | 0.0213 | 0.2398 | 0.0213 |
| 277 | DCUN1D2 | 3 | 0.3688 | 0.2843 | -0.1324 | 0.0003 |
| 278 | DDIT3 | 2 | 0.1226 | 0.2442 | 0.1226 | 0.2442 |
| 279 | DDOST | 3 | 0.2479 | 0.3452 | -0.0995 | 0.0172 |
| 280 | DDX17 | 2 | 0.1251 | 0.2347 | 0.1251 | 0.2347 |
| 281 | DDX23 | 2 | 0.2036 | 0.0516 | 0.2036 | 0.0516 |
| 282 | DDX28 | 3 | 0.2058 | 0.6784 | -0.0438 | 0.0490 |
| 283 | DEDD2 | 2 | 0.1097 | 0.2978 | 0.1097 | 0.2978 |
| 284 | DENND5B | 2 | -0.0099 | 0.9253 | -0.0099 | 0.9253 |
| 285 | DGCR2 | 4 | 0.3678 | 0.9788 | -0.0382 | 0.0003 |
| 286 | DGCR8 | 4 | 0.1776 | 0.7241 | -0.2214 | 0.0340 |
| 287 | DHCR24 | 3 | 0.2637 | 0.1113 | -0.2845 | 0.0060 |
| 288 | DHX8 | 3 | 0.1174 | 0.8256 | 0.0233 | 0.2651 |
| 289 | DIDO1 | 5 | 0.2512 | 0.9738 | -0.2692 | 0.0095 |
| 290 | DLG5 | 5 | 0.2012 | 0.8076 | -0.2505 | 0.0160 |
| 291 | DLX4 | 2 | 0.3784 | 0.0002 | 0.3784 | 0.0002 |
| 292 | DNAAF5 | 2 | -0.5651 | 0.0000 | -0.5651 | 0.0000 |
| 293 | DNAJB1 | 4 | 0.2197 | 0.7215 | -0.2462 | 0.0180 |
| 294 | DNAJB4 | 3 | 0.2128 | 0.7572 | -0.0327 | 0.0417 |
| 295 | DNAJC14 | 3 | 0.0168 | 0.8739 | -0.1438 | 0.1715 |
| 296 | DNM2 | 2 | -0.1832 | 0.0804 | -0.1832 | 0.0804 |
| 297 | DOCK3 | 2 | -0.1771 | 0.0913 | -0.1771 | 0.0913 |
| 298 | DOLK | 2 | -0.3265 | 0.0015 | -0.3265 | 0.0015 |
| 299 | DSE | 3 | 0.1303 | 0.2588 | -0.1910 | 0.0683 |
| 300 | DSP | 3 | 0.0579 | 0.6646 | -0.2629 | 0.0113 |
| 301 | DUS1L | 3 | 0.3639 | 0.3608 | -0.4566 | 0.0000 |
| 302 | DUSP16 | 3 | 0.1769 | 0.9354 | -0.1806 | 0.0850 |
| 303 | DUSP8 | 2 | 0.8143 | 0.0000 | 0.8143 | 0.0000 |
| 304 | DYRK1A | 2 | 0.2066 | 0.0481 | 0.2066 | 0.0481 |
| 305 | EARS2 | 3 | 0.0363 | 0.7312 | -0.1433 | 0.1729 |
| 306 | EBLN3P | 2 | 0.2456 | 0.0183 | 0.2456 | 0.0183 |
| 307 | EBP | 2 | 0.5054 | 0.0000 | 0.5054 | 0.0000 |
| 308 | ECE1 | 2 | 0.4849 | 0.0000 | 0.4849 | 0.0000 |
| 309 | EDC3 | 2 | -0.1732 | 0.0987 | -0.1732 | 0.0987 |
| 310 | EDF1 | 2 | 0.2531 | 0.0149 | 0.2531 | 0.0149 |
| 311 | EEF1A1 | 2 | 0.0450 | 0.6703 | 0.0450 | 0.6703 |
| 312 | EEF1AKNMT | 2 | 0.0832 | 0.4305 | 0.0832 | 0.4305 |
| 313 | EEF2 | 4 | 0.2030 | 0.9975 | -0.1481 | 0.0523 |
| 314 | EFNB2 | 2 | -0.1053 | 0.3179 | -0.1053 | 0.3179 |
| 315 | EGR1 | 3 | -0.0702 | 0.5060 | -0.1762 | 0.0929 |
| 316 | EGR3 | 3 | 0.2355 | 0.8415 | -0.0546 | 0.0238 |
| 317 | EHD2 | 3 | 0.1838 | 0.9321 | 0.0090 | 0.0794 |
| 318 | EIF1AD | 2 | -0.0079 | 0.9407 | -0.0079 | 0.9407 |
| 319 | EIF4EBP1 | 3 | 0.2131 | 0.6228 | -0.1681 | 0.0414 |
| 320 | EIF4G1 | 2 | 0.3480 | 0.0007 | 0.3480 | 0.0007 |
| 321 | EIF4G2 | 2 | 0.4241 | 0.0000 | 0.4241 | 0.0000 |
| 322 | ELF4 | 2 | -0.1732 | 0.0988 | -0.1732 | 0.0988 |
| 323 | ELK3 | 2 | 0.0939 | 0.3735 | 0.0939 | 0.3735 |
| 324 | ELK4 | 2 | 0.1227 | 0.2441 | 0.1227 | 0.2441 |
| 325 | ELMSAN1 | 2 | 0.0228 | 0.8293 | 0.0228 | 0.8293 |
| 326 | ELOA | 3 | 0.5061 | 0.0036 | 0.3005 | 0.0000 |
| 327 | EMD | 2 | -0.1230 | 0.2428 | -0.1230 | 0.2428 |
| 328 | ENG | 2 | 0.2080 | 0.0466 | 0.2080 | 0.0466 |
| 329 | ENTPD4 | 3 | 0.2363 | 0.2566 | -0.1958 | 0.0234 |
| 330 | EP300 | 5 | 0.4244 | 0.8202 | -0.4327 | 0.0000 |
| 331 | EP400 | 2 | 0.1863 | 0.0755 | 0.1863 | 0.0755 |
| 332 | EPG5 | 3 | 0.1579 | 0.8589 | -0.0433 | 0.1327 |
| 333 | EPHB2 | 2 | -0.1676 | 0.1104 | -0.1676 | 0.1104 |
| 334 | EPHB4 | 2 | 0.1798 | 0.0863 | 0.1798 | 0.0863 |
| 335 | EPN1 | 2 | 0.1101 | 0.2961 | 0.1101 | 0.2961 |
| 336 | EPN2 | 2 | 0.1009 | 0.3387 | 0.1009 | 0.3387 |
| 337 | EPOP | 3 | 0.0452 | 0.6691 | -0.1944 | 0.0633 |
| 338 | EPOR | 5 | 0.2175 | 0.9551 | -0.2910 | 0.0049 |
| 339 | ERBB2 | 4 | 0.1097 | 0.9854 | -0.3108 | 0.0026 |
| 340 | ERC1 | 2 | -0.0147 | 0.8892 | -0.0147 | 0.8892 |
| 341 | ERCC1 | 2 | -0.1031 | 0.3283 | -0.1031 | 0.3283 |
| 342 | ERCC4 | 2 | 0.0471 | 0.6559 | 0.0471 | 0.6559 |
| 343 | ERCC6L | 2 | -0.2902 | 0.0050 | -0.2902 | 0.0050 |
| 344 | ERF | 2 | 0.0688 | 0.5144 | 0.0688 | 0.5144 |
| 345 | ERRFI1 | 4 | 0.0402 | 0.9087 | -0.4258 | 0.0000 |
| 346 | EVC | 2 | 0.0605 | 0.5670 | 0.0605 | 0.5670 |
| 347 | EXD3 | 2 | 0.0696 | 0.5097 | 0.0696 | 0.5097 |
| 348 | EXO5 | 2 | -0.1854 | 0.0768 | -0.1854 | 0.0768 |
| 349 | EXOC3 | 2 | 0.0493 | 0.6410 | 0.0493 | 0.6410 |
| 350 | EXOC8 | 2 | -0.2313 | 0.0265 | -0.2313 | 0.0265 |
| 351 | EXOSC2 | 2 | -0.1044 | 0.3218 | -0.1044 | 0.3218 |
| 352 | EXT1 | 4 | 0.2969 | 0.9376 | -0.1939 | 0.0041 |
| 353 | EXT2 | 2 | 0.0570 | 0.5893 | 0.0570 | 0.5893 |
| 354 | F2R | 3 | 0.2061 | 0.5679 | -0.1831 | 0.0487 |
| 355 | FAF2 | 2 | -0.0108 | 0.9184 | -0.0108 | 0.9184 |
| 356 | FAM122A | 2 | -0.0664 | 0.5295 | -0.0664 | 0.5295 |
| 357 | FAM13A | 3 | 0.0275 | 0.8536 | -0.2247 | 0.0313 |
| 358 | FAM168A | 3 | 0.1239 | 0.7655 | 0.0315 | 0.2395 |
| 359 | FAM171A1 | 4 | 0.1913 | 0.7818 | -0.1740 | 0.0677 |
| 360 | FAM193B | 4 | 0.1170 | 0.9794 | -0.1537 | 0.1435 |
| 361 | FAM208B | 8 | 0.5138 | 0.8759 | -0.3960 | 0.0000 |
| 362 | FAM20C | 2 | 0.4293 | 0.0000 | 0.4293 | 0.0000 |
| 363 | FAM32A | 4 | 0.2687 | 0.8009 | -0.0913 | 0.0096 |
| 364 | FAM53B | 4 | 0.2409 | 0.7190 | -0.0380 | 0.0207 |
| 365 | FAM53C | 2 | 0.0798 | 0.4494 | 0.0798 | 0.4494 |
| 366 | FAM78A | 2 | -0.1415 | 0.1785 | -0.1415 | 0.1785 |
| 367 | FAM83D | 2 | 0.2307 | 0.0270 | 0.2307 | 0.0270 |
| 368 | FAM83G | 4 | 0.2060 | 0.9627 | -0.0856 | 0.0488 |
| 369 | FAM83H | 2 | -0.0500 | 0.6360 | -0.0500 | 0.6360 |
| 370 | FANCE | 3 | 0.2671 | 0.3899 | -0.2410 | 0.0101 |
| 371 | FASN | 3 | 0.2435 | 0.2275 | -0.1542 | 0.0194 |
| 372 | FASTKD5 | 3 | 0.2016 | 0.6170 | -0.3241 | 0.0016 |
| 373 | FAT1 | 10 | 0.4913 | 0.9925 | -0.4218 | 0.0000 |
| 374 | FBXL14 | 2 | 0.0503 | 0.6340 | 0.0503 | 0.6340 |
| 375 | FBXL19 | 3 | 0.4986 | 0.9765 | -0.0291 | 0.0000 |
| 376 | FBXL5 | 2 | 0.2419 | 0.0202 | 0.2419 | 0.0202 |
| 377 | FBXO10 | 2 | -0.0802 | 0.4472 | -0.0802 | 0.4472 |
| 378 | FBXO17 | 2 | -0.2577 | 0.0131 | -0.2577 | 0.0131 |
| 379 | FBXO28 | 3 | 0.4551 | 0.8222 | 0.0238 | 0.0000 |
| 380 | FBXO31 | 5 | 0.3588 | 0.9676 | -0.2996 | 0.0004 |
| 381 | FBXO42 | 2 | 0.2570 | 0.0134 | 0.2570 | 0.0134 |
| 382 | FBXO46 | 4 | 0.7137 | 0.8831 | -0.0601 | 0.0000 |
| 383 | FBXO5 | 2 | 0.4252 | 0.0000 | 0.4252 | 0.0000 |
| 384 | FCMR | 2 | -0.1674 | 0.1107 | -0.1674 | 0.1107 |
| 385 | FDFT1 | 2 | 0.1259 | 0.2319 | 0.1259 | 0.2319 |
| 386 | FDX2 | 2 | -0.1962 | 0.0609 | -0.1962 | 0.0609 |
| 387 | FEM1A | 2 | -0.0273 | 0.7959 | -0.0273 | 0.7959 |
| 388 | FEM1B | 3 | 0.0189 | 0.8584 | -0.3218 | 0.0018 |
| 389 | FEM1C | 2 | -0.0769 | 0.4663 | -0.0769 | 0.4663 |
| 390 | FJX1 | 3 | 0.0285 | 0.7874 | -0.3548 | 0.0005 |
| 391 | FKBP10 | 2 | -0.1161 | 0.2706 | -0.1161 | 0.2706 |
| 392 | FKRP | 4 | 0.2711 | 0.9797 | -0.1811 | 0.0090 |
| 393 | FLCN | 2 | -0.1097 | 0.2979 | -0.1097 | 0.2979 |
| 394 | FLYWCH1 | 2 | -0.2672 | 0.0100 | -0.2672 | 0.0100 |
| 395 | FNDC10 | 2 | -0.1755 | 0.0943 | -0.1755 | 0.0943 |
| 396 | FOXA1 | 2 | -0.1805 | 0.0850 | -0.1805 | 0.0850 |
| 397 | FOXK1 | 9 | 0.5602 | 0.9490 | -0.3826 | 0.0000 |
| 398 | FOXK2 | 4 | 0.1063 | 0.9394 | -0.1962 | 0.0609 |
| 399 | FOXM1 | 5 | 0.4211 | 0.8133 | -0.2497 | 0.0000 |
| 400 | FOXO1 | 2 | 0.1527 | 0.1460 | 0.1527 | 0.1460 |
| 401 | FRS2 | 2 | 0.1512 | 0.1503 | 0.1512 | 0.1503 |
| 402 | FSTL3 | 2 | 0.1081 | 0.3052 | 0.1081 | 0.3052 |
| 403 | FUT10 | 2 | 0.1817 | 0.0830 | 0.1817 | 0.0830 |
| 404 | FYCO1 | 2 | -0.1669 | 0.1118 | -0.1669 | 0.1118 |
| 405 | FYN | 2 | 0.2432 | 0.0195 | 0.2432 | 0.0195 |
| 406 | FZD1 | 2 | 0.0809 | 0.4434 | 0.0809 | 0.4434 |
| 407 | FZD6 | 3 | 0.1729 | 0.8809 | -0.3335 | 0.0012 |
| 408 | GADD45B | 2 | 0.1612 | 0.1247 | 0.1612 | 0.1247 |
| 409 | GAPDH | 2 | 0.3420 | 0.0008 | 0.3420 | 0.0008 |
| 410 | GAS2L3 | 3 | 0.2356 | 0.0544 | -0.2786 | 0.0072 |
| 411 | GATAD2A | 2 | 0.2657 | 0.0105 | 0.2657 | 0.0105 |
| 412 | GBA2 | 4 | 0.4102 | 0.9593 | -0.1921 | 0.0000 |
| 413 | GCC1 | 4 | 0.2703 | 0.8039 | -0.2827 | 0.0063 |
| 414 | GCNT3 | 3 | 0.1391 | 0.2917 | -0.1787 | 0.0883 |
| 415 | GDF11 | 3 | 0.1142 | 0.2784 | -0.2360 | 0.0235 |
| 416 | GEMIN4 | 6 | 0.3171 | 0.9353 | -0.2288 | 0.0021 |
| 417 | GEMIN5 | 2 | -0.0734 | 0.4871 | -0.0734 | 0.4871 |
| 418 | GGA3 | 4 | 0.2662 | 0.7947 | -0.2955 | 0.0042 |
| 419 | GIPC1 | 2 | 0.0420 | 0.6910 | 0.0420 | 0.6910 |
| 420 | GJC1 | 2 | -0.0747 | 0.4793 | -0.0747 | 0.4793 |
| 421 | GLG1 | 4 | 0.2220 | 0.4488 | -0.3259 | 0.0015 |
| 422 | GNA12 | 4 | 0.3050 | 0.3564 | -0.2170 | 0.0031 |
| 423 | GOLGA3 | 3 | 0.0317 | 0.9569 | -0.0369 | 0.7268 |
| 424 | GON4L | 2 | 0.0502 | 0.6348 | 0.0502 | 0.6348 |
| 425 | GOSR1 | 2 | 0.0749 | 0.4777 | 0.0749 | 0.4777 |
| 426 | GPAT4 | 2 | -0.2586 | 0.0128 | -0.2586 | 0.0128 |
| 427 | GPATCH8 | 2 | 0.0860 | 0.4152 | 0.0860 | 0.4152 |
| 428 | GPRIN1 | 4 | 0.1313 | 0.9907 | -0.1543 | 0.1420 |
| 429 | GPS1 | 2 | 0.1785 | 0.0886 | 0.1785 | 0.0886 |
| 430 | GPX2 | 2 | 0.1274 | 0.2262 | 0.1274 | 0.2262 |
| 431 | GRAMD4 | 3 | 0.1232 | 0.2902 | -0.1768 | 0.0919 |
| 432 | GRB10 | 2 | 0.1375 | 0.1912 | 0.1375 | 0.1912 |
| 433 | GSE1 | 2 | -0.0796 | 0.4506 | -0.0796 | 0.4506 |
| 434 | GSPT1 | 2 | -0.1920 | 0.0668 | -0.1920 | 0.0668 |
| 435 | GTF3C2 | 2 | 0.1882 | 0.0724 | 0.1882 | 0.0724 |
| 436 | GTF3C4 | 2 | 0.0079 | 0.9403 | 0.0079 | 0.9403 |
| 437 | GTF3C5 | 2 | -0.0735 | 0.4861 | -0.0735 | 0.4861 |
| 438 | GUCD1 | 2 | -0.3726 | 0.0003 | -0.3726 | 0.0003 |
| 439 | GUK1 | 2 | -0.4116 | 0.0000 | -0.4116 | 0.0000 |
| 440 | GVINP1 | 2 | 0.2115 | 0.0430 | 0.2115 | 0.0430 |
| 441 | H1F0 | 3 | 0.2930 | 0.7662 | -0.0314 | 0.0046 |
| 442 | H6PD | 4 | 0.1698 | 0.4039 | -0.4773 | 0.0000 |
| 443 | HARS | 2 | 0.5341 | 0.0000 | 0.5341 | 0.0000 |
| 444 | HCFC1 | 2 | -0.1710 | 0.1032 | -0.1710 | 0.1032 |
| 445 | HCFC1R1 | 2 | 0.0439 | 0.6777 | 0.0439 | 0.6777 |
| 446 | HDGFL2 | 2 | 0.1838 | 0.0795 | 0.1838 | 0.0795 |
| 447 | HDHD3 | 5 | 0.2006 | 0.9515 | -0.2046 | 0.0504 |
| 448 | HDLBP | 2 | -0.0504 | 0.6335 | -0.0504 | 0.6335 |
| 449 | HEATR6 | 2 | 0.3129 | 0.0024 | 0.3129 | 0.0024 |
| 450 | HECA | 3 | 0.0410 | 0.6978 | -0.2305 | 0.0271 |
| 451 | HEG1 | 2 | -0.0569 | 0.5901 | -0.0569 | 0.5901 |
| 452 | HELZ2 | 2 | -0.0341 | 0.7467 | -0.0341 | 0.7467 |
| 453 | HEMK1 | 2 | 0.2693 | 0.0094 | 0.2693 | 0.0094 |
| 454 | HERC2 | 2 | 0.2419 | 0.0202 | 0.2419 | 0.0202 |
| 455 | HEXIM1 | 2 | 0.2446 | 0.0188 | 0.2446 | 0.0188 |
| 456 | HIC2 | 2 | 0.3371 | 0.0010 | 0.3371 | 0.0010 |
| 457 | HILPDA | 2 | 0.1471 | 0.1617 | 0.1471 | 0.1617 |
| 458 | HIP1 | 2 | 0.5523 | 0.0000 | 0.5523 | 0.0000 |
| 459 | HIPK2 | 3 | 0.1510 | 0.6313 | -0.2274 | 0.0293 |
| 460 | HIRIP3 | 3 | 0.3070 | 0.9863 | -0.0755 | 0.0029 |
| 461 | HIST1H3B | 2 | -0.0421 | 0.6900 | -0.0421 | 0.6900 |
| 462 | HIVEP1 | 2 | 0.0145 | 0.8907 | 0.0145 | 0.8907 |
| 463 | HIVEP2 | 5 | 0.3002 | 0.8720 | -0.1397 | 0.0036 |
| 464 | HMOX2 | 2 | -0.0900 | 0.3935 | -0.0900 | 0.3935 |
| 465 | HOMER1 | 2 | 0.0201 | 0.8494 | 0.0201 | 0.8494 |
| 466 | HPS6 | 6 | 0.3640 | 0.8201 | -0.0950 | 0.0004 |
| 467 | HS6ST1 | 3 | 0.0316 | 0.9239 | -0.0101 | 0.7647 |
| 468 | HSD17B1 | 2 | 0.2155 | 0.0391 | 0.2155 | 0.0391 |
| 469 | HSP90AB1 | 3 | 0.4226 | 0.4341 | 0.0825 | 0.0000 |
| 470 | HSP90B1 | 2 | 0.2179 | 0.0370 | 0.2179 | 0.0370 |
| 471 | ICE1 | 4 | 0.4038 | 0.9494 | -0.1134 | 0.0001 |
| 472 | ICMT | 3 | 0.1843 | 0.3984 | -0.1510 | 0.0786 |
| 473 | IFIT5 | 3 | 0.4108 | 0.5118 | -0.2077 | 0.0000 |
| 474 | IFNAR2 | 5 | 0.3280 | 0.9747 | -0.0168 | 0.0014 |
| 475 | IGF1R | 5 | 0.2563 | 0.9349 | -0.5516 | 0.0000 |
| 476 | IGF2R | 2 | -0.0066 | 0.9503 | -0.0066 | 0.9503 |
| 477 | IL17RA | 2 | 0.2368 | 0.0231 | 0.2368 | 0.0231 |
| 478 | IL4R | 4 | 0.2508 | 0.8290 | -0.1698 | 0.0159 |
| 479 | IL6ST | 2 | -0.0065 | 0.9509 | -0.0065 | 0.9509 |
| 480 | IL7R | 2 | -0.2933 | 0.0046 | -0.2933 | 0.0046 |
| 481 | INKA2 | 2 | -0.2134 | 0.0411 | -0.2134 | 0.0411 |
| 482 | INO80C | 2 | 0.5490 | 0.0000 | 0.5490 | 0.0000 |
| 483 | INO80D | 2 | -0.1293 | 0.2191 | -0.1293 | 0.2191 |
| 484 | INTS11 | 2 | 0.2785 | 0.0072 | 0.2785 | 0.0072 |
| 485 | INTS5 | 3 | 0.0463 | 0.6615 | -0.3253 | 0.0016 |
| 486 | IP6K1 | 3 | -0.0156 | 0.8825 | -0.1708 | 0.1036 |
| 487 | IPO8 | 2 | -0.0825 | 0.4344 | -0.0825 | 0.4344 |
| 488 | IPO9 | 2 | 0.0471 | 0.6557 | 0.0471 | 0.6557 |
| 489 | IQCE | 2 | 0.1430 | 0.1738 | 0.1430 | 0.1738 |
| 490 | IQSEC2 | 2 | -0.2248 | 0.0312 | -0.2248 | 0.0312 |
| 491 | IRF2BP1 | 2 | 0.2620 | 0.0116 | 0.2620 | 0.0116 |
| 492 | IRF2BP2 | 3 | 0.2507 | 0.7277 | -0.1371 | 0.0160 |
| 493 | IRF2BPL | 2 | 0.1276 | 0.2253 | 0.1276 | 0.2253 |
| 494 | IRS1 | 2 | -0.0807 | 0.4442 | -0.0807 | 0.4442 |
| 495 | IRS4 | 4 | 0.2633 | 0.7262 | -0.2748 | 0.0080 |
| 496 | ISG20L2 | 2 | 0.1663 | 0.1132 | 0.1663 | 0.1132 |
| 497 | IST1 | 2 | 0.1447 | 0.1688 | 0.1447 | 0.1688 |
| 498 | ITGA5 | 6 | 0.4405 | 0.8428 | -0.5820 | 0.0000 |
| 499 | ITGB5 | 2 | 0.2208 | 0.0344 | 0.2208 | 0.0344 |
| 500 | ITPKB | 4 | 0.1611 | 0.7467 | -0.1352 | 0.1249 |
| 501 | ITPKC | 3 | 0.1456 | 0.7523 | -0.0624 | 0.1661 |
| 502 | ITPR2 | 2 | -0.0435 | 0.6803 | -0.0435 | 0.6803 |
| 503 | ITPRIPL2 | 3 | 0.1564 | 0.9064 | -0.4231 | 0.0000 |
| 504 | JADE2 | 5 | 0.1620 | 0.9594 | -0.2097 | 0.0448 |
| 505 | JARID2 | 2 | 0.1631 | 0.1203 | 0.1631 | 0.1203 |
| 506 | JRK | 4 | 0.2300 | 0.6951 | -0.4669 | 0.0000 |
| 507 | JUN | 2 | 0.5014 | 0.0000 | 0.5014 | 0.0000 |
| 508 | JUNB | 6 | 0.3996 | 0.9504 | -0.1701 | 0.0001 |
| 509 | KANK1 | 2 | 0.0684 | 0.5172 | 0.0684 | 0.5172 |
| 510 | KANK2 | 3 | -0.0127 | 0.9041 | -0.1810 | 0.0842 |
| 511 | KANSL3 | 2 | 0.3170 | 0.0021 | 0.3170 | 0.0021 |
| 512 | KAT6A | 5 | 0.4746 | 0.7246 | -0.2027 | 0.0000 |
| 513 | KBTBD4 | 2 | -0.2775 | 0.0074 | -0.2775 | 0.0074 |
| 514 | KBTBD7 | 2 | 0.0074 | 0.9443 | 0.0074 | 0.9443 |
| 515 | KCNAB2 | 3 | -0.1047 | 0.3204 | -0.2414 | 0.0205 |
| 516 | KCNK5 | 2 | 0.0163 | 0.8772 | 0.0163 | 0.8772 |
| 517 | KCTD10 | 2 | -0.0019 | 0.9854 | -0.0019 | 0.9854 |
| 518 | KCTD11 | 3 | 0.2105 | 0.1382 | -0.1642 | 0.0440 |
| 519 | KCTD15 | 3 | 0.1587 | 0.6917 | -0.0522 | 0.1307 |
| 520 | KCTD2 | 4 | 0.1075 | 0.7287 | -0.1726 | 0.1000 |
| 521 | KDELR2 | 2 | 0.0710 | 0.5013 | 0.0710 | 0.5013 |
| 522 | KDM2A | 3 | 0.4683 | 0.5004 | -0.1199 | 0.0000 |
| 523 | KDM3B | 3 | 0.1488 | 0.3405 | 0.1005 | 0.1570 |
| 524 | KDM6A | 4 | 0.2241 | 0.7957 | -0.4255 | 0.0000 |
| 525 | KHDC4 | 2 | 0.0495 | 0.6397 | 0.0495 | 0.6397 |
| 526 | KHNYN | 3 | 0.0466 | 0.7284 | -0.1296 | 0.2183 |
| 527 | KIAA0753 | 2 | -0.0665 | 0.5290 | -0.0665 | 0.5290 |
| 528 | KIAA0754 | 4 | 0.2563 | 0.8865 | -0.2608 | 0.0120 |
| 529 | KIAA1191 | 2 | 0.0521 | 0.6217 | 0.0521 | 0.6217 |
| 530 | KIAA1671 | 2 | -0.2191 | 0.0359 | -0.2191 | 0.0359 |
| 531 | KIAA1958 | 2 | 0.0339 | 0.7485 | 0.0339 | 0.7485 |
| 532 | KIAA2026 | 3 | 0.1496 | 0.4340 | -0.2319 | 0.0261 |
| 533 | KIDINS220 | 2 | 0.1735 | 0.0981 | 0.1735 | 0.0981 |
| 534 | KIF18A | 2 | -0.0455 | 0.6664 | -0.0455 | 0.6664 |
| 535 | KIF18B | 4 | 0.2256 | 0.8375 | -0.2378 | 0.0224 |
| 536 | KIF1B | 2 | -0.0133 | 0.9000 | -0.0133 | 0.9000 |
| 537 | KIF3C | 6 | 0.5594 | 0.8911 | -0.0922 | 0.0000 |
| 538 | KLF11 | 3 | 0.4806 | 0.7656 | -0.1756 | 0.0000 |
| 539 | KLF12 | 2 | 0.0852 | 0.4191 | 0.0852 | 0.4191 |
| 540 | KLF13 | 4 | 0.3498 | 0.1474 | -0.3414 | 0.0006 |
| 541 | KLF6 | 2 | -0.2438 | 0.0192 | -0.2438 | 0.0192 |
| 542 | KLHL18 | 4 | 0.1076 | 0.5813 | -0.3401 | 0.0009 |
| 543 | KLHL21 | 3 | 0.1735 | 0.8443 | -0.1014 | 0.0981 |
| 544 | KLHL22 | 3 | 0.1552 | 0.8874 | -0.0921 | 0.1395 |
| 545 | KMT2A | 4 | 0.4002 | 0.5632 | 0.0611 | 0.0001 |
| 546 | KMT2B | 5 | 0.4947 | 0.5044 | -0.1859 | 0.0000 |
| 547 | KMT2C | 4 | 0.2726 | 0.7882 | -0.2480 | 0.0086 |
| 548 | KMT2D | 7 | 0.3485 | 0.9260 | -0.4370 | 0.0000 |
| 549 | KMT2E | 2 | 0.1433 | 0.1731 | 0.1433 | 0.1731 |
| 550 | KNOP1 | 4 | 0.1530 | 0.8058 | -0.1491 | 0.1453 |
| 551 | LAMC1 | 2 | -0.0219 | 0.8356 | -0.0219 | 0.8356 |
| 552 | LARP1 | 2 | 0.1213 | 0.2493 | 0.1213 | 0.2493 |
| 553 | LARS2 | 2 | -0.1977 | 0.0589 | -0.1977 | 0.0589 |
| 554 | LATS1 | 3 | 0.1266 | 0.7547 | -0.1352 | 0.1989 |
| 555 | LATS2 | 6 | 0.3644 | 0.8829 | -0.2049 | 0.0004 |
| 556 | LCMT2 | 2 | -0.2749 | 0.0080 | -0.2749 | 0.0080 |
| 557 | LDB1 | 2 | -0.0631 | 0.5505 | -0.0631 | 0.5505 |
| 558 | LDLR | 5 | 0.1602 | 0.8075 | -0.2963 | 0.0041 |
| 559 | LEF1 | 2 | 0.0613 | 0.5614 | 0.0613 | 0.5614 |
| 560 | LEMD3 | 2 | -0.1673 | 0.1110 | -0.1673 | 0.1110 |
| 561 | LETM1 | 2 | 0.0959 | 0.3630 | 0.0959 | 0.3630 |
| 562 | LGALS3BP | 3 | 0.0986 | 0.3937 | -0.2993 | 0.0038 |
| 563 | LHFPL2 | 3 | -0.0085 | 0.9356 | -0.2680 | 0.0098 |
| 564 | LMF2 | 2 | -0.1525 | 0.1466 | -0.1525 | 0.1466 |
| 565 | LMTK3 | 2 | 0.3698 | 0.0003 | 0.3698 | 0.0003 |
| 566 | LOC100287042 | 2 | -0.0764 | 0.4693 | -0.0764 | 0.4693 |
| 567 | LOC101927151 | 2 | 0.1823 | 0.0820 | 0.1823 | 0.0820 |
| 568 | LOC105371763 | 2 | 0.1439 | 0.1713 | 0.1439 | 0.1713 |
| 569 | LOC642852 | 3 | 0.0720 | 0.4955 | -0.2066 | 0.0481 |
| 570 | LOC728743 | 3 | 0.2013 | 0.6043 | 0.0547 | 0.0543 |
| 571 | LPCAT1 | 2 | -0.2536 | 0.0147 | -0.2536 | 0.0147 |
| 572 | LRFN1 | 2 | 0.2027 | 0.0526 | 0.2027 | 0.0526 |
| 573 | LRFN4 | 2 | 0.3173 | 0.0021 | 0.3173 | 0.0021 |
| 574 | LRP10 | 4 | 0.1194 | 0.7896 | -0.1481 | 0.1588 |
| 575 | LRRC14 | 6 | 0.5547 | 0.8380 | -0.2645 | 0.0000 |
| 576 | LRRC8A | 2 | 0.1395 | 0.1849 | 0.1395 | 0.1849 |
| 577 | LRRC8B | 2 | 0.5632 | 0.0000 | 0.5632 | 0.0000 |
| 578 | LRRC8D | 3 | 0.1469 | 0.3335 | -0.3369 | 0.0010 |
| 579 | LRRN3 | 3 | 0.3930 | 0.3145 | -0.2164 | 0.0001 |
| 580 | LSG1 | 2 | -0.1856 | 0.0766 | -0.1856 | 0.0766 |
| 581 | LURAP1L | 2 | 0.1929 | 0.0654 | 0.1929 | 0.0654 |
| 582 | LUZP1 | 5 | 0.0889 | 0.6277 | -0.2076 | 0.0471 |
| 583 | LYSMD1 | 3 | 0.0905 | 0.9169 | -0.1728 | 0.0996 |
| 584 | LYSMD4 | 2 | -0.1186 | 0.2601 | -0.1186 | 0.2601 |
| 585 | MALAT1 | 4 | 0.6735 | 0.3612 | 0.0963 | 0.0000 |
| 586 | MAML1 | 6 | 0.5076 | 0.6089 | -0.4042 | 0.0000 |
| 587 | MAML2 | 4 | 0.2578 | 0.8642 | -0.0181 | 0.0131 |
| 588 | MAN1B1 | 2 | -0.1577 | 0.1334 | -0.1577 | 0.1334 |
| 589 | MANBA | 2 | 0.2105 | 0.0441 | 0.2105 | 0.0441 |
| 590 | MAP1S | 3 | 0.7418 | 0.1157 | -0.1654 | 0.0000 |
| 591 | MAP3K11 | 2 | -0.0341 | 0.7467 | -0.0341 | 0.7467 |
| 592 | MAP4 | 6 | 0.7504 | 0.6257 | -0.0706 | 0.0000 |
| 593 | MAPK12 | 3 | 0.1747 | 0.1467 | -0.1630 | 0.0958 |
| 594 | MAPKBP1 | 2 | 0.2059 | 0.0490 | 0.2059 | 0.0490 |
| 595 | MARK2 | 2 | -0.1284 | 0.2225 | -0.1284 | 0.2225 |
| 596 | MARS2 | 2 | 0.0719 | 0.4958 | 0.0719 | 0.4958 |
| 597 | MAST2 | 3 | 0.3015 | 0.2890 | 0.1117 | 0.0035 |
| 598 | MAU2 | 2 | -0.0696 | 0.5098 | -0.0696 | 0.5098 |
| 599 | MAZ | 2 | 0.0844 | 0.4239 | 0.0844 | 0.4239 |
| 600 | MBP | 2 | 0.2020 | 0.0534 | 0.2020 | 0.0534 |
| 601 | MC1R | 3 | 0.7404 | 0.0053 | 0.2885 | 0.0000 |
| 602 | MCM3AP | 3 | 0.2905 | 0.6107 | -0.0538 | 0.0050 |
| 603 | MDN1 | 2 | 0.1752 | 0.0949 | 0.1752 | 0.0949 |
| 604 | MECP2 | 2 | -0.0955 | 0.3653 | -0.0955 | 0.3653 |
| 605 | MED1 | 2 | -0.1068 | 0.3110 | -0.1068 | 0.3110 |
| 606 | MED13 | 4 | 0.5147 | 0.9569 | 0.0057 | 0.0000 |
| 607 | MED26 | 4 | 0.2194 | 0.9787 | -0.1883 | 0.0356 |
| 608 | MEGF8 | 2 | 0.0529 | 0.6166 | 0.0529 | 0.6166 |
| 609 | MEN1 | 2 | 0.3522 | 0.0006 | 0.3522 | 0.0006 |
| 610 | MESD | 2 | 0.0663 | 0.5298 | 0.0663 | 0.5298 |
| 611 | METTL16 | 2 | -0.1030 | 0.3285 | -0.1030 | 0.3285 |
| 612 | METTL18 | 2 | 0.1576 | 0.1335 | 0.1576 | 0.1335 |
| 613 | MEX3A | 2 | 0.0132 | 0.9009 | 0.0132 | 0.9009 |
| 614 | MFHAS1 | 5 | 0.0917 | 0.9704 | -0.2889 | 0.0052 |
| 615 | MGA | 2 | 0.0471 | 0.6558 | 0.0471 | 0.6558 |
| 616 | MICAL3 | 4 | 0.1685 | 0.8127 | -0.1252 | 0.1083 |
| 617 | MID1 | 2 | -0.3439 | 0.0008 | -0.3439 | 0.0008 |
| 618 | MID1IP1 | 2 | -0.0276 | 0.7942 | -0.0276 | 0.7942 |
| 619 | MKI67 | 10 | 0.5128 | 0.9716 | -0.3255 | 0.0000 |
| 620 | MLLT1 | 4 | 0.1048 | 0.9206 | -0.1279 | 0.2244 |
| 621 | MLLT3 | 2 | 0.0585 | 0.5794 | 0.0585 | 0.5794 |
| 622 | MLXIP | 7 | 0.3543 | 0.9448 | -0.2127 | 0.0005 |
| 623 | MNX1 | 3 | 0.3725 | 0.7209 | -0.0377 | 0.0003 |
| 624 | MOGS | 3 | 0.1955 | 0.2432 | -0.1229 | 0.0618 |
| 625 | MPHOSPH10 | 2 | 0.2544 | 0.0144 | 0.2544 | 0.0144 |
| 626 | MPRIP | 2 | -0.1022 | 0.3324 | -0.1022 | 0.3324 |
| 627 | MRFAP1 | 2 | 0.3048 | 0.0031 | 0.3048 | 0.0031 |
| 628 | MRFAP1L1 | 3 | 0.0913 | 0.3867 | -0.3097 | 0.0027 |
| 629 | MRGBP | 2 | -0.2841 | 0.0061 | -0.2841 | 0.0061 |
| 630 | MRM2 | 2 | -0.1733 | 0.0986 | -0.1733 | 0.0986 |
| 631 | MRM3 | 3 | -0.1411 | 0.1797 | -0.1914 | 0.0676 |
| 632 | MRPS21 | 2 | -0.0344 | 0.7448 | -0.0344 | 0.7448 |
| 633 | MRTFA | 2 | -0.0954 | 0.3655 | -0.0954 | 0.3655 |
| 634 | MSL1 | 4 | 0.2030 | 0.2690 | -0.2093 | 0.0453 |
| 635 | MSRB1 | 4 | 0.3440 | 0.5361 | -0.3077 | 0.0008 |
| 636 | MTCH1 | 2 | 0.2073 | 0.0474 | 0.2073 | 0.0474 |
| 637 | MTCL1 | 6 | 0.4454 | 0.9106 | -0.3063 | 0.0000 |
| 638 | MTERF4 | 2 | 0.4201 | 0.0000 | 0.4201 | 0.0000 |
| 639 | MTG1 | 2 | 0.1271 | 0.2273 | 0.1271 | 0.2273 |
| 640 | MTG2 | 3 | 0.5120 | 0.7015 | -0.1979 | 0.0000 |
| 641 | MTHFSD | 3 | -0.0606 | 0.5658 | -0.0937 | 0.3745 |
| 642 | MTMR3 | 3 | 0.2816 | 0.4446 | -0.2482 | 0.0065 |
| 643 | MTMR4 | 3 | 0.3887 | 0.1595 | -0.1857 | 0.0001 |
| 644 | MTSS1L | 2 | 0.6705 | 0.0000 | 0.6705 | 0.0000 |
| 645 | MUL1 | 3 | 0.1886 | 0.9525 | -0.0715 | 0.0718 |
| 646 | MVB12B | 2 | 0.3030 | 0.0033 | 0.3030 | 0.0033 |
| 647 | MYC | 4 | 0.3364 | 0.9410 | -0.1293 | 0.0010 |
| 648 | MYCBP2 | 2 | -0.1542 | 0.1422 | -0.1542 | 0.1422 |
| 649 | MYH9 | 3 | 0.3496 | 0.3843 | -0.1552 | 0.0006 |
| 650 | MYO10 | 3 | 0.2991 | 0.5046 | 0.0705 | 0.0038 |
| 651 | MYO9A | 2 | -0.0179 | 0.8653 | -0.0179 | 0.8653 |
| 652 | MYO9B | 6 | 0.5035 | 0.9852 | -0.2268 | 0.0000 |
| 653 | MZF1 | 2 | 0.1461 | 0.1646 | 0.1461 | 0.1646 |
| 654 | N4BP2L2 | 2 | -0.1273 | 0.2267 | -0.1273 | 0.2267 |
| 655 | NAA40 | 2 | 0.1374 | 0.1914 | 0.1374 | 0.1914 |
| 656 | NAB2 | 2 | 0.1264 | 0.2300 | 0.1264 | 0.2300 |
| 657 | NAF1 | 2 | 0.1364 | 0.1947 | 0.1364 | 0.1947 |
| 658 | NAGLU | 2 | 0.0338 | 0.7493 | 0.0338 | 0.7493 |
| 659 | NATD1 | 2 | 0.0746 | 0.4800 | 0.0746 | 0.4800 |
| 660 | NAV1 | 2 | 0.0876 | 0.4062 | 0.0876 | 0.4062 |
| 661 | NAV2 | 2 | 0.0368 | 0.7276 | 0.0368 | 0.7276 |
| 662 | NCALD | 2 | 0.0506 | 0.6318 | 0.0506 | 0.6318 |
| 663 | NCAPH2 | 2 | 0.1969 | 0.0599 | 0.1969 | 0.0599 |
| 664 | NCKAP5L | 4 | 0.7993 | 0.0976 | 0.1738 | 0.0000 |
| 665 | NCOA2 | 2 | -0.1137 | 0.2804 | -0.1137 | 0.2804 |
| 666 | NCOR2 | 3 | 0.3542 | 0.3879 | 0.0911 | 0.0005 |
| 667 | NDST1 | 2 | 0.3155 | 0.0022 | 0.3155 | 0.0022 |
| 668 | NDST2 | 2 | 0.2179 | 0.0369 | 0.2179 | 0.0369 |
| 669 | NEAT1 | 3 | 0.3284 | 0.4056 | 0.0877 | 0.0014 |
| 670 | NECAP2 | 2 | -0.0371 | 0.7252 | -0.0371 | 0.7252 |
| 671 | NEDD9 | 2 | -0.3064 | 0.0030 | -0.3064 | 0.0030 |
| 672 | NETO2 | 3 | 0.2673 | 0.9023 | -0.0867 | 0.0100 |
| 673 | NEU1 | 4 | 0.0669 | 0.8711 | -0.2927 | 0.0046 |
| 674 | NFE2L1 | 3 | 0.2130 | 0.4389 | 0.0817 | 0.0415 |
| 675 | NFE2L2 | 2 | 0.0834 | 0.4295 | 0.0834 | 0.4295 |
| 676 | NFIC | 3 | 0.3533 | 0.3446 | -0.0997 | 0.0006 |
| 677 | NFKBIE | 2 | -0.0469 | 0.6573 | -0.0469 | 0.6573 |
| 678 | NFX1 | 2 | -0.0718 | 0.4967 | -0.0718 | 0.4967 |
| 679 | NFYA | 3 | 0.3105 | 0.9712 | 0.0038 | 0.0026 |
| 680 | NIF3L1 | 2 | 0.0628 | 0.5518 | 0.0628 | 0.5518 |
| 681 | NINJ1 | 2 | -0.4913 | 0.0000 | -0.4913 | 0.0000 |
| 682 | NINL | 2 | -0.0646 | 0.5405 | -0.0646 | 0.5405 |
| 683 | NKX3 | 2 | -0.3791 | 0.0002 | -0.3791 | 0.0002 |
| 684 | NLGN2 | 4 | 0.8379 | 0.7126 | -0.1308 | 0.0000 |
| 685 | NMRAL1 | 2 | 0.0184 | 0.8620 | 0.0184 | 0.8620 |
| 686 | NMT1 | 2 | -0.1231 | 0.2424 | -0.1231 | 0.2424 |
| 687 | NOL6 | 3 | 0.3121 | 0.2522 | 0.1206 | 0.0025 |
| 688 | NPLOC4 | 4 | 0.0984 | 0.4912 | -0.2700 | 0.0093 |
| 689 | NR2F2 | 2 | 0.3714 | 0.0003 | 0.3714 | 0.0003 |
| 690 | NR2F6 | 2 | 0.1824 | 0.0818 | 0.1824 | 0.0818 |
| 691 | NRIP1 | 4 | 0.3194 | 0.8515 | -0.1222 | 0.0019 |
| 692 | NSD1 | 2 | -0.0477 | 0.6514 | -0.0477 | 0.6514 |
| 693 | NUAK1 | 2 | 0.0930 | 0.3781 | 0.0930 | 0.3781 |
| 694 | NUAK2 | 2 | -0.1804 | 0.0853 | -0.1804 | 0.0853 |
| 695 | NUFIP2 | 6 | 0.1845 | 0.7641 | -0.3495 | 0.0006 |
| 696 | NUP153 | 2 | 0.0079 | 0.9405 | 0.0079 | 0.9405 |
| 697 | NUP210 | 3 | 0.2546 | 0.4443 | -0.0807 | 0.0143 |
| 698 | NXN | 2 | -0.1702 | 0.1049 | -0.1702 | 0.1049 |
| 699 | NXT1 | 2 | 0.2820 | 0.0065 | 0.2820 | 0.0065 |
| 700 | OGA | 2 | -0.1946 | 0.0631 | -0.1946 | 0.0631 |
| 701 | OGFR | 3 | 0.0761 | 0.4709 | -0.1752 | 0.0947 |
| 702 | OGT | 3 | 0.2840 | 0.7883 | 0.0284 | 0.0061 |
| 703 | OLFM1 | 3 | 0.1419 | 0.3300 | -0.2550 | 0.0142 |
| 704 | OSBPL2 | 2 | -0.0937 | 0.3744 | -0.0937 | 0.3744 |
| 705 | OTUD7B | 5 | 0.2375 | 0.2768 | -0.4914 | 0.0000 |
| 706 | OXLD1 | 4 | 0.4700 | 0.6593 | -0.0466 | 0.0000 |
| 707 | P4HB | 2 | 0.0294 | 0.7811 | 0.0294 | 0.7811 |
| 708 | PAGR1 | 2 | 0.0596 | 0.5724 | 0.0596 | 0.5724 |
| 709 | PALB2 | 4 | 0.3167 | 0.4510 | -0.1871 | 0.0021 |
| 710 | PALM | 2 | 0.1241 | 0.2385 | 0.1241 | 0.2385 |
| 711 | PAPPA | 3 | 0.3218 | 0.8095 | -0.2783 | 0.0018 |
| 712 | PAQR4 | 2 | 0.0120 | 0.9100 | 0.0120 | 0.9100 |
| 713 | PARP1 | 2 | -0.3629 | 0.0004 | -0.3629 | 0.0004 |
| 714 | PARP10 | 2 | 0.1069 | 0.3103 | 0.1069 | 0.3103 |
| 715 | PARP9 | 2 | -0.0725 | 0.4921 | -0.0725 | 0.4921 |
| 716 | PASK | 3 | 0.0777 | 0.4615 | -0.1163 | 0.2697 |
| 717 | PBXIP1 | 2 | -0.0600 | 0.5702 | -0.0600 | 0.5702 |
| 718 | PCED1A | 2 | 0.2787 | 0.0071 | 0.2787 | 0.0071 |
| 719 | PCF11 | 2 | -0.0265 | 0.8022 | -0.0265 | 0.8022 |
| 720 | PCGF3 | 2 | 0.2906 | 0.0050 | 0.2906 | 0.0050 |
| 721 | PCK2 | 2 | 0.0701 | 0.5064 | 0.0701 | 0.5064 |
| 722 | PCMTD2 | 2 | 0.0160 | 0.8794 | 0.0160 | 0.8794 |
| 723 | PCNX3 | 3 | 0.3358 | 0.5313 | -0.1190 | 0.0011 |
| 724 | PCYT2 | 2 | 0.5313 | 0.0000 | 0.5313 | 0.0000 |
| 725 | PDCD11 | 2 | -0.3273 | 0.0014 | -0.3273 | 0.0014 |
| 726 | PDE4B | 2 | -0.0834 | 0.4292 | -0.0834 | 0.4292 |
| 727 | PDE4D | 2 | -0.5333 | 0.0000 | -0.5333 | 0.0000 |
| 728 | PDE8A | 3 | 0.1075 | 0.5763 | -0.0791 | 0.3078 |
| 729 | PDP2 | 4 | 0.2061 | 0.5501 | -0.2409 | 0.0207 |
| 730 | PDPR | 2 | -0.2426 | 0.0198 | -0.2426 | 0.0198 |
| 731 | PDZD8 | 2 | -0.0851 | 0.4201 | -0.0851 | 0.4201 |
| 732 | PEAK1 | 3 | 0.0820 | 0.8954 | -0.1565 | 0.1363 |
| 733 | PELO | 2 | 0.0367 | 0.7287 | 0.0367 | 0.7287 |
| 734 | PEX10 | 2 | 0.1172 | 0.2659 | 0.1172 | 0.2659 |
| 735 | PFKFB3 | 5 | 0.3989 | 0.3268 | -0.3209 | 0.0001 |
| 736 | PFKL | 2 | 0.0105 | 0.9208 | 0.0105 | 0.9208 |
| 737 | PFKM | 2 | -0.1010 | 0.3381 | -0.1010 | 0.3381 |
| 738 | PGAM5 | 2 | -0.1388 | 0.1870 | -0.1388 | 0.1870 |
| 739 | PGBD5 | 2 | -0.1876 | 0.0733 | -0.1876 | 0.0733 |
| 740 | PGGHG | 2 | 0.2255 | 0.0306 | 0.2255 | 0.0306 |
| 741 | PGPEP1 | 2 | -0.0972 | 0.3569 | -0.0972 | 0.3569 |
| 742 | PHACTR2 | 3 | 0.4062 | 0.8955 | 0.0139 | 0.0001 |
| 743 | PHB2 | 2 | 0.0226 | 0.8309 | 0.0226 | 0.8309 |
| 744 | PHF12 | 3 | 0.3500 | 0.1477 | 0.1521 | 0.0006 |
| 745 | PHF13 | 2 | -0.1065 | 0.3122 | -0.1065 | 0.3122 |
| 746 | PHF20 | 3 | 0.1931 | 0.8581 | -0.0821 | 0.0652 |
| 747 | PHF3 | 2 | 0.0414 | 0.6951 | 0.0414 | 0.6951 |
| 748 | PHF8 | 2 | 0.2906 | 0.0050 | 0.2906 | 0.0050 |
| 749 | PHLDA1 | 3 | 0.4420 | 0.4989 | -0.0714 | 0.0000 |
| 750 | PHLDB1 | 5 | 0.4844 | 0.3703 | -0.3072 | 0.0000 |
| 751 | PHLDB2 | 3 | 0.1676 | 0.2555 | -0.2337 | 0.0249 |
| 752 | PHLPP2 | 3 | 0.2977 | 0.7950 | -0.1793 | 0.0039 |
| 753 | PHRF1 | 2 | 0.1909 | 0.0683 | 0.1909 | 0.0683 |
| 754 | PHYKPL | 2 | -0.0555 | 0.5991 | -0.0555 | 0.5991 |
| 755 | PI4KB | 2 | 0.0955 | 0.3654 | 0.0955 | 0.3654 |
| 756 | PIGC | 3 | 0.2834 | 0.3799 | -0.0926 | 0.0062 |
| 757 | PIGG | 2 | -0.0602 | 0.5687 | -0.0602 | 0.5687 |
| 758 | PIGQ | 3 | 0.0663 | 0.8534 | -0.0715 | 0.4980 |
| 759 | PIK3CG | 2 | 0.2769 | 0.0075 | 0.2769 | 0.0075 |
| 760 | PINK1.AS | 4 | 0.3787 | 0.1415 | 0.1545 | 0.0002 |
| 761 | PIP5K1C | 2 | 0.1831 | 0.0807 | 0.1831 | 0.0807 |
| 762 | PKM | 4 | 0.3158 | 0.8684 | 0.0175 | 0.0022 |
| 763 | PLCD3 | 2 | 0.3014 | 0.0035 | 0.3014 | 0.0035 |
| 764 | PLEC | 6 | 0.6470 | 0.6782 | -0.2843 | 0.0000 |
| 765 | PLEKHA6 | 2 | 0.2467 | 0.0178 | 0.2467 | 0.0178 |
| 766 | PLEKHA7 | 2 | -0.3451 | 0.0008 | -0.3451 | 0.0008 |
| 767 | PLEKHF1 | 2 | -0.1875 | 0.0734 | -0.1875 | 0.0734 |
| 768 | PLEKHG2 | 5 | 0.6288 | 0.8656 | -0.5339 | 0.0000 |
| 769 | PLEKHG3 | 4 | 0.1263 | 0.6728 | -0.3117 | 0.0025 |
| 770 | PLEKHH3 | 2 | 0.0722 | 0.4942 | 0.0722 | 0.4942 |
| 771 | PLEKHM2 | 2 | -0.0969 | 0.3583 | -0.0969 | 0.3583 |
| 772 | PLOD1 | 5 | 0.2885 | 0.8860 | -0.3626 | 0.0004 |
| 773 | PLXNA1 | 2 | 0.0224 | 0.8325 | 0.0224 | 0.8325 |
| 774 | PLXNB2 | 2 | -0.1186 | 0.2600 | -0.1186 | 0.2600 |
| 775 | PMAIP1 | 2 | 0.2999 | 0.0037 | 0.2999 | 0.0037 |
| 776 | PML | 2 | 0.1086 | 0.3028 | 0.1086 | 0.3028 |
| 777 | PMM2 | 2 | -0.2101 | 0.0444 | -0.2101 | 0.0444 |
| 778 | POGZ | 3 | 0.2899 | 0.9819 | -0.0985 | 0.0051 |
| 779 | POLDIP2 | 2 | -0.2249 | 0.0311 | -0.2249 | 0.0311 |
| 780 | POMGNT1 | 2 | 0.0551 | 0.6018 | 0.0551 | 0.6018 |
| 781 | POU2F2 | 3 | 0.3417 | 0.0216 | -0.2754 | 0.0009 |
| 782 | PP7080 | 4 | 0.2071 | 0.4980 | -0.0715 | 0.0477 |
| 783 | PPIF | 2 | 0.0817 | 0.4387 | 0.0817 | 0.4387 |
| 784 | PPIL2 | 4 | 0.1311 | 0.7905 | -0.1021 | 0.2128 |
| 785 | PPP1R10 | 3 | 0.2703 | 0.8946 | -0.0191 | 0.0092 |
| 786 | PPP1R13B | 2 | -0.1581 | 0.1323 | -0.1581 | 0.1323 |
| 787 | PPP1R15A | 2 | 0.1855 | 0.0766 | 0.1855 | 0.0766 |
| 788 | PPP1R15B | 2 | -0.1613 | 0.1246 | -0.1613 | 0.1246 |
| 789 | PPP1R26 | 4 | 0.5129 | 0.1340 | -0.2634 | 0.0000 |
| 790 | PPP1R8 | 2 | 0.2995 | 0.0037 | 0.2995 | 0.0037 |
| 791 | PPP2CA | 2 | 0.1839 | 0.0792 | 0.1839 | 0.0792 |
| 792 | PPP2R2D | 2 | -0.0393 | 0.7098 | -0.0393 | 0.7098 |
| 793 | PPRC1 | 5 | 0.1280 | 0.8197 | -0.4430 | 0.0000 |
| 794 | PQLC2 | 2 | 0.2747 | 0.0081 | 0.2747 | 0.0081 |
| 795 | PRAF2 | 3 | 0.2977 | 0.0230 | 0.2369 | 0.0040 |
| 796 | PRAG1 | 6 | 0.5055 | 0.5277 | -0.3090 | 0.0000 |
| 797 | PRICKLE4 | 3 | 0.4218 | 0.2470 | -0.2933 | 0.0000 |
| 798 | PRMT6 | 2 | -0.0604 | 0.5672 | -0.0604 | 0.5672 |
| 799 | PRPF38A | 2 | -0.1689 | 0.1075 | -0.1689 | 0.1075 |
| 800 | PRPF4 | 3 | 0.4632 | 0.0353 | 0.2197 | 0.0000 |
| 801 | PRPSAP1 | 2 | 0.1701 | 0.1050 | 0.1701 | 0.1050 |
| 802 | PRR14L | 2 | 0.1381 | 0.1894 | 0.1381 | 0.1894 |
| 803 | PRRC2A | 4 | 0.6568 | 0.4431 | 0.0809 | 0.0000 |
| 804 | PRRC2B | 4 | 0.2297 | 0.7573 | -0.2130 | 0.0276 |
| 805 | PRRC2C | 6 | 0.4392 | 0.9082 | -0.2186 | 0.0000 |
| 806 | PRSS23 | 2 | -0.2158 | 0.0389 | -0.2158 | 0.0389 |
| 807 | PRUNE1 | 2 | 0.1409 | 0.1805 | 0.1409 | 0.1805 |
| 808 | PSD4 | 2 | -0.1594 | 0.1292 | -0.1594 | 0.1292 |
| 809 | PSMD5 | 2 | 0.0510 | 0.6291 | 0.0510 | 0.6291 |
| 810 | PSRC1 | 2 | -0.1428 | 0.1746 | -0.1428 | 0.1746 |
| 811 | PTPN12 | 2 | -0.1200 | 0.2544 | -0.1200 | 0.2544 |
| 812 | PTPRS | 2 | 0.2920 | 0.0047 | 0.2920 | 0.0047 |
| 813 | PUM1 | 2 | 0.4085 | 0.0001 | 0.4085 | 0.0001 |
| 814 | PWWP2B | 4 | 0.4148 | 0.8214 | -0.0645 | 0.0000 |
| 815 | PXDN | 3 | 0.3197 | 0.9486 | -0.2311 | 0.0019 |
| 816 | PXMP2 | 2 | -0.1307 | 0.2143 | -0.1307 | 0.2143 |
| 817 | PYCR1 | 3 | 0.4450 | 0.0593 | -0.2410 | 0.0000 |
| 818 | PYCR3 | 2 | 0.0496 | 0.6385 | 0.0496 | 0.6385 |
| 819 | PYGO2 | 2 | 0.1670 | 0.1116 | 0.1670 | 0.1116 |
| 820 | PYM1 | 3 | 0.5144 | 0.0192 | 0.2439 | 0.0000 |
| 821 | QPCTL | 2 | -0.0140 | 0.8950 | -0.0140 | 0.8950 |
| 822 | QRICH1 | 2 | 0.3740 | 0.0002 | 0.3740 | 0.0002 |
| 823 | QSOX1 | 2 | 0.2109 | 0.0436 | 0.2109 | 0.0436 |
| 824 | QSOX2 | 2 | -0.1542 | 0.1423 | -0.1542 | 0.1423 |
| 825 | RAB11FIP1 | 2 | -0.1385 | 0.1879 | -0.1385 | 0.1879 |
| 826 | RAB35 | 2 | -0.0332 | 0.7535 | -0.0332 | 0.7535 |
| 827 | RAB40C | 2 | -0.2779 | 0.0073 | -0.2779 | 0.0073 |
| 828 | RAB8A | 2 | -0.0538 | 0.6109 | -0.0538 | 0.6109 |
| 829 | RACK1 | 2 | 0.5872 | 0.0000 | 0.5872 | 0.0000 |
| 830 | RAD51D | 2 | -0.1318 | 0.2104 | -0.1318 | 0.2104 |
| 831 | RAD54L2 | 2 | -0.0029 | 0.9780 | -0.0029 | 0.9780 |
| 832 | RAI1 | 8 | 0.5476 | 0.8745 | -0.3517 | 0.0000 |
| 833 | RANBP10 | 2 | 0.2863 | 0.0057 | 0.2863 | 0.0057 |
| 834 | RAP1GAP | 2 | 0.0708 | 0.5027 | 0.0708 | 0.5027 |
| 835 | RAPH1 | 2 | -0.0871 | 0.4091 | -0.0871 | 0.4091 |
| 836 | RARA | 3 | 0.4042 | 0.0571 | -0.4966 | 0.0000 |
| 837 | RASSF2 | 4 | 0.1927 | 0.8363 | -0.0877 | 0.0657 |
| 838 | RASSF8 | 2 | -0.0149 | 0.8880 | -0.0149 | 0.8880 |
| 839 | RBBP6 | 2 | 0.1576 | 0.1335 | 0.1576 | 0.1335 |
| 840 | RBFA | 3 | 0.1211 | 0.7205 | -0.1688 | 0.1078 |
| 841 | RBM12 | 2 | 0.1560 | 0.1376 | 0.1560 | 0.1376 |
| 842 | RBM12B | 2 | 0.2546 | 0.0143 | 0.2546 | 0.0143 |
| 843 | RBM15 | 5 | 0.4466 | 0.2403 | -0.4586 | 0.0000 |
| 844 | RBM15B | 9 | 0.4656 | 0.9894 | -0.3434 | 0.0000 |
| 845 | RBM22 | 2 | 0.0701 | 0.5068 | 0.0701 | 0.5068 |
| 846 | RBM48 | 2 | -0.0577 | 0.5847 | -0.0577 | 0.5847 |
| 847 | RBM6 | 3 | 0.0784 | 0.8810 | 0.0158 | 0.4575 |
| 848 | RBMXL1 | 2 | -0.4076 | 0.0001 | -0.4076 | 0.0001 |
| 849 | RBSN | 3 | 0.3073 | 0.0714 | 0.1889 | 0.0029 |
| 850 | RCC1 | 2 | 0.0388 | 0.7135 | 0.0388 | 0.7135 |
| 851 | RCOR1 | 2 | 0.2977 | 0.0039 | 0.2977 | 0.0039 |
| 852 | RELA | 2 | 0.0258 | 0.8068 | 0.0258 | 0.8068 |
| 853 | REPIN1 | 3 | 0.1514 | 0.2727 | -0.1436 | 0.1497 |
| 854 | RERE | 4 | 0.5404 | 0.9580 | -0.1295 | 0.0000 |
| 855 | RETREG2 | 2 | -0.0011 | 0.9919 | -0.0011 | 0.9919 |
| 856 | RETREG3 | 2 | 0.0726 | 0.4914 | 0.0726 | 0.4914 |
| 857 | REV1 | 2 | -0.0972 | 0.3568 | -0.0972 | 0.3568 |
| 858 | RFX7 | 3 | 0.0980 | 0.9367 | -0.0679 | 0.3526 |
| 859 | RGMB | 3 | -0.0393 | 0.7097 | -0.0718 | 0.4962 |
| 860 | RHNO1 | 3 | 0.1672 | 0.4572 | -0.1536 | 0.1112 |
| 861 | RHOA | 2 | 0.2182 | 0.0367 | 0.2182 | 0.0367 |
| 862 | RHOB | 4 | 0.2615 | 0.4569 | -0.1271 | 0.0118 |
| 863 | RHOBTB1 | 3 | 0.1343 | 0.7973 | -0.1522 | 0.1476 |
| 864 | RHOF | 2 | 0.2809 | 0.0067 | 0.2809 | 0.0067 |
| 865 | RIC1 | 2 | 0.3372 | 0.0010 | 0.3372 | 0.0010 |
| 866 | RIC8A | 3 | 0.2029 | 0.9461 | -0.1019 | 0.0524 |
| 867 | RIC8B | 2 | -0.0126 | 0.9055 | -0.0126 | 0.9055 |
| 868 | RILPL2 | 3 | 0.6252 | 0.7978 | -0.0271 | 0.0000 |
| 869 | RIN3 | 2 | 0.2497 | 0.0164 | 0.2497 | 0.0164 |
| 870 | RIPK1 | 2 | 0.1338 | 0.2036 | 0.1338 | 0.2036 |
| 871 | RNF111 | 2 | -0.0684 | 0.5169 | -0.0684 | 0.5169 |
| 872 | RNF135 | 2 | -0.0569 | 0.5901 | -0.0569 | 0.5901 |
| 873 | RNF168 | 3 | 0.0343 | 0.7457 | -0.1080 | 0.3054 |
| 874 | RNF19A | 2 | -0.1129 | 0.2837 | -0.1129 | 0.2837 |
| 875 | RNF19B | 2 | -0.0714 | 0.4990 | -0.0714 | 0.4990 |
| 876 | RNF20 | 2 | -0.0158 | 0.8809 | -0.0158 | 0.8809 |
| 877 | RNF217 | 2 | 0.0455 | 0.6667 | 0.0455 | 0.6667 |
| 878 | RNF25 | 2 | 0.1496 | 0.1545 | 0.1496 | 0.1545 |
| 879 | RNF26 | 3 | -0.0015 | 0.9884 | -0.2404 | 0.0210 |
| 880 | RNF34 | 2 | 0.2433 | 0.0195 | 0.2433 | 0.0195 |
| 881 | RNF43 | 2 | -0.1251 | 0.2349 | -0.1251 | 0.2349 |
| 882 | RPL13 | 2 | 0.0910 | 0.3885 | 0.0910 | 0.3885 |
| 883 | RPP25 | 3 | 0.0849 | 0.8307 | -0.0226 | 0.4208 |
| 884 | RPP25L | 2 | -0.2310 | 0.0267 | -0.2310 | 0.0267 |
| 885 | RPRD2 | 3 | 0.0321 | 0.8344 | -0.0318 | 0.7612 |
| 886 | RPUSD2 | 3 | 0.3411 | 0.7638 | -0.0318 | 0.0009 |
| 887 | RREB1 | 4 | 0.7364 | 0.0032 | -0.3740 | 0.0000 |
| 888 | RRP1B | 2 | 0.1193 | 0.2574 | 0.1193 | 0.2574 |
| 889 | RRP8 | 2 | 0.1735 | 0.0982 | 0.1735 | 0.0982 |
| 890 | RRS1 | 3 | 0.0627 | 0.6745 | -0.2858 | 0.0058 |
| 891 | RSF1 | 3 | 0.1360 | 0.3876 | 0.0911 | 0.1961 |
| 892 | RSPRY1 | 2 | -0.3365 | 0.0010 | -0.3365 | 0.0010 |
| 893 | RTKN2 | 2 | -0.0490 | 0.6426 | -0.0490 | 0.6426 |
| 894 | RTL10 | 2 | -0.2063 | 0.0485 | -0.2063 | 0.0485 |
| 895 | RTL6 | 5 | 0.1768 | 0.9807 | -0.2589 | 0.0127 |
| 896 | RUSC2 | 3 | 0.3585 | 0.4817 | 0.0743 | 0.0004 |
| 897 | RXRA | 2 | 0.0754 | 0.4749 | 0.0754 | 0.4749 |
| 898 | S1PR3 | 2 | 0.5120 | 0.0000 | 0.5120 | 0.0000 |
| 899 | SACS | 4 | 0.1853 | 0.6618 | -0.3613 | 0.0004 |
| 900 | SAP30BP | 2 | 0.1154 | 0.2735 | 0.1154 | 0.2735 |
| 901 | SART3 | 4 | 0.2573 | 0.6575 | -0.1681 | 0.0133 |
| 902 | SASH1 | 3 | 0.2162 | 0.1386 | 0.1556 | 0.0384 |
| 903 | SCAF11 | 3 | 0.4459 | 0.2701 | -0.1466 | 0.0000 |
| 904 | SCAF8 | 2 | -0.2384 | 0.0221 | -0.2384 | 0.0221 |
| 905 | SCARA3 | 4 | 0.5610 | 0.3865 | 0.0913 | 0.0000 |
| 906 | SCARB1 | 2 | 0.2242 | 0.0317 | 0.2242 | 0.0317 |
| 907 | SCD | 2 | 0.2314 | 0.0264 | 0.2314 | 0.0264 |
| 908 | SCFD2 | 2 | -0.2624 | 0.0115 | -0.2624 | 0.0115 |
| 909 | SDC1 | 2 | 0.0888 | 0.3998 | 0.0888 | 0.3998 |
| 910 | SDHAF1 | 5 | 0.4598 | 0.1571 | -0.5438 | 0.0000 |
| 911 | SEC16A | 7 | 0.1511 | 0.9665 | -0.3493 | 0.0006 |
| 912 | SEC61A1 | 4 | 0.3723 | 0.7927 | -0.5190 | 0.0000 |
| 913 | SEMA3C | 2 | 0.0095 | 0.9282 | 0.0095 | 0.9282 |
| 914 | SEMA4B | 3 | 0.2164 | 0.8358 | -0.2341 | 0.0247 |
| 915 | SEMA4D | 5 | 0.2324 | 0.9968 | -0.1714 | 0.0258 |
| 916 | SEPHS2 | 2 | 0.1789 | 0.0879 | 0.1789 | 0.0879 |
| 917 | 9-Sep | 5 | 0.3532 | 0.9434 | -0.3168 | 0.0006 |
| 918 | SERPINH1 | 5 | 0.3450 | 0.9857 | -0.1190 | 0.0008 |
| 919 | SERTAD2 | 2 | 0.5306 | 0.0000 | 0.5306 | 0.0000 |
| 920 | SETD1A | 2 | -0.1360 | 0.1960 | -0.1360 | 0.1960 |
| 921 | SETD1B | 2 | 0.0838 | 0.4272 | 0.0838 | 0.4272 |
| 922 | SETD2 | 2 | -0.2688 | 0.0096 | -0.2688 | 0.0096 |
| 923 | SETD5 | 3 | 0.3788 | 0.3977 | 0.0892 | 0.0002 |
| 924 | SETDB1 | 2 | -0.0131 | 0.9016 | -0.0131 | 0.9016 |
| 925 | SF1 | 2 | 0.6396 | 0.0000 | 0.6396 | 0.0000 |
| 926 | SGMS1 | 5 | 0.1198 | 0.9263 | -0.2515 | 0.0156 |
| 927 | SH3BP2 | 2 | 0.4559 | 0.0000 | 0.4559 | 0.0000 |
| 928 | SH3BP4 | 5 | 0.3023 | 0.9277 | -0.0685 | 0.0034 |
| 929 | SH3BP5L | 2 | -0.0661 | 0.5312 | -0.0661 | 0.5312 |
| 930 | SH3D19 | 2 | -0.3140 | 0.0023 | -0.3140 | 0.0023 |
| 931 | SH3PXD2A | 6 | 0.5622 | 0.5888 | -0.2164 | 0.0000 |
| 932 | SH3PXD2B | 5 | 0.6480 | 0.8941 | -0.3295 | 0.0000 |
| 933 | SHPK | 3 | 0.2122 | 0.6918 | -0.1441 | 0.0423 |
| 934 | SHROOM3 | 2 | -0.1983 | 0.0581 | -0.1983 | 0.0581 |
| 935 | SIK2 | 2 | 0.0890 | 0.3987 | 0.0890 | 0.3987 |
| 936 | SIL1 | 3 | 0.1347 | 0.9779 | -0.0029 | 0.2005 |
| 937 | SIPA1L3 | 4 | 0.3916 | 0.7808 | -0.3057 | 0.0001 |
| 938 | SIRPA | 2 | 0.5110 | 0.0000 | 0.5110 | 0.0000 |
| 939 | SKI | 2 | -0.1188 | 0.2595 | -0.1188 | 0.2595 |
| 940 | SKIL | 2 | -0.1650 | 0.1160 | -0.1650 | 0.1160 |
| 941 | SLAMF7 | 2 | -0.0622 | 0.5559 | -0.0622 | 0.5559 |
| 942 | SLC10A3 | 4 | 0.1425 | 0.5774 | -0.1407 | 0.1754 |
| 943 | SLC12A7 | 2 | -0.3076 | 0.0029 | -0.3076 | 0.0029 |
| 944 | SLC16A1 | 4 | 0.0108 | 0.9429 | -0.2138 | 0.0407 |
| 945 | SLC16A5 | 2 | 0.2499 | 0.0163 | 0.2499 | 0.0163 |
| 946 | SLC19A1 | 3 | -0.0090 | 0.9322 | -0.2399 | 0.0213 |
| 947 | SLC1A5 | 6 | 0.2181 | 0.9589 | -0.2215 | 0.0339 |
| 948 | SLC22A23 | 3 | 0.1562 | 0.8723 | -0.0250 | 0.1370 |
| 949 | SLC23A2 | 2 | 0.0451 | 0.6693 | 0.0451 | 0.6693 |
| 950 | SLC25A25.AS1 | 2 | -0.0489 | 0.6434 | -0.0489 | 0.6434 |
| 951 | SLC25A29 | 4 | 0.4055 | 0.9923 | -0.0010 | 0.0001 |
| 952 | SLC25A37 | 3 | 0.1140 | 0.4449 | 0.0806 | 0.2793 |
| 953 | SLC25A44 | 3 | 0.0345 | 0.7438 | -0.1122 | 0.2869 |
| 954 | SLC27A4 | 2 | 0.3272 | 0.0015 | 0.3272 | 0.0015 |
| 955 | SLC33A1 | 2 | 0.0453 | 0.6679 | 0.0453 | 0.6679 |
| 956 | SLC35A4 | 2 | -0.2568 | 0.0135 | -0.2568 | 0.0135 |
| 957 | SLC38A10 | 3 | 0.0585 | 0.5796 | -0.1996 | 0.0564 |
| 958 | SLC39A3 | 2 | -0.0617 | 0.5594 | -0.0617 | 0.5594 |
| 959 | SLC39A6 | 2 | 0.2021 | 0.0534 | 0.2021 | 0.0534 |
| 960 | SLC3A2 | 2 | -0.3259 | 0.0015 | -0.3259 | 0.0015 |
| 961 | SLC45A4 | 3 | 0.1104 | 0.2948 | -0.2260 | 0.0303 |
| 962 | SLC46A1 | 2 | -0.0144 | 0.8919 | -0.0144 | 0.8919 |
| 963 | SLC4A2 | 2 | -0.2061 | 0.0487 | -0.2061 | 0.0487 |
| 964 | SLC7A1 | 3 | 0.5797 | 0.0000 | 0.4121 | 0.0000 |
| 965 | SLC7A5 | 5 | 0.4509 | 0.9111 | -0.0705 | 0.0000 |
| 966 | SLC9A3.AS1 | 3 | 0.4506 | 0.7490 | -0.1717 | 0.0000 |
| 967 | SLX4 | 4 | 0.4303 | 0.5938 | -0.2718 | 0.0000 |
| 968 | SMAD3 | 4 | 0.5713 | 0.8728 | -0.0351 | 0.0000 |
| 969 | SMCR8 | 5 | 0.4373 | 0.9241 | -0.3415 | 0.0000 |
| 970 | SMG6 | 5 | 0.3078 | 0.6916 | -0.1651 | 0.0028 |
| 971 | SMG8 | 2 | -0.0663 | 0.5300 | -0.0663 | 0.5300 |
| 972 | SMIM12 | 3 | 0.2770 | 0.8844 | -0.2139 | 0.0075 |
| 973 | SMIM13 | 2 | -0.3180 | 0.0020 | -0.3180 | 0.0020 |
| 974 | SNAP47 | 3 | 0.0580 | 0.7226 | -0.1009 | 0.3384 |
| 975 | SNAPC4 | 3 | 0.3917 | 0.3788 | 0.0928 | 0.0001 |
| 976 | SNPH | 3 | 0.1175 | 0.7243 | -0.1130 | 0.2645 |
| 977 | SNX19 | 2 | 0.2835 | 0.0062 | 0.2835 | 0.0062 |
| 978 | SNX21 | 2 | 0.0861 | 0.4147 | 0.0861 | 0.4147 |
| 979 | SNX33 | 2 | -0.0918 | 0.3840 | -0.0918 | 0.3840 |
| 980 | SOCS5 | 2 | 0.0099 | 0.9251 | 0.0099 | 0.9251 |
| 981 | SOCS6 | 3 | 0.1461 | 0.1645 | -0.2281 | 0.0287 |
| 982 | SOGA1 | 4 | 0.2930 | 0.8707 | -0.3921 | 0.0001 |
| 983 | SON | 5 | 0.2252 | 0.7751 | -0.1245 | 0.0309 |
| 984 | SOS1 | 5 | 0.3160 | 0.9711 | -0.1903 | 0.0022 |
| 985 | SOX4 | 2 | 0.1018 | 0.3344 | 0.1018 | 0.3344 |
| 986 | SP1 | 3 | 0.2501 | 0.4152 | 0.0860 | 0.0162 |
| 987 | SP4 | 2 | -0.0527 | 0.6176 | -0.0527 | 0.6176 |
| 988 | SPATA13 | 2 | -0.0211 | 0.8420 | -0.0211 | 0.8420 |
| 989 | SPATA2 | 2 | -0.1070 | 0.3102 | -0.1070 | 0.3102 |
| 990 | SPECC1 | 2 | -0.2568 | 0.0135 | -0.2568 | 0.0135 |
| 991 | SPEN | 5 | 0.1954 | 0.9860 | -0.2964 | 0.0041 |
| 992 | SPG7 | 3 | 0.3000 | 0.7956 | -0.0378 | 0.0037 |
| 993 | SPOCK2 | 2 | 0.4602 | 0.0000 | 0.4602 | 0.0000 |
| 994 | SPOP | 3 | 0.1869 | 0.2955 | -0.2378 | 0.0225 |
| 995 | SPPL3 | 3 | 0.1584 | 0.3567 | -0.3450 | 0.0008 |
| 996 | SPR | 3 | 0.1139 | 0.7929 | -0.0854 | 0.2797 |
| 997 | SPSB1 | 5 | 0.6385 | 0.7856 | -0.5479 | 0.0000 |
| 998 | SPTBN1 | 4 | 0.2701 | 0.7945 | -0.4216 | 0.0000 |
| 999 | SPTLC2 | 2 | -0.1282 | 0.2232 | -0.1282 | 0.2232 |
| 1000 | SRBD1 | 2 | -0.0600 | 0.5700 | -0.0600 | 0.5700 |
| 1001 | SRCAP | 8 | 0.6741 | 0.8313 | -0.4133 | 0.0000 |
| 1002 | SRFBP1 | 2 | -0.0108 | 0.9188 | -0.0108 | 0.9188 |
| 1003 | SRRD | 2 | -0.1119 | 0.2882 | -0.1119 | 0.2882 |
| 1004 | SRRM2 | 9 | 0.5218 | 0.9997 | -0.2096 | 0.0000 |
| 1005 | SRXN1 | 4 | 0.2974 | 0.7800 | -0.2275 | 0.0040 |
| 1006 | SSH1 | 4 | 0.0303 | 0.7742 | -0.3285 | 0.0014 |
| 1007 | SSH2 | 4 | 0.3258 | 0.5966 | -0.1138 | 0.0015 |
| 1008 | STAM | 2 | 0.0710 | 0.5014 | 0.0710 | 0.5014 |
| 1009 | STARD7 | 3 | 0.3345 | 0.4538 | -0.3444 | 0.0008 |
| 1010 | STC2 | 2 | 0.2034 | 0.0518 | 0.2034 | 0.0518 |
| 1011 | STK10 | 4 | 0.5230 | 0.7017 | -0.0765 | 0.0000 |
| 1012 | STK16 | 2 | 0.2031 | 0.0522 | 0.2031 | 0.0522 |
| 1013 | STPG1 | 2 | -0.0858 | 0.4164 | -0.0858 | 0.4164 |
| 1014 | STRADA | 2 | 0.1852 | 0.0772 | 0.1852 | 0.0772 |
| 1015 | STS | 2 | 0.1211 | 0.2501 | 0.1211 | 0.2501 |
| 1016 | SUGP2 | 3 | 0.0534 | 0.6132 | -0.3271 | 0.0015 |
| 1017 | SURF6 | 3 | 0.1980 | 0.2608 | -0.1785 | 0.0585 |
| 1018 | SYNJ2 | 2 | 0.2902 | 0.0050 | 0.2902 | 0.0050 |
| 1019 | SYNRG | 5 | 0.6606 | 0.4653 | -0.4940 | 0.0000 |
| 1020 | TAB2 | 2 | -0.1334 | 0.2050 | -0.1334 | 0.2050 |
| 1021 | TACC1 | 2 | 0.4516 | 0.0000 | 0.4516 | 0.0000 |
| 1022 | TADA2B | 4 | 0.2295 | 0.9485 | -0.2962 | 0.0041 |
| 1023 | TAF15 | 3 | 0.6988 | 0.4111 | 0.0867 | 0.0000 |
| 1024 | TAF1C | 4 | 0.4481 | 0.8504 | -0.4064 | 0.0000 |
| 1025 | TAF4 | 2 | -0.2111 | 0.0434 | -0.2111 | 0.0434 |
| 1026 | TAF5L | 5 | 0.2481 | 0.8244 | -0.4549 | 0.0000 |
| 1027 | TANC2 | 5 | 0.4110 | 0.5677 | -0.3359 | 0.0000 |
| 1028 | TAPBP | 3 | -0.0505 | 0.6326 | -0.3649 | 0.0003 |
| 1029 | TARS | 2 | -0.1581 | 0.1322 | -0.1581 | 0.1322 |
| 1030 | TATDN2 | 2 | 0.0533 | 0.6141 | 0.0533 | 0.6141 |
| 1031 | TBC1D10B | 5 | 0.4037 | 0.8956 | -0.2091 | 0.0001 |
| 1032 | TBC1D13 | 3 | 0.0044 | 0.9665 | -0.1813 | 0.0837 |
| 1033 | TBC1D14 | 2 | 0.0694 | 0.5109 | 0.0694 | 0.5109 |
| 1034 | TBC1D16 | 8 | 0.4450 | 0.9809 | -0.3618 | 0.0000 |
| 1035 | TBC1D20 | 6 | 0.2425 | 0.7483 | -0.4600 | 0.0000 |
| 1036 | TBC1D8 | 2 | 0.2664 | 0.0103 | 0.2664 | 0.0103 |
| 1037 | TBC1D9B | 4 | 0.4192 | 0.9655 | -0.3483 | 0.0000 |
| 1038 | TBL1X | 2 | 0.2858 | 0.0057 | 0.2858 | 0.0057 |
| 1039 | TBL2 | 2 | 0.1523 | 0.1473 | 0.1523 | 0.1473 |
| 1040 | TCF19 | 3 | 0.1053 | 0.7853 | -0.1182 | 0.2619 |
| 1041 | TCF20 | 2 | -0.1251 | 0.2347 | -0.1251 | 0.2347 |
| 1042 | TCFL5 | 2 | -0.0287 | 0.7856 | -0.0287 | 0.7856 |
| 1043 | TECPR2 | 3 | 0.0876 | 0.4063 | -0.3821 | 0.0002 |
| 1044 | TENM3 | 3 | 0.2187 | 0.9179 | 0.0109 | 0.0362 |
| 1045 | TENT5B | 2 | -0.1019 | 0.3336 | -0.1019 | 0.3336 |
| 1046 | TESK1 | 2 | 0.0985 | 0.3505 | 0.0985 | 0.3505 |
| 1047 | TEX10 | 2 | -0.0444 | 0.6745 | -0.0444 | 0.6745 |
| 1048 | TGFBRAP1 | 3 | 0.1785 | 0.8429 | -0.2702 | 0.0092 |
| 1049 | TGOLN2 | 4 | 0.2812 | 0.8631 | -0.0289 | 0.0066 |
| 1050 | THADA | 2 | -0.0448 | 0.6717 | -0.0448 | 0.6717 |
| 1051 | THAP11 | 2 | 0.1892 | 0.0709 | 0.1892 | 0.0709 |
| 1052 | THAP3 | 2 | -0.3217 | 0.0018 | -0.3217 | 0.0018 |
| 1053 | THBS1 | 2 | 0.1931 | 0.0651 | 0.1931 | 0.0651 |
| 1054 | TICAM1 | 5 | 0.2902 | 0.5557 | -0.3962 | 0.0001 |
| 1055 | TIGD5 | 2 | 0.2355 | 0.0239 | 0.2355 | 0.0239 |
| 1056 | TIMM23 | 2 | -0.0643 | 0.5427 | -0.0643 | 0.5427 |
| 1057 | TIMM50 | 2 | -0.0797 | 0.4501 | -0.0797 | 0.4501 |
| 1058 | TIMM8A | 3 | 0.7659 | 0.1333 | -0.2740 | 0.0000 |
| 1059 | TIPARP | 2 | -0.1056 | 0.3166 | -0.1056 | 0.3166 |
| 1060 | TJAP1 | 2 | 0.2292 | 0.0280 | 0.2292 | 0.0280 |
| 1061 | TJP1 | 4 | 0.5448 | 0.7008 | -0.3253 | 0.0000 |
| 1062 | TKT | 2 | 0.0382 | 0.7178 | 0.0382 | 0.7178 |
| 1063 | TLNRD1 | 2 | 0.1362 | 0.1954 | 0.1362 | 0.1954 |
| 1064 | TM9SF4 | 4 | 0.3755 | 0.4700 | -0.1710 | 0.0002 |
| 1065 | TMED1 | 2 | -0.0112 | 0.9153 | -0.0112 | 0.9153 |
| 1066 | TMEM115 | 3 | 0.2630 | 0.4193 | -0.2028 | 0.0113 |
| 1067 | TMEM120B | 3 | 0.4373 | 0.9108 | 0.0119 | 0.0000 |
| 1068 | TMEM131L | 2 | 0.3845 | 0.0002 | 0.3845 | 0.0002 |
| 1069 | TMEM184C | 2 | -0.0908 | 0.3893 | -0.0908 | 0.3893 |
| 1070 | TMEM201 | 2 | -0.2919 | 0.0048 | -0.2919 | 0.0048 |
| 1071 | TMEM203 | 2 | 0.3556 | 0.0005 | 0.3556 | 0.0005 |
| 1072 | TMEM231 | 2 | -0.1228 | 0.2435 | -0.1228 | 0.2435 |
| 1073 | TMEM246 | 2 | -0.2626 | 0.0114 | -0.2626 | 0.0114 |
| 1074 | TMEM250 | 2 | 0.0500 | 0.6357 | 0.0500 | 0.6357 |
| 1075 | TMEM268 | 2 | 0.1945 | 0.0631 | 0.1945 | 0.0631 |
| 1076 | TMEM41A | 4 | 0.2466 | 0.6950 | -0.1582 | 0.0178 |
| 1077 | TMEM51 | 2 | 0.1405 | 0.1817 | 0.1405 | 0.1817 |
| 1078 | TMPO.AS1 | 2 | 0.0612 | 0.5624 | 0.0612 | 0.5624 |
| 1079 | TNF | 2 | -0.0090 | 0.9325 | -0.0090 | 0.9325 |
| 1080 | TNFAIP3 | 2 | 0.0633 | 0.5488 | 0.0633 | 0.5488 |
| 1081 | TNFAIP8L1 | 2 | 0.0257 | 0.8082 | 0.0257 | 0.8082 |
| 1082 | TNFRSF10B | 2 | -0.1365 | 0.1946 | -0.1365 | 0.1946 |
| 1083 | TNFRSF21 | 3 | 0.1126 | 0.3345 | -0.1436 | 0.1720 |
| 1084 | TNK2 | 2 | 0.5265 | 0.0000 | 0.5265 | 0.0000 |
| 1085 | TNKS1BP1 | 8 | 0.2492 | 0.9034 | -0.2585 | 0.0129 |
| 1086 | TNRC18 | 4 | 0.2001 | 0.6228 | -0.1595 | 0.0558 |
| 1087 | TNRC6A | 5 | 0.2632 | 0.7528 | -0.3255 | 0.0015 |
| 1088 | TNRC6B | 3 | 0.1059 | 0.5244 | -0.2305 | 0.0271 |
| 1089 | TNRC6C | 3 | 0.1842 | 0.6541 | -0.1790 | 0.0788 |
| 1090 | TNS3 | 3 | 0.2693 | 0.6378 | -0.1721 | 0.0094 |
| 1091 | TOB2 | 2 | 0.3033 | 0.0033 | 0.3033 | 0.0033 |
| 1092 | TOLLIP | 2 | 0.2504 | 0.0161 | 0.2504 | 0.0161 |
| 1093 | TOM1L2 | 3 | 0.0414 | 0.6949 | -0.2897 | 0.0051 |
| 1094 | TOP3A | 4 | 0.1551 | 0.8571 | -0.4272 | 0.0000 |
| 1095 | TOPORS | 3 | 0.0400 | 0.7083 | -0.1153 | 0.2738 |
| 1096 | TOR1AIP2 | 5 | 0.3296 | 0.7824 | -0.3329 | 0.0012 |
| 1097 | TOR1B | 2 | -0.1519 | 0.1483 | -0.1519 | 0.1483 |
| 1098 | TPBG | 2 | -0.2372 | 0.0228 | -0.2372 | 0.0228 |
| 1099 | TPD52 | 2 | 0.2164 | 0.0383 | 0.2164 | 0.0383 |
| 1100 | TRABD | 2 | -0.0389 | 0.7128 | -0.0389 | 0.7128 |
| 1101 | TRAF1 | 4 | 0.2636 | 0.7816 | -0.2480 | 0.0111 |
| 1102 | TRAF4 | 2 | 0.4578 | 0.0000 | 0.4578 | 0.0000 |
| 1103 | TRAF5 | 2 | -0.2091 | 0.0454 | -0.2091 | 0.0454 |
| 1104 | TRAK1 | 3 | 0.2450 | 0.9257 | -0.2131 | 0.0186 |
| 1105 | TRAM2 | 2 | -0.2887 | 0.0053 | -0.2887 | 0.0053 |
| 1106 | TRIB1 | 3 | 0.7301 | 0.0243 | 0.2348 | 0.0000 |
| 1107 | TRIM11 | 3 | 0.0810 | 0.9882 | -0.1161 | 0.2705 |
| 1108 | TRIM13 | 2 | 0.8100 | 0.0000 | 0.8100 | 0.0000 |
| 1109 | TRIM2 | 2 | 0.0803 | 0.4467 | 0.0803 | 0.4467 |
| 1110 | TRIM21 | 2 | 0.1795 | 0.0868 | 0.1795 | 0.0868 |
| 1111 | TRIM26 | 4 | 0.2608 | 0.2820 | -0.5776 | 0.0000 |
| 1112 | TRIM32 | 4 | 0.2631 | 0.7230 | -0.3189 | 0.0019 |
| 1113 | TRIM41 | 2 | 0.1722 | 0.1008 | 0.1722 | 0.1008 |
| 1114 | TRIM56 | 6 | 0.3640 | 0.9814 | -0.2306 | 0.0004 |
| 1115 | TRIM65 | 2 | 0.0623 | 0.5554 | 0.0623 | 0.5554 |
| 1116 | TRIM8 | 2 | 0.1525 | 0.1466 | 0.1525 | 0.1466 |
| 1117 | TRIO | 5 | 0.3858 | 0.5570 | -0.3371 | 0.0001 |
| 1118 | TRMT61A | 2 | 0.3981 | 0.0001 | 0.3981 | 0.0001 |
| 1119 | TRMT61B | 2 | 0.1331 | 0.2059 | 0.1331 | 0.2059 |
| 1120 | TRPS1 | 2 | 0.0246 | 0.8163 | 0.0246 | 0.8163 |
| 1121 | TRRAP | 2 | 0.2343 | 0.0246 | 0.2343 | 0.0246 |
| 1122 | TRUB2 | 2 | 0.1979 | 0.0587 | 0.1979 | 0.0587 |
| 1123 | TSC1 | 2 | -0.0284 | 0.7885 | -0.0284 | 0.7885 |
| 1124 | TSC22D2 | 2 | -0.1074 | 0.3080 | -0.1074 | 0.3080 |
| 1125 | TSHZ1 | 2 | -0.0553 | 0.6009 | -0.0553 | 0.6009 |
| 1126 | TSKU | 3 | 0.2547 | 0.9870 | -0.0017 | 0.0143 |
| 1127 | TSPAN14 | 3 | 0.3454 | 0.2141 | 0.1308 | 0.0007 |
| 1128 | TSPYL1 | 2 | 0.0467 | 0.6585 | 0.0467 | 0.6585 |
| 1129 | TSPYL4 | 3 | 0.1689 | 0.7028 | -0.0403 | 0.1076 |
| 1130 | TTC28 | 4 | 0.4465 | 0.5884 | 0.0572 | 0.0000 |
| 1131 | TTPAL | 2 | 0.2594 | 0.0125 | 0.2594 | 0.0125 |
| 1132 | TUBB4B | 3 | 0.1354 | 0.6434 | 0.0489 | 0.1980 |
| 1133 | TUBG1 | 2 | 0.1093 | 0.2997 | 0.1093 | 0.2997 |
| 1134 | TUBGCP6 | 3 | 0.5981 | 0.0000 | 0.4327 | 0.0000 |
| 1135 | TUG1 | 5 | 0.0898 | 0.9905 | -0.2903 | 0.0050 |
| 1136 | TUT4 | 3 | 0.3219 | 0.7129 | -0.0389 | 0.0018 |
| 1137 | TWNK | 4 | 0.4680 | 0.8731 | -0.2106 | 0.0000 |
| 1138 | TXLNA | 2 | -0.0660 | 0.5322 | -0.0660 | 0.5322 |
| 1139 | UBE2G2 | 2 | -0.1544 | 0.1416 | -0.1544 | 0.1416 |
| 1140 | UBE2O | 3 | 0.2535 | 0.9622 | 0.0050 | 0.0148 |
| 1141 | UBIAD1 | 2 | 0.1357 | 0.1970 | 0.1357 | 0.1970 |
| 1142 | UBL4A | 4 | 0.4557 | 0.2520 | -0.3436 | 0.0000 |
| 1143 | UBN1 | 4 | 0.4281 | 0.9874 | 0.0017 | 0.0000 |
| 1144 | UBTF | 3 | 0.1118 | 0.6741 | -0.1175 | 0.2645 |
| 1145 | UHRF1BP1 | 2 | -0.2195 | 0.0355 | -0.2195 | 0.0355 |
| 1146 | ULK1 | 2 | -0.1474 | 0.1609 | -0.1474 | 0.1609 |
| 1147 | UPF1 | 3 | 0.2330 | 0.0513 | -0.3426 | 0.0008 |
| 1148 | UPF2 | 2 | -0.0718 | 0.4962 | -0.0718 | 0.4962 |
| 1149 | URB1 | 6 | 0.2174 | 0.9867 | -0.4065 | 0.0001 |
| 1150 | URB2 | 3 | 0.0729 | 0.4897 | -0.4339 | 0.0000 |
| 1151 | URGCP | 6 | 0.3050 | 0.9792 | -0.2765 | 0.0031 |
| 1152 | USF1 | 2 | 0.2157 | 0.0389 | 0.2157 | 0.0389 |
| 1153 | USF3 | 2 | -0.1639 | 0.1186 | -0.1639 | 0.1186 |
| 1154 | USP24 | 2 | 0.1901 | 0.0696 | 0.1901 | 0.0696 |
| 1155 | USP31 | 4 | 0.3443 | 0.4789 | -0.3047 | 0.0008 |
| 1156 | USP36 | 2 | 0.2801 | 0.0068 | 0.2801 | 0.0068 |
| 1157 | USP54 | 2 | -0.3399 | 0.0009 | -0.3399 | 0.0009 |
| 1158 | UTP3 | 2 | -0.0879 | 0.4046 | -0.0879 | 0.4046 |
| 1159 | UVRAG | 2 | 0.2856 | 0.0058 | 0.2856 | 0.0058 |
| 1160 | VANGL1 | 2 | 0.4669 | 0.0000 | 0.4669 | 0.0000 |
| 1161 | VCAN | 2 | -0.1594 | 0.1290 | -0.1594 | 0.1290 |
| 1162 | VCL | 2 | -0.0867 | 0.4115 | -0.0867 | 0.4115 |
| 1163 | VCPIP1 | 5 | 0.3649 | 0.9885 | -0.0899 | 0.0003 |
| 1164 | VEGFA | 2 | 0.4026 | 0.0001 | 0.4026 | 0.0001 |
| 1165 | VIM | 2 | 0.5646 | 0.0000 | 0.5646 | 0.0000 |
| 1166 | VPS18 | 3 | 0.2295 | 0.3286 | -0.1395 | 0.0278 |
| 1167 | VPS39 | 2 | -0.3715 | 0.0003 | -0.3715 | 0.0003 |
| 1168 | VPS4A | 2 | 0.0720 | 0.4952 | 0.0720 | 0.4952 |
| 1169 | VPS53 | 2 | -0.3227 | 0.0017 | -0.3227 | 0.0017 |
| 1170 | VSIR | 3 | -0.0318 | 0.7633 | -0.4063 | 0.0001 |
| 1171 | WDR25 | 2 | -0.1691 | 0.1071 | -0.1691 | 0.1071 |
| 1172 | WDR33 | 7 | 0.3079 | 0.8493 | -0.4506 | 0.0000 |
| 1173 | WDR5 | 2 | 0.6495 | 0.0000 | 0.6495 | 0.0000 |
| 1174 | WDR6 | 2 | 0.2149 | 0.0396 | 0.2149 | 0.0396 |
| 1175 | WDR81 | 4 | 0.2796 | 0.9123 | -0.1040 | 0.0069 |
| 1176 | WHAMM | 2 | -0.1136 | 0.2807 | -0.1136 | 0.2807 |
| 1177 | WIPF2 | 2 | 0.0220 | 0.8351 | 0.0220 | 0.8351 |
| 1178 | WNK1 | 3 | 0.3273 | 0.0021 | -0.3569 | 0.0005 |
| 1179 | WNT7B | 5 | 0.3753 | 0.6788 | -0.0438 | 0.0002 |
| 1180 | XBP1 | 3 | 0.2319 | 0.1658 | -0.2007 | 0.0261 |
| 1181 | XIST | 6 | 0.2443 | 0.9296 | -0.2622 | 0.0116 |
| 1182 | XXYLT1 | 2 | 0.0845 | 0.4230 | 0.0845 | 0.4230 |
| 1183 | YARS | 2 | -0.3986 | 0.0001 | -0.3986 | 0.0001 |
| 1184 | YLPM1 | 3 | 0.1013 | 0.4689 | -0.2018 | 0.0537 |
| 1185 | YTHDF1 | 4 | 0.3266 | 0.9400 | -0.2376 | 0.0015 |
| 1186 | YTHDF3 | 4 | 0.1336 | 0.9582 | -0.3716 | 0.0003 |
| 1187 | YWHAH | 3 | 0.2863 | 0.2185 | -0.2352 | 0.0057 |
| 1188 | YY1AP1 | 2 | -0.1650 | 0.1161 | -0.1650 | 0.1161 |
| 1189 | ZBTB1 | 2 | 0.0424 | 0.6884 | 0.0424 | 0.6884 |
| 1190 | ZBTB18 | 4 | 0.1197 | 0.7751 | -0.1360 | 0.1962 |
| 1191 | ZBTB2 | 3 | 0.0626 | 0.9940 | -0.0008 | 0.5533 |
| 1192 | ZBTB21 | 4 | 0.1998 | 0.9365 | -0.0905 | 0.0562 |
| 1193 | ZBTB22 | 3 | 0.0624 | 0.5547 | -0.2885 | 0.0053 |
| 1194 | ZBTB25 | 2 | -0.0636 | 0.5471 | -0.0636 | 0.5471 |
| 1195 | ZBTB38 | 5 | 0.4112 | 0.8216 | -0.1102 | 0.0000 |
| 1196 | ZBTB40 | 2 | 0.0144 | 0.8915 | 0.0144 | 0.8915 |
| 1197 | ZBTB5 | 3 | 0.4709 | 0.6333 | -0.0543 | 0.0000 |
| 1198 | ZBTB7A | 2 | 0.2762 | 0.0077 | 0.2762 | 0.0077 |
| 1199 | ZBTB7B | 2 | 0.3121 | 0.0025 | 0.3121 | 0.0025 |
| 1200 | ZC3H13 | 3 | 0.0915 | 0.8896 | -0.1864 | 0.0752 |
| 1201 | ZC3H4 | 5 | 0.2450 | 0.4858 | -0.2010 | 0.0186 |
| 1202 | ZC3HAV1 | 2 | 0.0226 | 0.8307 | 0.0226 | 0.8307 |
| 1203 | ZCCHC14 | 2 | 0.0865 | 0.4124 | 0.0865 | 0.4124 |
| 1204 | ZCCHC8 | 9 | 0.5418 | 0.9977 | -0.4418 | 0.0000 |
| 1205 | ZDHHC3 | 3 | 0.4996 | 0.8172 | -0.0382 | 0.0000 |
| 1206 | ZDHHC5 | 2 | -0.3738 | 0.0002 | -0.3738 | 0.0002 |
| 1207 | ZER1 | 2 | 0.1484 | 0.1580 | 0.1484 | 0.1580 |
| 1208 | ZFHX3 | 3 | 0.3214 | 0.4600 | -0.1919 | 0.0018 |
| 1209 | ZFP36L1 | 4 | 0.4138 | 0.6088 | -0.1133 | 0.0000 |
| 1210 | ZFP36L2 | 3 | 0.1676 | 0.8143 | -0.1339 | 0.1102 |
| 1211 | ZFP64 | 6 | 0.4089 | 0.8604 | -0.2610 | 0.0001 |
| 1212 | ZHX3 | 3 | 0.0274 | 0.7952 | -0.0341 | 0.7468 |
| 1213 | ZKSCAN1 | 2 | -0.2294 | 0.0278 | -0.2294 | 0.0278 |
| 1214 | ZMIZ1 | 10 | 0.4371 | 0.9545 | -0.5164 | 0.0000 |
| 1215 | ZMIZ2 | 3 | 0.1988 | 0.2905 | 0.1114 | 0.0575 |
| 1216 | ZMYND19 | 2 | 0.1257 | 0.2327 | 0.1257 | 0.2327 |
| 1217 | ZMYND8 | 5 | 0.3072 | 0.9602 | -0.2969 | 0.0029 |
| 1218 | ZNF133 | 3 | 0.2390 | 0.8120 | -0.0733 | 0.0218 |
| 1219 | ZNF142 | 4 | 0.1618 | 0.9571 | -0.2246 | 0.0314 |
| 1220 | ZNF148 | 2 | -0.0033 | 0.9751 | -0.0033 | 0.9751 |
| 1221 | ZNF189 | 2 | 0.2902 | 0.0050 | 0.2902 | 0.0050 |
| 1222 | ZNF212 | 2 | -0.1133 | 0.2821 | -0.1133 | 0.2821 |
| 1223 | ZNF263 | 2 | 0.2129 | 0.0416 | 0.2129 | 0.0416 |
| 1224 | ZNF275 | 2 | 0.1499 | 0.1538 | 0.1499 | 0.1538 |
| 1225 | ZNF281 | 3 | 0.2044 | 0.7843 | -0.1670 | 0.0506 |
| 1226 | ZNF3 | 2 | 0.2242 | 0.0317 | 0.2242 | 0.0317 |
| 1227 | ZNF316 | 2 | -0.3507 | 0.0006 | -0.3507 | 0.0006 |
| 1228 | ZNF317 | 4 | 0.3139 | 0.5625 | -0.2304 | 0.0023 |
| 1229 | ZNF318 | 2 | 0.2390 | 0.0218 | 0.2390 | 0.0218 |
| 1230 | ZNF324 | 3 | 0.3412 | 0.4499 | -0.1035 | 0.0009 |
| 1231 | ZNF324B | 4 | 0.0653 | 0.5779 | -0.1615 | 0.1240 |
| 1232 | ZNF343 | 2 | -0.2410 | 0.0207 | -0.2410 | 0.0207 |
| 1233 | ZNF398 | 2 | 0.1738 | 0.0975 | 0.1738 | 0.0975 |
| 1234 | ZNF445 | 4 | 0.1970 | 0.8143 | -0.1368 | 0.0598 |
| 1235 | ZNF451 | 2 | 0.0410 | 0.6977 | 0.0410 | 0.6977 |
| 1236 | ZNF469 | 2 | -0.0696 | 0.5096 | -0.0696 | 0.5096 |
| 1237 | ZNF48 | 3 | 0.1464 | 0.4150 | -0.0860 | 0.1637 |
| 1238 | ZNF496 | 5 | 0.2721 | 0.8621 | -0.2579 | 0.0087 |
| 1239 | ZNF503 | 2 | 0.3317 | 0.0012 | 0.3317 | 0.0012 |
| 1240 | ZNF512B | 2 | -0.2540 | 0.0146 | -0.2540 | 0.0146 |
| 1241 | ZNF516 | 2 | -0.2565 | 0.0136 | -0.2565 | 0.0136 |
| 1242 | ZNF526 | 2 | -0.4180 | 0.0000 | -0.4180 | 0.0000 |
| 1243 | ZNF544 | 2 | -0.1711 | 0.1030 | -0.1711 | 0.1030 |
| 1244 | ZNF584 | 3 | 0.0719 | 0.6285 | -0.0709 | 0.4959 |
| 1245 | ZNF592 | 2 | 0.0899 | 0.3941 | 0.0899 | 0.3941 |
| 1246 | ZNF598 | 2 | -0.1609 | 0.1256 | -0.1609 | 0.1256 |
| 1247 | ZNF608 | 3 | 0.2329 | 0.0548 | -0.2500 | 0.0163 |
| 1248 | ZNF609 | 4 | 0.3635 | 0.7239 | -0.1061 | 0.0004 |
| 1249 | ZNF618 | 3 | 0.2056 | 0.0493 | -0.4558 | 0.0000 |
| 1250 | ZNF623 | 3 | 0.3406 | 0.2477 | -0.3049 | 0.0009 |
| 1251 | ZNF646 | 3 | 0.1462 | 0.5261 | 0.0669 | 0.1644 |
| 1252 | ZNF649 | 2 | 0.0703 | 0.5055 | 0.0703 | 0.5055 |
| 1253 | ZNF668 | 2 | 0.0682 | 0.5186 | 0.0682 | 0.5186 |
| 1254 | ZNF687 | 4 | 0.7194 | 0.6888 | -0.2274 | 0.0000 |
| 1255 | ZNF691 | 2 | 0.0481 | 0.6489 | 0.0481 | 0.6489 |
| 1256 | ZNF703 | 2 | -0.1440 | 0.1708 | -0.1440 | 0.1708 |
| 1257 | ZNF746 | 4 | 0.3428 | 0.7453 | -0.2364 | 0.0008 |
| 1258 | ZNF777 | 3 | 0.3108 | 0.9445 | -0.2677 | 0.0026 |
| 1259 | ZNF786 | 2 | -0.2249 | 0.0312 | -0.2249 | 0.0312 |
| 1260 | ZNF821 | 2 | -0.0090 | 0.9318 | -0.0090 | 0.9318 |
| 1261 | ZNF827 | 3 | 0.1203 | 0.2617 | -0.1501 | 0.1533 |
| 1262 | ZNF865 | 2 | 0.2324 | 0.0258 | 0.2324 | 0.0258 |
| 1263 | ZNFX1 | 3 | -0.0358 | 0.7345 | -0.3137 | 0.0023 |
| 1264 | ZNRF3 | 2 | 0.2463 | 0.0180 | 0.2463 | 0.0180 |
| 1265 | ZSCAN2 | 2 | 0.1198 | 0.2552 | 0.1198 | 0.2552 |
| 1266 | ZSCAN25 | 2 | -0.2076 | 0.0470 | -0.2076 | 0.0470 |
| 1267 | ZSCAN29 | 2 | 0.2101 | 0.0444 | 0.2101 | 0.0444 |
| 1268 | ZWINT | 2 | 0.3581 | 0.0005 | 0.3581 | 0.0005 |
| 1269 | ZZZ3 | 2 | -0.1571 | 0.1348 | -0.1571 | 0.1348 |

**Corr: Max**: Maximum correlation, the strongest linear relationship between each site**; Corr: p-value: Max:** Correlation p-value at Maximum, indicating significance for the maximum correlation; **Corr: Min**: Minimum correlation, the weakest linear relationship between each site; **Corr: p-value: Min**: Correlation p-value at Minimum, denoting significance for the minimum correlation; **LM: adj.R-square:** Linear Model Adjusted R-squared, measuring explained variance in the dependent number of sites; **LM:** **p-value:** Linear Model p-value, assessing the statistical significance of regression coefficients.

**Table S4**: Enriched GO Processes and KEGG Pathways Associated with Clinical and Histopathological Significant Modules in Glioblastoma

| **Term** | **Library** | **p-value** | **q-value** | **z-score** | **combined score** | **Chi-square comparison p-value** |
| --- | --- | --- | --- | --- | --- | --- |
| **MEmagenta correlated with Diagnosis (PD vs psPD)** |  |  |  |  |  |  |
| regulation of transcription by RNA polymerase II (GO:0006357) | BP 2021 | 0.0003189 | 0.09505 | 2.153 | 17.34 | 0.000620247 |
| vesicle organization (GO:0016050) | BP 2021 | 0.000369 | 0.09505 | 13.1 | 103.6 | 3.60E-18 |
| positive regulation of transcription, DNA-templated (GO:0045893) | BP 2021 | 0.0004604 | 0.09505 | 2.511 | 19.29 | 2.55E-07 |
| positive regulation of transcription by RNA polymerase II (GO:0045944) | BP 2021 | 0.0004053 | 0.09505 | 2.762 | 21.57 | 7.16E-08 |
| negative regulation of transcription, DNA-templated (GO:0045892) | BP 2021 | 0.000229 | 0.09505 | 2.817 | 23.61 | 2.58E-10 |
| Wnt signaling pathway | KEGG 2021 | 0.007915 | 0.68 | 4.3 | 20.69 | 4.74E-249 |
| Signaling pathways regulating pluripotency of stem cells | KEGG 2021 | 0.0218 | 0.72 | 3.9 | 15.07 | 6.50E-250 |
| Mucin type O-glycan biosynthesis | KEGG 2021 | 0.02908 | 0.72 | 8 | 28.25 | 1.21E-251 |
| AMPK signaling pathway | KEGG 2021 | 0.0122 | 0.68 | 4.7 | 20.82 | 4.74E-249 |
| Aminoacyl-tRNA biosynthesis | KEGG 2021 | 0.01293 | 0.68 | 6.5 | 28.26 | 8.89E-251 |
|  |  |  |  |  |  |  |
| **Genes identified by the Boruta analysis with MEmagenta correlated with Diagnosis (PD vs psPD)** |  |  |  |  |  |  |
| regulation of transcription by RNA polymerase II (GO:0006357) | BP 2021 | 6.89E-11 | 2.90E-07 | 1.641 | 38.4 | 6.89E-11 |
| negative regulation of transcription, DNA-templated (GO:0045892) | BP 2021 | 7.60E-10 | 1.5968E-06 | 1.918 | 40.27 | 7.60E-10 |
| regulation of transcription, DNA-templated (GO:0006355) | BP 2021 | 3.94E-09 | 5.5211E-06 | 1.563 | 30.25 | 3.94E-09 |
| positive regulation of transcription, DNA-templated (GO:0045893) | BP 2021 | 8.69E-09 | 9.1294E-06 | 1.753 | 32.53 | 8.69E-09 |
| negative regulation of transcription by RNA polymerase II (GO:0000122) | BP 2021 | 5.69E-08 | 0.0000478 | 1.942 | 32.4 | 5.69E-08 |
| Glycosaminoglycan biosynthesis | KEGG 2021 | 6.1512E-06 | 0.001852 | 4.827 | 57.91 | 6.1512E-06 |
| Chronic myeloid leukemia | KEGG 2021 | 0.000045268 | 0.004735 | 3.524 | 35.25 | 0.000045268 |
| Cellular senescence | KEGG 2021 | 0.000047194 | 0.004735 | 2.567 | 25.57 | 0.000047194 |
| Growth hormone synthesis, secretion and action | KEGG 2021 | 0.000084543 | 0.006362 | 2.777 | 26.05 | 0.000084543 |
| Longevity regulating pathway | KEGG 2021 | 0.0002266 | 0.01364 | 2.8 | 23.5 | 0.0002266 |
|  |  |  |  |  |  |  |
| **MEpurple correlated with Diagnosis (PD vs psPD)** |  |  |  |  |  |  |
| regulation of mRNA catabolic process (GO:0061013) | BP 2021 | 0.0008787 | 0.06 | 17.9 | 125.8 | 1.61E-05 |
| positive regulation of nuclear-transcribed mRNA poly(A) tail shortening (GO:0060213) | BP 2021 | 0.0001794 | 0.03 | 125.1 | 1079 | 1.35E-22 |
| regulation of nuclear-transcribed mRNA poly(A) tail shortening (GO:0060211) | BP 2021 | 0.0002411 | 0.03 | 105.9 | 881.9 | 1.35E-22 |
| post-transcriptional gene silencing by RNA (GO:0035194) | BP 2021 | 0.0004341 | 0.04 | 76.4 | 591.8 | 1.35E-22 |
| positive regulation of mRNA catabolic process (GO:0061014) | BP 2021 | 0.000042772 | 0.02 | 52.1 | 523.9 | 8.81E-31 |
| Platelet activation | KEGG 2021 | 0.01577 | 0.33 | 11.2 | 46.56 | 1.17E-17 |
| Vitamin digestion and absorption | KEGG 2021 | 0.03656 | 0.33 | 28.9 | 95.64 | 1.67E-18 |
| Glycosylphosphatidylinositol (GPI)-anchor biosynthesis | KEGG 2021 | 0.03955 | 0.33 | 26.6 | 85.9 | 2.26E-19 |
| Fatty acid elongation | KEGG 2021 | 0.04104 | 0.33 | 25.6 | 81.64 | 1.67E-18 |
| Butanoate metabolism | KEGG 2021 | 0.04253 | 0.33 | 24.6 | 77.74 | 1.67E-18 |
|  |  |  |  |  |  |  |
| **MElightcyan correlated with Diagnosis (PD vs psPD)** |  |  |  |  |  |  |
| regulation of type I interferon production (GO:0032479) | BP 2021 | 0.0003499 | 0.03405 | 24.77 | 197.1 | 0.221905087 |
| positive regulation of interferon-beta production (GO:0032728) | BP 2021 | 0.001417 | 0.06068 | 40.44 | 265.2 | 0.755420189 |
| mammary gland development (GO:0030879) | BP 2021 | 0.0006282 | 0.04586 | 62.53 | 461 | 0.755420189 |
| lactation (GO:0007595) | BP 2021 | 0.0001038 | 0.03032 | 172.1 | 1578 | 0.755420189 |
| body fluid secretion (GO:0007589) | BP 2021 | 0.0002753 | 0.03405 | 98.3 | 805.8 | 0.755420189 |
| Phosphonate and phosphinate metabolism | KEGG 2021 | 0.009265 | 0.15 | 133.1 | 623.1 | 4.65E-58 |
| TNF signaling pathway | KEGG 2021 | 0.013 | 0.15 | 12.5 | 54.07 | 3.38E-57 |
| Notch signaling pathway | KEGG 2021 | 0.003765 | 0.15 | 24.1 | 134.5 | 3.38E-57 |
| Growth hormone synthesis, secretion and action | KEGG 2021 | 0.01459 | 0.15 | 11.7 | 49.47 | 3.38E-57 |
| Glycerophospholipid metabolism | KEGG 2021 | 0.01007 | 0.15 | 14.3 | 65.64 | 3.38E-57 |
|  |  |  |  |  |  |  |
| **MEmidnightblue correlated with Diagnosis (PD vs psPD)** |  |  |  |  |  |  |
| organelle organization (GO:0006996) | BP 2021 | 0.003851 | 0.09911 | 6.963 | 38.71 | 1.60E-47 |
| cytoskeleton organization (GO:0007010) | BP 2021 | 0.0008376 | 0.08627 | 18.18 | 128.8 | 1.98E-17 |
| regulation of RIG-I signaling pathway (GO:0039535) | BP 2021 | 0.0003911 | 0.08057 | 80.94 | 635.1 | 2.09E-07 |
| cytoskeleton-dependent cytokinesis (GO:0061640) | BP 2021 | 0.005555 | 0.09911 | 19.6 | 101.8 | 2.09E-07 |
| positive regulation of basement membrane assembly involved in embryonic body morphogenesis (GO:1904261) | BP 2021 | 0.007727 | 0.09911 | 166.4 | 809.1 | 0.102767181 |
| AMPK signaling pathway | KEGG 2021 | 0.01482 | 0.22 | 11.6 | 48.87 | 2.58E-18 |
| Maturity onset diabetes of the young | KEGG 2021 | 0.03955 | 0.22 | 26.6 | 85.9 | 3.65E-19 |
| Pentose phosphate pathway | KEGG 2021 | 0.0455 | 0.22 | 22.9 | 70.82 | 3.65E-19 |
| Galactose metabolism | KEGG 2021 | 0.04698 | 0.22 | 22.2 | 67.75 | 3.65E-19 |
| Protein processing in endoplasmic reticulum | KEGG 2021 | 0.02873 | 0.22 | 8.1 | 28.68 | 1.11E-16 |
|  |  |  |  |  |  |  |
| **MEgreen correlated with Diagnosis (PD vs psPD)** |  |  |  |  |  |  |
| mRNA methylation (GO:0080009) | BP 2021 | 0.0002411 | 0.02203 | 105.9 | 881.9 | 5.17E-11 |
| positive regulation of cellular biosynthetic process (GO:0031328) | BP 2021 | 0.0001651 | 0.0205 | 16.66 | 145.1 | 1.50E-48 |
| RNA methylation (GO:0001510) | BP 2021 | 0.0001087 | 0.0205 | 37.43 | 341.6 | 7.76E-27 |
| rRNA base methylation (GO:0070475) | BP 2021 | 6.47E-05 | 0.0205 | 229.5 | 2213 | 5.17E-11 |
| central nervous system neuron axonogenesis (GO:0021955) | BP 2021 | 0.0001794 | 0.0205 | 125.1 | 1079 | 5.17E-11 |
| Protein processing in endoplasmic reticulum | KEGG 2021 | 0.002315 | 0.07 | 12.6 | 76.63 | 2.23E-09 |
| Vitamin B6 metabolism | KEGG 2021 | 0.009265 | 0.14 | 133.1 | 623.1 | 5.24E-11 |
| Fructose and mannose metabolism | KEGG 2021 | 0.04994 | 0.37 | 20.8 | 62.24 | 5.24E-11 |
| Ferroptosis | KEGG 2021 | 0.06168 | 0.37 | 16.6 | 46.27 | N/A |
| Amino sugar and nucleotide sugar metabolism | KEGG 2021 | 0.07183 | 0.37 | 14.1 | 37.21 | N/A |
|  |  |  |  |  |  |  |
| **MEturquoise correlated with Diagnosis (PD vs psPD)** |  |  |  |  |  |  |
| positive regulation of GTPase activity (GO:0043547) | BP 2021 | 0.0003197 | 0.09626 | 13.94 | 112.2 | 0.236043642 |
| positive regulation of intracellular protein transport (GO:0090316) | BP 2021 | 0.001533 | 0.1205 | 14.65 | 94.93 | 0.642528733 |
| activation of GTPase activity (GO:0090630) | BP 2021 | 0.0005679 | 0.09626 | 20.87 | 156 | 0.01286882 |
| regulation of cilium assembly (GO:1902017) | BP 2021 | 0.004415 | 0.1205 | 22.14 | 120.1 | 1 |
| Golgi to plasma membrane protein transport (GO:0043001) | BP 2021 | 0.0009844 | 0.1112 | 49.12 | 340.1 | 1 |
| Hedgehog signaling pathway | KEGG 2021 | 0.08331 | 0.33 | 12.1 | 29.99 | N/A |
| Legionellosis | KEGG 2021 | 0.08474 | 0.33 | 11.9 | 29.26 | N/A |
| Notch signaling pathway | KEGG 2021 | 0.08758 | 0.33 | 11.4 | 27.87 | N/A |
| Lysine degradation | KEGG 2021 | 0.09324 | 0.33 | 10.7 | 25.39 | N/A |
| Alzheimer disease | KEGG 2021 | 0.1113 | 0.33 | 3.7 | 8.086 | N/A |
|  |  |  |  |  |  |  |
| **MEtan correlated with Diagnosis (PD vs psPD)** |  |  |  |  |  |  |
| metanephric epithelium development (GO:0072207) | BP 2021 | 0.0004999 | 0.002949 | 4999 | 37990 | 3.48E-17 |
| kidney morphogenesis (GO:0060993) | BP 2021 | 0.0004999 | 0.002949 | 4999 | 37990 | 3.48E-17 |
| lung morphogenesis (GO:0060425) | BP 2021 | 0.0004999 | 0.002949 | 4999 | 37990 | 3.48E-17 |
| forebrain regionalization (GO:0021871) | BP 2021 | 0.0004999 | 0.002949 | 4999 | 37990 | 3.48E-17 |
| isopentenyl diphosphate biosynthetic process (GO:0009240) | BP 2021 | 0.0004999 | 0.002949 | 4999 | 37990 | 2.44E-16 |
| Terpenoid backbone biosynthesis | KEGG 2021 | 0.002199 | 0.02 | 951.3 | 5822 | 4.04E-16 |
| Basal cell carcinoma | KEGG 2021 | 0.00629 | 0.02 | 321.5 | 1630 | 5.77E-17 |
| Melanogenesis | KEGG 2021 | 0.01007 | 0.02 | 199 | 914.9 | 5.77E-17 |
| Signaling pathways regulating pluripotency of stem cells | KEGG 2021 | 0.01425 | 0.02 | 139.8 | 594.4 | 5.77E-17 |
| Breast cancer | KEGG 2021 | 0.01465 | 0.02 | 136 | 574.3 | 5.77E-17 |
|  |  |  |  |  |  |  |
| **MEsalmon correlated with Gender** |  |  |  |  |  |  |
| regulation of lymphocyte migration (GO:2000401) | BP 2021 | 0.003296 | 0.02154 | 399.7 | 2284 | 3.65E-19 |
| T-helper cell lineage commitment (GO:0002295) | BP 2021 | 0.002398 | 0.02154 | 571.1 | 3445 | 3.65E-19 |
| negative regulation of production of miRNAs involved in gene silencing by miRNA (GO:1903799) | BP 2021 | 0.003595 | 0.02154 | 363.3 | 2045 | 3.65E-19 |
| negative regulation of purine nucleotide metabolic process (GO:1900543) | BP 2021 | 0.002997 | 0.02154 | 444.1 | 2580 | 3.65E-19 |
| negative regulation of glycolytic process (GO:0045820) | BP 2021 | 0.002997 | 0.02154 | 444.1 | 2580 | 3.65E-19 |
| Mitophagy | KEGG 2021 | 0.0001906 | 0.01 | 30.6 | 262.3 | 2.02E-14 |
| Thyroid hormone signaling pathway | KEGG 2021 | 0.01696 | 0.29 | 10.8 | 43.87 | 3.06E-15 |
| Estrogen signaling pathway | KEGG 2021 | 0.02141 | 0.29 | 9.5 | 36.43 | 3.06E-15 |
| Spinocerebellar ataxia | KEGG 2021 | 0.02319 | 0.29 | 9.1 | 34.15 | 3.06E-15 |
| Wnt signaling pathway | KEGG 2021 | 0.03056 | 0.29 | 7.8 | 27.17 | 3.06E-15 |
|  |  |  |  |  |  |  |
| **MElightgreen correlated with Gender** |  |  |  |  |  |  |
| fructose 2,6-bisphosphate metabolic process (GO:0006003) | BP 2021 | 0.008224 | 0.1149 | 156 | 748.7 | 7.46E-63 |
| protein localization to lysosome (GO:0061462) | BP 2021 | 0.0009715 | 0.1149 | 49.48 | 343.2 | 5.43E-62 |
| nucleocytoplasmic transport (GO:0006913) | BP 2021 | 0.00198 | 0.1149 | 33.84 | 210.6 | 5.43E-62 |
| positive regulation of RNA splicing (GO:0033120) | BP 2021 | 0.001191 | 0.1149 | 44.36 | 298.6 | 5.43E-62 |
| regulation of autophagy (GO:0010506) | BP 2021 | 0.006427 | 0.1149 | 8.657 | 43.7 | 2.76E-60 |
| Lysosome | KEGG 2021 | 0.01885 | 0.33 | 10.2 | 40.34 | 5.43E-62 |
| Fatty acid elongation | KEGG 2021 | 0.04363 | 0.33 | 24 | 75.06 | 7.46E-63 |
| Biosynthesis of unsaturated fatty acids | KEGG 2021 | 0.04363 | 0.33 | 24 | 75.06 | 7.46E-63 |
| Human immunodeficiency virus 1 infection | KEGG 2021 | 0.04763 | 0.33 | 6.1 | 18.48 | 5.43E-62 |
| Fructose and mannose metabolism | KEGG 2021 | 0.05308 | 0.33 | 19.5 | 57.16 | 5.43E-62 |
|  |  |  |  |  |  |  |
| **MEmagenta correlated with Gender** |  |  |  |  |  |  |
| regulation of transforming growth factor beta receptor signaling pathway (GO:0017015) | BP 2021 | 0.0006913 | 0.1699 | 7.706 | 56.07 | 0.460487091 |
| negative regulation of transcription, DNA-templated (GO:0045892) | BP 2021 | 0.0001126 | 0.13 | 3.003 | 27.3 | 3.26E-14 |
| rRNA metabolic process (GO:0016072) | BP 2021 | 0.0009888 | 0.1699 | 5.656 | 39.13 | 3.26E-07 |
| regulation of cellular response to transforming growth factor beta stimulus (GO:1903844) | BP 2021 | 0.00061 | 0.1699 | 20.69 | 153.1 | 0.584717746 |
| 5S class rRNA transcription by RNA polymerase III (GO:0042791) | BP 2021 | 0.0007164 | 0.1699 | 71.94 | 520.9 | 0.759541299 |
| Aminoacyl-tRNA biosynthesis | KEGG 2021 | 0.01114 | 0.69 | 6.9 | 30.95 | 8.89E-251 |
| Mucin type O-glycan biosynthesis | KEGG 2021 | 0.02624 | 0.69 | 8.5 | 30.77 | 1.21E-251 |
| Phosphatidylinositol signaling system | KEGG 2021 | 0.03072 | 0.69 | 4.6 | 16.04 | 8.89E-251 |
| Signaling pathways regulating pluripotency of stem cells | KEGG 2021 | 0.01819 | 0.69 | 4.2 | 16.72 | 6.50E-250 |
| Wnt signaling pathway | KEGG 2021 | 0.02942 | 0.69 | 3.6 | 12.61 | 6.50E-250 |
|  |  |  |  |  |  |  |
| **MEblack correlated with Gender** |  |  |  |  |  |  |
| transcription, DNA-templated (GO:0006351) | BP 2021 | 0.000029037 | 0.01 | 16.3 | 170.6 | 0.058087317 |
| DNA conformation change (GO:0071103) | BP 2021 | 0.0003973 | 0.09 | 80.5 | 630 | 0.005394729 |
| transcription by RNA polymerase II (GO:0006366) | BP 2021 | 0.001825 | 0.12 | 8.6 | 54.09 | 0.028499753 |
| cellular response to DNA damage stimulus (GO:0006974) | BP 2021 | 0.002527 | 0.12 | 7.8 | 46.78 | 0.028499753 |
| protein phosphorylation (GO:0006468) | BP 2021 | 0.001229 | 0.12 | 7.1 | 47.47 | 0.109543893 |
| Spinocerebellar ataxia | KEGG 2021 | 0.02319 | 0.27 | 9.1 | 34.15 | 1.94E-67 |
| Vitamin digestion and absorption | KEGG 2021 | 0.03888 | 0.27 | 27.1 | 87.99 | 2.66E-68 |
| Biosynthesis of unsaturated fatty acids | KEGG 2021 | 0.04363 | 0.27 | 24 | 75.06 | 2.66E-68 |
|  |  |  |  |  |  |  |
| **MEsalmon correlated with Side Location** |  |  |  |  |  |  |
| regulation of lipid metabolic process (GO:0019216) | BP 2021 | 0.0005756 | 0.0254 | 73.71 | 549.9 | 0.167571877 |
| regulation of primary metabolic process (GO:0080090) | BP 2021 | 0.001144 | 0.0254 | 51.73 | 350.4 | 0.167571877 |
| heart development (GO:0007507) | BP 2021 | 0.001812 | 0.0254 | 40.8 | 257.6 | 0.167571877 |
| circulatory system development (GO:0072359) | BP 2021 | 0.001075 | 0.0254 | 53.41 | 365 | 0.167571877 |
| left/right axis specification (GO:0070986) | BP 2021 | 0.001999 | 0.0254 | 713.9 | 4437 | 1 |
| Mitophagy | KEGG 2021 | 0.0003147 | 0.01 | 100.6 | 811.5 | 3.06E-15 |
| Transcriptional misregulation in cancer | KEGG 2021 | 0.002471 | 0.02 | 34.7 | 208.5 | 3.06E-15 |
| Mannose type O-glycan biosynthesis | KEGG 2021 | 0.009165 | 0.05 | 129.7 | 608.5 | 4.38E-16 |
| Cortisol synthesis and secretion | KEGG 2021 | 0.02571 | 0.08 | 44.5 | 162.8 | 4.38E-16 |
| TGF-beta signaling pathway | KEGG 2021 | 0.03699 | 0.08 | 30.6 | 100.8 | 4.38E-16 |
|  |  |  |  |  |  |  |
| **MEpurple correlated with Side Location** |  |  |  |  |  |  |
| cellular response to bacterial lipopeptide (GO:0071221) | BP 2021 | 0.002398 | 0.05 | 571.1 | 3445 | 1.67E-18 |
| response to lipoteichoic acid (GO:0070391) | BP 2021 | 0.002398 | 0.05 | 571.1 | 3445 | 1.67E-18 |
| positive regulation of toll-like receptor 3 signaling pathway (GO:0034141) | BP 2021 | 0.002398 | 0.05 | 571.1 | 3445 | 1.67E-18 |
| positive regulation of toll-like receptor 2 signaling pathway (GO:0034137) | BP 2021 | 0.002398 | 0.05 | 571.1 | 3445 | 1.67E-18 |
| cellular response to lipoteichoic acid (GO:0071223) | BP 2021 | 0.002398 | 0.05 | 571.1 | 3445 | 1.67E-18 |
| Glycosylphosphatidylinositol (GPI)-anchor biosynthesis | KEGG 2021 | 0.01035 | 0.05 | 114.1 | 521.5 | 2.26E-19 |
| Fatty acid elongation | KEGG 2021 | 0.01075 | 0.05 | 109.7 | 497.3 | 1.67E-18 |
| Butanoate metabolism | KEGG 2021 | 0.01115 | 0.05 | 105.6 | 475 | 1.67E-18 |
| Tryptophan metabolism | KEGG 2021 | 0.01668 | 0.05 | 69.5 | 284.6 | 1.67E-18 |
| Fatty acid degradation | KEGG 2021 | 0.01707 | 0.05 | 67.9 | 276.2 | 1.67E-18 |
|  |  |  |  |  |  |  |
| **MEyellow correlated with Time Survival** |  |  |  |  |  |  |
| entrainment of circadian clock by photoperiod (GO:0043153) | BP 2021 | 0.0003285 | 0.03 | 88.7 | 711.4 | 9.81E-10 |
| photoperiodism (GO:0009648) | BP 2021 | 0.0003535 | 0.03 | 85.3 | 677.7 | 9.81E-10 |
| protein K63-linked deubiquitination (GO:0070536) | BP 2021 | 0.0004628 | 0.03 | 73.9 | 567.3 | 9.81E-10 |
| nucleic acid phosphodiester bond hydrolysis (GO:0090305) | BP 2021 | 0.002523 | 0.09 | 30.3 | 181.3 | 9.81E-10 |
| heparin metabolic process (GO:0030202) | BP 2021 | 0.00499 | 0.09 | 262.8 | 1393 | 0.0432928 |
| Mismatch repair | KEGG 2021 | 0.02276 | 0.1 | 47.8 | 180.6 | 2.38E-21 |
| Circadian rhythm | KEGG 2021 | 0.03056 | 0.1 | 35 | 122.1 | 2.38E-21 |
| DNA replication | KEGG 2021 | 0.03541 | 0.1 | 30 | 100.2 | 2.38E-21 |
| Nucleotide excision repair | KEGG 2021 | 0.04599 | 0.1 | 22.8 | 70.23 | 2.38E-21 |
|  |  |  |  |  |  |  |
| **MEpurple correlated with Midline Shift** |  |  |  |  |  |  |
| cellular response to bacterial lipopeptide (GO:0071221) | BP 2021 | 0.002098 | 0.04 | 666.3 | 4109 | 1.67E-18 |
| response to lipoteichoic acid (GO:0070391) | BP 2021 | 0.002098 | 0.04 | 666.3 | 4109 | 1.67E-18 |
| positive regulation of toll-like receptor 3 signaling pathway (GO:0034141) | BP 2021 | 0.002098 | 0.04 | 666.3 | 4109 | 1.67E-18 |
| positive regulation of toll-like receptor 2 signaling pathway (GO:0034137) | BP 2021 | 0.002098 | 0.04 | 666.3 | 4109 | 1.67E-18 |
| cellular response to lipoteichoic acid (GO:0071223) | BP 2021 | 0.002098 | 0.04 | 666.3 | 4109 | 1.67E-18 |
| Glycosylphosphatidylinositol (GPI)-anchor biosynthesis | KEGG 2021 | 0.009066 | 0.04 | 133.1 | 626.1 | 2.26E-19 |
| Fatty acid elongation | KEGG 2021 | 0.009413 | 0.04 | 128 | 597.2 | 2.26E-19 |
| Butanoate metabolism | KEGG 2021 | 0.00976 | 0.04 | 123.2 | 570.6 | 2.26E-19 |
| Tryptophan metabolism | KEGG 2021 | 0.01461 | 0.04 | 81.1 | 342.8 | 2.26E-19 |
| Fatty acid degradation | KEGG 2021 | 0.01496 | 0.04 | 79.2 | 332.7 | 2.26E-19 |
|  |  |  |  |  |  |  |
| **MEpinkcorrelated with EGFR Status** |  |  |  |  |  |  |
| positive regulation of epithelial cell migration (GO:0010634) | BP 2021 | 0.0009597 | 0.04 | 54.1 | 375.7 | 0.248485344 |
| negative regulation of cell motility (GO:2000146) | BP 2021 | 0.001407 | 0.04 | 44.4 | 291.4 | 1 |
| negative regulation of cell migration (GO:0030336) | BP 2021 | 0.002231 | 0.04 | 34.9 | 213.3 | 1 |
| p38MAPK cascade (GO:0038066) | BP 2021 | 0.002498 | 0.04 | 555.2 | 3327 | 1 |
| response to salt stress (GO:0009651) | BP 2021 | 0.002498 | 0.04 | 555.2 | 3327 | 1 |
| Salmonella infection | KEGG 2021 | 0.006504 | 0.05 | 20 | 100.6 | 2.48E-30 |
| Phenylalanine metabolism | KEGG 2021 | 0.008469 | 0.05 | 138.7 | 661.8 | 2.48E-30 |
| Tyrosine metabolism | KEGG 2021 | 0.01786 | 0.07 | 63.4 | 255 | 2.48E-30 |
| Fc gamma R-mediated phagocytosis | KEGG 2021 | 0.04747 | 0.11 | 23 | 70.18 | 2.48E-30 |
|  |  |  |  |  |  |  |
| **MEpurple correlated with Ki-67 Status** |  |  |  |  |  |  |
| positive regulation of mRNA catabolic process (GO:0061014) | BP 2021 | 0.000042772 | 0.02 | 52.1 | 523.9 | 1.16E-19 |
| positive regulation of nuclear-transcribed mRNA poly(A) tail shortening (GO:0060213) | BP 2021 | 0.0001794 | 0.03 | 125.1 | 1079 | 2.36E-14 |
| regulation of nuclear-transcribed mRNA poly(A) tail shortening (GO:0060211) | BP 2021 | 0.0002411 | 0.03 | 105.9 | 881.9 | 2.36E-14 |
| post-transcriptional gene silencing by RNA (GO:0035194) | BP 2021 | 0.0004341 | 0.04 | 76.4 | 591.8 | 2.36E-14 |
| regulation of actin polymerization or depolymerization (GO:0008064) | BP 2021 | 0.0009197 | 0.06 | 50.9 | 356.1 | 2.36E-14 |
| regulation of mRNA catabolic process (GO:0061013) | BP 2021 | 0.0008787 | 0.06 | 17.9 | 125.8 | 0.001429978 |
| Platelet activation | KEGG 2021 | 0.01577 | 0.33 | 11.2 | 46.56 | 1.17E-17 |
| Glycosylphosphatidylinositol (GPI)-anchor biosynthesis | KEGG 2021 | 0.03955 | 0.33 | 26.6 | 85.9 | 2.26E-19 |
| Vitamin digestion and absorption | KEGG 2021 | 0.03656 | 0.33 | 28.9 | 95.64 | 1.67E-18 |
| Fatty acid elongation | KEGG 2021 | 0.04104 | 0.33 | 25.6 | 81.64 | 1.67E-18 |
|  |  |  |  |  |  |  |
| **MEpink correlated with p53 Status** |  |  |  |  |  |  |
| negative regulation of programmed cell death (GO:0043069) | BP 2021 | 0.000321 | 0.03 | 14.9 | 119.5 | 0.000250903 |
| cellular response to growth factor stimulus (GO:0071363) | BP 2021 | 0.0003618 | 0.03 | 25.6 | 202.7 | 1.02E-06 |
| positive regulation of epithelial cell migration (GO:0010634) | BP 2021 | 0.000077948 | 0.03 | 43.7 | 413.5 | 0.015657816 |
| positive regulation of cell-substrate junction organization (GO:0150117) | BP 2021 | 0.0002087 | 0.03 | 113.4 | 961.1 | 0.248485344 |
| TOR signaling (GO:0031929) | BP 2021 | 0.0002455 | 0.03 | 103.9 | 864.1 | 0.248485344 |
| Pancreatic cancer | KEGG 2021 | 0.002096 | 0.08 | 33.6 | 207.4 | 1.31E-28 |
| Fc gamma R-mediated phagocytosis | KEGG 2021 | 0.003386 | 0.08 | 26.2 | 148.8 | 1.31E-28 |
| HIF-1 signaling pathway | KEGG 2021 | 0.004253 | 0.08 | 23.2 | 126.8 | 1.31E-28 |
| Proteoglycans in cancer | KEGG 2021 | 0.01436 | 0.11 | 12.2 | 51.68 | 1.31E-28 |
| Phenylalanine metabolism | KEGG 2021 | 0.0152 | 0.11 | 73.4 | 307.3 | 1.31E-28 |
|  |  |  |  |  |  |  |
| **MEgreen correlated with p53 Status** |  |  |  |  |  |  |
| ribonucleoprotein complex assembly (GO:0022618) | BP 2021 | 0.0002752 | 0.04 | 28 | 229.4 | 9.80E-05 |
| central nervous system neuron axonogenesis (GO:0021955) | BP 2021 | 0.000066276 | 0.02 | 213.6 | 2055 | 0.033360167 |
| negative regulation of protein metabolic process (GO:0051248) | BP 2021 | 0.001102 | 0.06 | 46.9 | 319.4 | 0.033360167 |
| ubiquitin-dependent ERAD pathway (GO:0030433) | BP 2021 | 0.002158 | 0.06 | 33 | 202.5 | 0.033360167 |
| ERAD pathway (GO:0036503) | BP 2021 | 0.003048 | 0.06 | 27.5 | 159.5 | 0.033360167 |
| Protein processing in endoplasmic reticulum | KEGG 2021 | 0.0005381 | 0.01 | 22.1 | 166.5 | 7.10E-12 |
| Ferroptosis | KEGG 2021 | 0.03826 | 0.25 | 27.7 | 90.38 | 7.10E-12 |
| Antigen processing and presentation | KEGG 2021 | 0.07159 | 0.25 | 14.4 | 37.87 | 7.10E-12 |
| IL-17 signaling pathway | KEGG 2021 | 0.08566 | 0.25 | 11.9 | 29.2 | 7.10E-12 |
| Prostate cancer | KEGG 2021 | 0.08827 | 0.25 | 11.5 | 27.93 | 7.10E-12 |
|  |  |  |  |  |  |  |
| **MEdarkred correlated with Platelets** |  |  |  |  |  |  |
| positive regulation of mRNA catabolic process (GO:0061014) | BP 2021 | 4.28E-05 | 0.01685 | 52.08 | 523.9 | 8.24E-15 |
| regulation of actin polymerization or depolymerization (GO:0008064) | BP 2021 | 0.0009197 | 0.05554 | 50.94 | 356.1 | 7.99E-11 |
| gene silencing by miRNA (GO:0035195) | BP 2021 | 0.00112 | 0.05554 | 45.84 | 311.4 | 7.99E-11 |
| post-transcriptional gene silencing by RNA (GO:0035194) | BP 2021 | 0.0004341 | 0.04276 | 76.44 | 591.8 | 7.99E-11 |
| regulation of mRNA stability (GO:0043488) | BP 2021 | 0.001475 | 0.0581 | 14.85 | 96.84 | 8.59E-05 |
| Ribosome | KEGG 2021 | 0.00692 | 0.16 | 18.2 | 90.3 | 7.37E-25 |
| Non-homologous end-joining | KEGG 2021 | 0.01035 | 0.16 | 111 | 507.1 | 7.37E-25 |
| Other glycan degradation | KEGG 2021 | 0.01431 | 0.16 | 78.3 | 332.5 | 7.37E-25 |
| Glycosaminoglycan degradation | KEGG 2021 | 0.0151 | 0.16 | 74 | 310.1 | 7.37E-25 |
| Galactose metabolism | KEGG 2021 | 0.02452 | 0.17 | 44.3 | 164.4 | 7.37E-25 |
|  |  |  |  |  |  |  |
| **MEgreenyellow correlated with Lymphocytes** |  |  |  |  |  |  |
| positive regulation of extracellular matrix assembly (GO:1901203) | BP 2021 | 2.29E-07 | 0 | 383.9 | 5871 | 0.82894069 |
| regulation of anatomical structure morphogenesis (GO:0022603) | BP 2021 | 0.00002872 | 0 | 26.7 | 279.1 | 0.344616273 |
| regulation of basement membrane assembly involved in embryonic body morphogenesis (GO:1904259) | BP 2021 | 0.000020245 | 0 | 493 | 5329 | 1 |
| dense core granule cytoskeletal transport (GO:0099519) | BP 2021 | 0.000042439 | 0 | 295.8 | 2978 | 1 |
| positive regulation of basement membrane assembly involved in embryonic body morphogenesis (GO:1904261) | BP 2021 | 0.000020245 | 0 | 493 | 5329 | 1 |
| Osteoclast differentiation | KEGG 2021 | 0.01452 | 0.29 | 11.8 | 49.77 | 1.01E-18 |
| Pentose phosphate pathway | KEGG 2021 | 0.04263 | 0.29 | 24.6 | 77.49 | 1.01E-18 |
| Galactose metabolism | KEGG 2021 | 0.04402 | 0.29 | 23.7 | 74.14 | 1.01E-18 |
| Fructose and mannose metabolism | KEGG 2021 | 0.04679 | 0.29 | 22.3 | 68.14 | 1.01E-18 |
|  |  |  |  |  |  |  |
| **MEyellow correlated with Lymphocytes** |  |  |  |  |  |  |
| heparin metabolic process (GO:0030202) | BP 2021 | 0.00723 | 0.1437 | 178.3 | 878.8 | 0.043292799 |
| photoperiodism (GO:0009648) | BP 2021 | 0.0007496 | 0.08462 | 56.82 | 408.9 | 9.81E-10 |
| nucleic acid phosphodiester bond hydrolysis (GO:0090305) | BP 2021 | 0.005276 | 0.1437 | 20.19 | 105.9 | 9.81E-10 |
| protein deubiquitination involved in ubiquitin-dependent protein catabolic process (GO:0071947) | BP 2021 | 0.00867 | 0.1437 | 142.6 | 677.1 | 0.043292799 |
| PML body organization (GO:0030578) | BP 2021 | 0.00723 | 0.1437 | 178.3 | 878.8 | 0.043292799 |
| Mismatch repair | KEGG 2021 | 0.03284 | 0.16 | 32.4 | 110.6 | 1.76E-20 |
| Circadian rhythm | KEGG 2021 | 0.04402 | 0.16 | 23.7 | 74.14 | 1.24E-19 |
|  |  |  |  |  |  |  |
| **MEpurple correlated with Lymphocytes** |  |  |  |  |  |  |
| positive regulation of mRNA catabolic process (GO:0061014) | BP 2021 | 0.000034876 | 0.01 | 56.1 | 575.7 | 1.67E-18 |
| positive regulation of nuclear-transcribed mRNA poly(A) tail shortening (GO:0060213) | BP 2021 | 0.0001568 | 0.03 | 134.4 | 1178 | 1.67E-18 |
| regulation of nuclear-transcribed mRNA poly(A) tail shortening (GO:0060211) | BP 2021 | 0.0002107 | 0.03 | 113.7 | 962.7 | 1.67E-18 |
| post-transcriptional gene silencing by RNA (GO:0035194) | BP 2021 | 0.0003795 | 0.04 | 82.1 | 646.8 | 1.67E-18 |
| regulation of mRNA catabolic process (GO:0061013) | BP 2021 | 0.0007206 | 0.05 | 19.3 | 139.3 | 1.67E-18 |
| Platelet activation | KEGG 2021 | 0.01388 | 0.31 | 12.1 | 51.55 | 2.26E-19 |
| Vitamin digestion and absorption | KEGG 2021 | 0.03425 | 0.31 | 31 | 104.5 | 2.26E-19 |
| Glycosylphosphatidylinositol (GPI)-anchor biosynthesis | KEGG 2021 | 0.03705 | 0.31 | 28.5 | 93.9 | 2.26E-19 |
| Fatty acid elongation | KEGG 2021 | 0.03845 | 0.31 | 27.4 | 89.27 | 1.67E-18 |
| Butanoate metabolism | KEGG 2021 | 0.03984 | 0.31 | 26.4 | 85.02 | 1.67E-18 |
|  |  |  |  |  |  |  |
| **MEsalmon correlated with Hemoglobin** |  |  |  |  |  |  |
| regulation of lymphocyte migration (GO:2000401) | BP 2021 | 0.003296 | 0.02154 | 399.7 | 2284 | 4.38E-16 |
| T-helper cell lineage commitment (GO:0002295) | BP 2021 | 0.002398 | 0.02154 | 571.1 | 3445 | 4.38E-16 |
| negative regulation of production of miRNAs involved in gene silencing by miRNA (GO:1903799) | BP 2021 | 0.003595 | 0.02154 | 363.3 | 2045 | 4.38E-16 |
| negative regulation of purine nucleotide metabolic process (GO:1900543) | BP 2021 | 0.002997 | 0.02154 | 444.1 | 2580 | 4.38E-16 |
| negative regulation of glycolytic process (GO:0045820) | BP 2021 | 0.002997 | 0.02154 | 444.1 | 2580 | 4.38E-16 |
| Mannose type O-glycan biosynthesis | KEGG 2021 | 0.006881 | 0.03 | 181.6 | 904 | 4.38E-16 |
| Thyroid hormone signaling pathway | KEGG 2021 | 0.03576 | 0.06 | 33.1 | 110.3 | 4.38E-16 |
| Cell adhesion molecules | KEGG 2021 | 0.04359 | 0.06 | 27 | 84.6 | 4.38E-16 |
| Wnt signaling pathway | KEGG 2021 | 0.04878 | 0.06 | 24 | 72.59 | 4.38E-16 |
| Transcriptional misregulation in cancer | KEGG 2021 | 0.05624 | 0.06 | 20.7 | 59.68 | 3.06E-15 |
|  |  |  |  |  |  |  |
| **MEbrown correlated with Hemoglobin** |  |  |  |  |  |  |
| supramolecular fiber organization (GO:0097435) | BP 2021 | 0.002085 | 0.06 | 14.1 | 87.08 | 0.230438739 |
| ephrin receptor signaling pathway (GO:0048013) | BP 2021 | 0.001684 | 0.06 | 38.3 | 244.5 | 0.1114336 |
| peptidyl-lysine modification (GO:0018205) | BP 2021 | 0.002793 | 0.06 | 29.4 | 172.9 | 0.111433596 |
| isopentenyl diphosphate biosynthetic process (GO:0009240) | BP 2021 | 0.003745 | 0.06 | 356.8 | 1994 | 0.046980968 |
| response to mitochondrial depolarisation (GO:0098780) | BP 2021 | 0.003745 | 0.06 | 356.8 | 1994 | 0.046980968 |
| Amyotrophic lateral sclerosis | KEGG 2021 | 0.002312 | 0.16 | 13.6 | 82.49 | 3.71E-113 |
| Other glycan degradation | KEGG 2021 | 0.01342 | 0.16 | 83.9 | 361.7 | 6.96E-115 |
| cAMP signaling pathway | KEGG 2021 | 0.01111 | 0.16 | 14.2 | 63.96 | 5.10E-114 |
| Salmonella infection | KEGG 2021 | 0.01457 | 0.16 | 12.3 | 51.99 | 5.10E-114 |
| Pathways of neurodegeneration | KEGG 2021 | 0.004896 | 0.16 | 10.3 | 54.98 | 3.71E-113 |
|  |  |  |  |  |  |  |
| **MEsalmon correlated with WBC** |  |  |  |  |  |  |
| regulation of cysteine-type endopeptidase activity involved in apoptotic process (GO:0043281) | BP 2021 | 1.50E-05 | 0.01016 | 18.17 | 201.9 | 6.57E-11 |
| regulation of transcription by RNA polymerase II (GO:0006357) | BP 2021 | 0.001287 | 0.09344 | 2.6 | 17.3 | 0 |
| negative regulation of glycolytic process (GO:0045820) | BP 2021 | 0.0005336 | 0.0793 | 73.24 | 551.9 | 7.28E-16 |
| negative regulation of cysteine-type endopeptidase activity (GO:2000117) | BP 2021 | 3.54E-05 | 0.01016 | 24.59 | 252 | 6.49E-07 |
| negative regulation of purine nucleotide metabolic process (GO:1900543) | BP 2021 | 0.0005336 | 0.0793 | 73.24 | 551.9 | 0.070298627 |
| Thyroid hormone signaling pathway | KEGG 2021 | 0.0008589 | 0.09 | 10.3 | 72.46 | 1.26E-13 |
| Mitophagy | KEGG 2021 | 0.001749 | 0.1 | 13.7 | 86.88 | 2.02E-14 |
| Pentose phosphate pathway | KEGG 2021 | 0.00493 | 0.14 | 20.9 | 111.1 | 3.06E-15 |
| Galactose metabolism | KEGG 2021 | 0.005259 | 0.14 | 20.2 | 105.9 | 3.06E-15 |
| Bladder cancer | KEGG 2021 | 0.009067 | 0.2 | 15 | 70.55 | 3.06E-15 |
|  |  |  |  |  |  |  |
| **MEmagenta correlated with WBC** |  |  |  |  |  |  |
| regulation of transcription by RNA polymerase II (GO:0006357) | BP 2021 | 2.13E-05 | 0.01278 | 2.177 | 23.42 | 4.38E-08 |
| positive regulation of transcription, DNA-templated (GO:0045893) | BP 2021 | 2.82E-05 | 0.01278 | 2.568 | 26.9 | 2.18E-06 |
| negative regulation of transcription, DNA-templated (GO:0045892) | BP 2021 | 3.88E-05 | 0.01278 | 2.723 | 27.66 | 6.92E-05 |
| regulation of transforming growth factor beta receptor signaling pathway (GO:0017015) | BP 2021 | 8.51E-06 | 0.01278 | 8.742 | 102 | 0.759541299 |
| regulation of macromolecule metabolic process (GO:0060255) | BP 2021 | 4.09E-05 | 0.01278 | 5.319 | 53.74 | 0.922159917 |
| Adherens junction | KEGG 2021 | 0.005995 | 0.55 | 5.9 | 30.13 | N/A |
| Protein processing in endoplasmic reticulum | KEGG 2021 | 0.008293 | 0.55 | 3.6 | 17.28 | N/A |
| Notch signaling pathway | KEGG 2021 | 0.02238 | 0.55 | 5.3 | 19.99 | N/A |
| Hippo signaling pathway | KEGG 2021 | 0.02601 | 0.55 | 3.1 | 11.4 | N/A |
| Wnt signaling pathway | KEGG 2021 | 0.02785 | 0.55 | 3.1 | 10.97 | N/A |
|  |  |  |  |  |  |  |
| **MEgreen correlated with WBC** |  |  |  |  |  |  |
| regulation of protein ubiquitination (GO:0031396) | BP 2021 | 0.002145 | 0.1332 | 12.77 | 78.44 | 1.38E-28 |
| rRNA methylation (GO:0031167) | BP 2021 | 0.001212 | 0.09676 | 44.3 | 297.5 | 9.66E-12 |
| mRNA methylation (GO:0080009) | BP 2021 | 0.0005566 | 0.07778 | 68.17 | 510.9 | 9.66E-12 |
| rRNA base methylation (GO:0070475) | BP 2021 | 0.00015 | 0.07727 | 147.8 | 1301 | 9.66E-12 |
| mRNA modification (GO:0016556) | BP 2021 | 0.001212 | 0.09676 | 44.3 | 297.5 | 9.66E-12 |
| Protein processing in endoplasmic reticulum | KEGG 2021 | 0.007559 | 0.3 | 8 | 39.23 | 2.23E-09 |
| Vitamin B6 metabolism | KEGG 2021 | 0.01402 | 0.3 | 86.7 | 370.1 | 5.24E-11 |
| Fluid shear stress and atherosclerosis | KEGG 2021 | 0.0423 | 0.45 | 6.4 | 20.33 | 3.56E-10 |
|  |  |  |  |  |  |  |
| **MEpurple correlated with WBC** |  |  |  |  |  |  |
| negative regulation of stem cell differentiation (GO:2000737) | BP 2021 | 0.001054 | 0.08039 | 49.6 | 340 | 1.17E-17 |
| positive regulation of mRNA catabolic process (GO:0061014) | BP 2021 | 6.28E-07 | 0 | 36.4 | 520.2 | 9.74E-14 |
| regulation of mRNA stability (GO:0043488) | BP 2021 | 0.000018162 | 0 | 12.3 | 134.1 | 9.74E-14 |
| regulation of mRNA catabolic process (GO:0061013) | BP 2021 | 6.50E-06 | 0 | 14.9 | 177.4 | 7.86E-17 |
| mRNA destabilization (GO:0061157) | BP 2021 | 0.0003888 | 0.06 | 23.7 | 185.9 | 7.86E-17 |
| Salmonella infection | KEGG 2021 | 0.01431 | 0.65 | 4.5 | 19.22 | 5.02E-16 |
| TGF-beta signaling pathway | KEGG 2021 | 0.04861 | 0.65 | 5.9 | 17.86 | 1.17E-17 |
|  |  |  |  |  |  |  |
| **MEblue correlated with Lobe Location** |  |  |  |  |  |  |
| peptidyl-lysine dimethylation (GO:0018027) | BP 2021 | 0.0006527 | 0.13 | 61.7 | 452.8 | 6.56E-01 |
| protein-containing complex assembly (GO:0065003) | BP 2021 | 0.001945 | 0.13 | 8.3 | 51.95 | 1.34E-10 |
| beta-catenin-TCF complex assembly (GO:1904837) | BP 2021 | 0.00153 | 0.13 | 38.9 | 251.9 | 1.57E-03 |
| peptidyl-lysine trimethylation (GO:0018023) | BP 2021 | 0.001637 | 0.13 | 37.5 | 240.3 | 6.56E-01 |
| positive regulation of cellular component movement (GO:0051272) | BP 2021 | 0.001326 | 0.13 | 42 | 278.1 | N/A |
| FoxO signaling pathway | KEGG 2021 | 0.002273 | 0.06 | 12.6 | 76.46 | 8.87E-116 |
| Autophagy | KEGG 2021 | 0.002581 | 0.06 | 12 | 71.49 | 1.23E-116 |
| Lysine degradation | KEGG 2021 | 0.007052 | 0.09 | 17.2 | 85.06 | 1.69E-117 |
| Adipocytokine signaling pathway | KEGG 2021 | 0.008408 | 0.09 | 15.6 | 74.67 | 1.69E-117 |
| Longevity regulating pathway | KEGG 2021 | 0.01771 | 0.15 | 10.5 | 42.16 | 1.69E-117 |
|  |  |  |  |  |  |  |
| **MEdarkgreen correlated with Lobe Location** |  |  |  |  |  |  |
| positive regulation of ubiquitin-dependent protein catabolic process (GO:2000060) | BP 2021 | 0.0005275 | 0.08631 | 21.21 | 160.1 | 0.003718516 |
| positive regulation of proteasomal ubiquitin-dependent protein catabolic process (GO:0032436) | BP 2021 | 0.0005275 | 0.08631 | 21.21 | 160.1 | 0.003718516 |
| positive regulation of proteasomal protein catabolic process (GO:1901800) | BP 2021 | 0.000676 | 0.08631 | 19.42 | 141.7 | 0.003718516 |
| regulation of proteasomal ubiquitin-dependent protein catabolic process (GO:0032434) | BP 2021 | 0.001109 | 0.08705 | 16.27 | 110.7 | 0.003718516 |
| protein quality control for misfolded or incompletely synthesized proteins (GO:0006515) | BP 2021 | 0.001136 | 0.08705 | 45.62 | 309.3 | 1 |
| Homologous recombination | KEGG 2021 | 0.003044 | 0.13 | 26.9 | 155.8 | 2.04E-131 |
| Wnt signaling pathway | KEGG 2021 | 0.04348 | 0.3 | 6.4 | 19.92 | 2.04E-131 |
| Hepatocellular carcinoma | KEGG 2021 | 0.04443 | 0.3 | 6.3 | 19.54 | 1.50E-130 |
| Protein export | KEGG 2021 | 0.04503 | 0.3 | 23.2 | 72.05 | 1.50E-130 |
| Vitamin digestion and absorption | KEGG 2021 | 0.04694 | 0.3 | 22.2 | 67.99 | 1.50E-130 |
|  |  |  |  |  |  |  |
| **MEgreen correlated with Lobe Location** |  |  |  |  |  |  |
| mRNA methylation (GO:0080009) | BP 2021 | 0.0001962 | 0.01845 | 118.1 | 1008 | 5.17E-11 |
| rRNA base methylation (GO:0070475) | BP 2021 | 5.26E-05 | 0.01499 | 256 | 2522 | 5.17E-11 |
| mRNA modification (GO:0016556) | BP 2021 | 0.0004291 | 0.02689 | 76.74 | 595 | 5.17E-11 |
| central nervous system neuron axonogenesis (GO:0021955) | BP 2021 | 0.000146 | 0.0183 | 139.6 | 1233 | 5.17E-11 |
| RNA methylation (GO:0001510) | BP 2021 | 7.97E-05 | 0.01499 | 41.93 | 395.7 | 7.76E-27 |
| Protein processing in endoplasmic reticulum | KEGG 2021 | 0.001719 | 0.05 | 14.2 | 90.05 | 2.23E-09 |
| Vitamin B6 metabolism | KEGG 2021 | 0.008372 | 0.13 | 147.9 | 707.4 | 5.24E-11 |
| Fructose and mannose metabolism | KEGG 2021 | 0.04522 | 0.36 | 23.1 | 71.46 | 5.24E-11 |
|  |  |  |  |  |  |  |
| **MEgreenyellow correlated with Lobe Location** |  |  |  |  |  |  |
| cation transmembrane transport (GO:0098655) | BP 2021 | 0.001724 | 0.09854 | 36.5 | 232.3 | 7.08E-18 |
| negative regulation of DNA metabolic process (GO:0051053) | BP 2021 | 0.001156 | 0.09854 | 45.11 | 305.1 | 7.08E-18 |
| RNA catabolic process (GO:0006401) | BP 2021 | 0.002134 | 0.09854 | 32.61 | 200.5 | 7.08E-18 |
| nucleobase-containing compound catabolic process (GO:0034655) | BP 2021 | 0.001498 | 0.09854 | 39.32 | 255.7 | 7.08E-18 |
| regulation of ubiquitin-protein transferase activity (GO:0051438) | BP 2021 | 0.0007496 | 0.09854 | 56.82 | 408.9 | 7.08E-18 |
| Ras signaling pathway | KEGG 2021 | 0.04156 | 0.3 | 6.6 | 21 | 7.08E-18 |
| SNARE interactions in vesicular transport | KEGG 2021 | 0.04522 | 0.3 | 23.1 | 71.46 | 1.01E-18 |
| DNA replication | KEGG 2021 | 0.04923 | 0.3 | 21.1 | 63.53 | 1.01E-18 |
|  |  |  |  |  |  |  |
| **MElightcyan correlated with Lobe Location** |  |  |  |  |  |  |
| regulation of type I interferon production (GO:0032479) | BP 2021 | 0.0002575 | 0.02403 | 27.75 | 229.3 | 0.221905087 |
| mammary gland development (GO:0030879) | BP 2021 | 0.0005118 | 0.03583 | 69.76 | 528.6 | 0.755420189 |
| positive regulation of interferon-beta production (GO:0032728) | BP 2021 | 0.001156 | 0.04747 | 45.11 | 305.1 | 0.755420189 |
| lactation (GO:0007595) | BP 2021 | 8.45E-05 | 0.02365 | 192 | 1800 | 0.755420189 |
| body fluid secretion (GO:0007589) | BP 2021 | 0.0002241 | 0.02403 | 109.7 | 921.5 | 0.755420189 |
| Glycerophospholipid metabolism | KEGG 2021 | 0.008268 | 0.14 | 15.9 | 76.37 | 3.38E-57 |
| Phosphonate and phosphinate metabolism | KEGG 2021 | 0.008372 | 0.14 | 147.9 | 707.4 | 4.65E-58 |
| TNF signaling pathway | KEGG 2021 | 0.01068 | 0.14 | 13.9 | 63.04 | 3.38E-57 |
| Growth hormone synthesis, secretion and action | KEGG 2021 | 0.012 | 0.14 | 13.1 | 57.74 | 3.38E-57 |
| Osteoclast differentiation | KEGG 2021 | 0.01358 | 0.14 | 12.2 | 52.51 | 3.38E-57 |
|  |  |  |  |  |  |  |
| **MEred correlated with Lobe Location** |  |  |  |  |  |  |
| glycosaminoglycan catabolic process (GO:0006027) | BP 2021 | 0.002778 | 0.1076 | 28.37 | 167 | 2.14E-10 |
| R-loop disassembly (GO:0062176) | BP 2021 | 0.006981 | 0.1076 | 184.9 | 917.9 | 3.15E-11 |
| radial glial cell differentiation (GO:0060019) | BP 2021 | 0.008372 | 0.1076 | 147.9 | 707.4 | 3.15E-11 |
| positive regulation of cellular component biogenesis (GO:0044089) | BP 2021 | 0.00732 | 0.1076 | 16.99 | 83.56 | 2.14E-10 |
| glycosaminoglycan metabolic process (GO:0030203) | BP 2021 | 5.81E-05 | 0.01883 | 46.87 | 457.2 | 1.34E-09 |

**ME**: module eigengene; **GO**: Gene Ontology; **BP**: Biological Process; **KEGG**: Kyoto Encyclopedia of Genes and Genome.
