# Supplementary figures and images for: Epitranscriptomic analysis reveals clinical and molecular signatures in glioblastoma
Source: Acta Neuropathol Commun. 2025 Apr 11;13:74. doi: 10.1186/s40478-025-01966-5 (PMC11987271; doi:10.1186/s40478-025-01966-5)

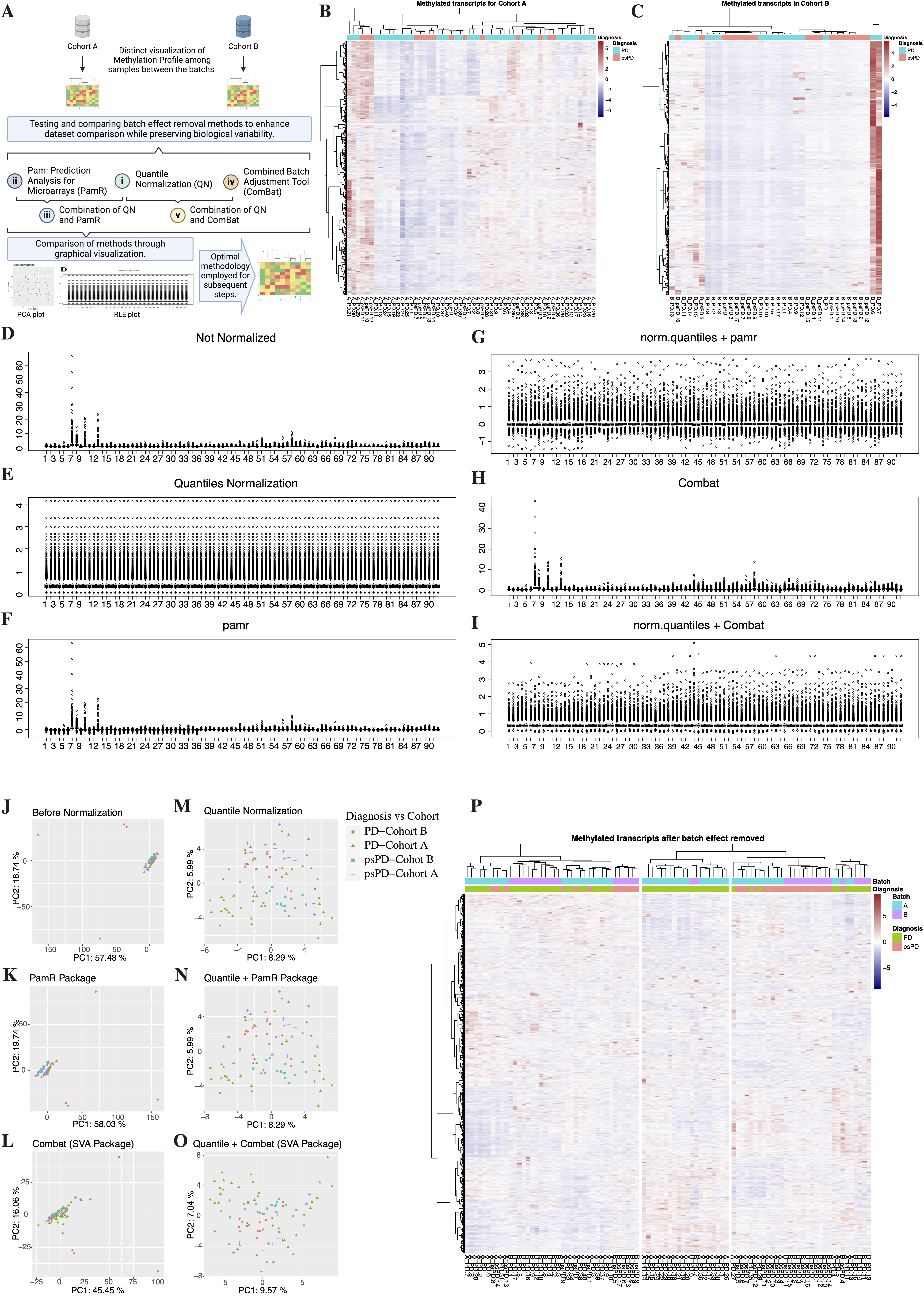

Supplement: Supplementary file 2 — Supplementary Material 2 [file 40478_2025_1966_MOESM2_ESM.png]

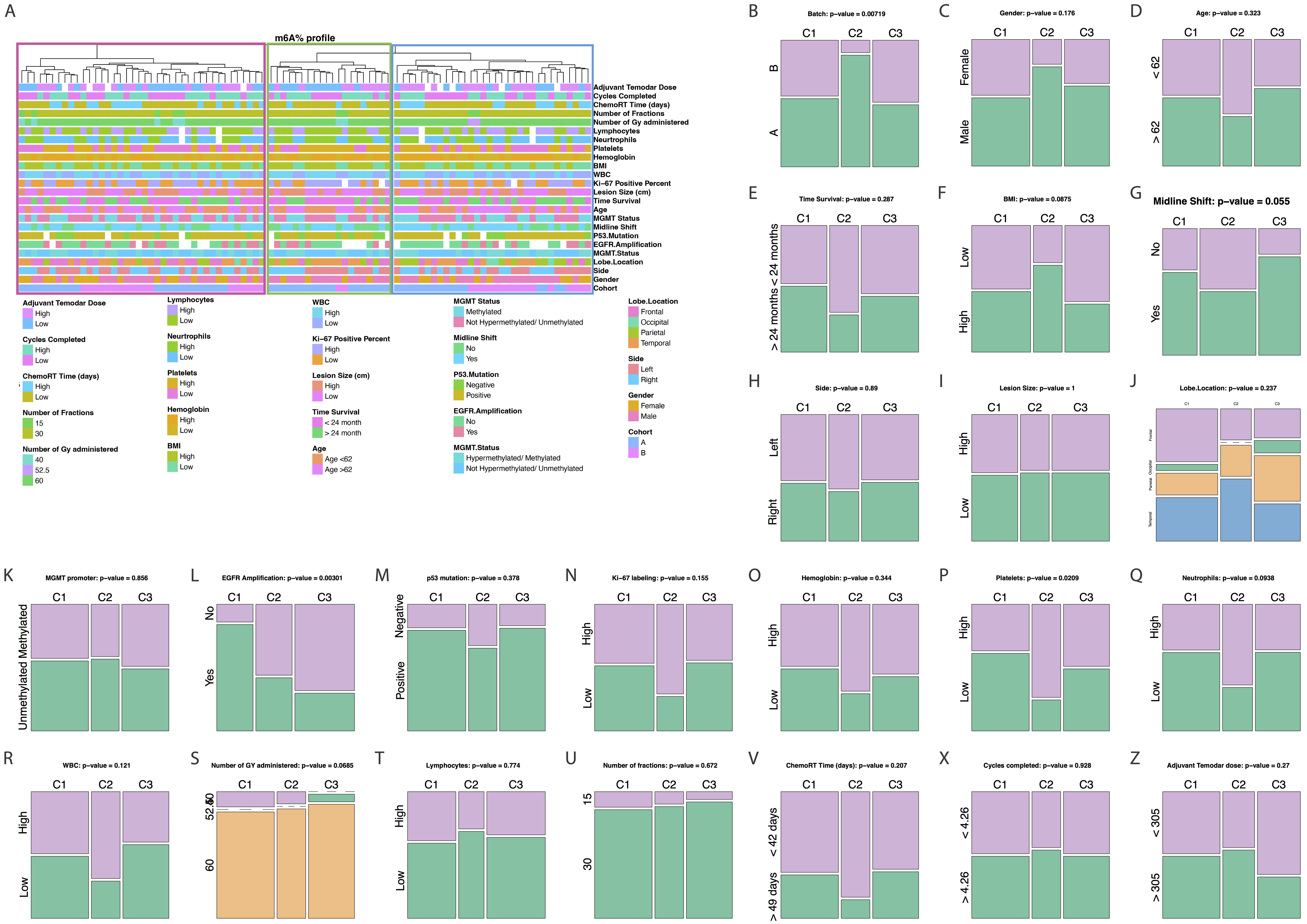

Supplement: Supplementary file 3 — Supplementary Material 3 [file 40478_2025_1966_MOESM3_ESM.png]

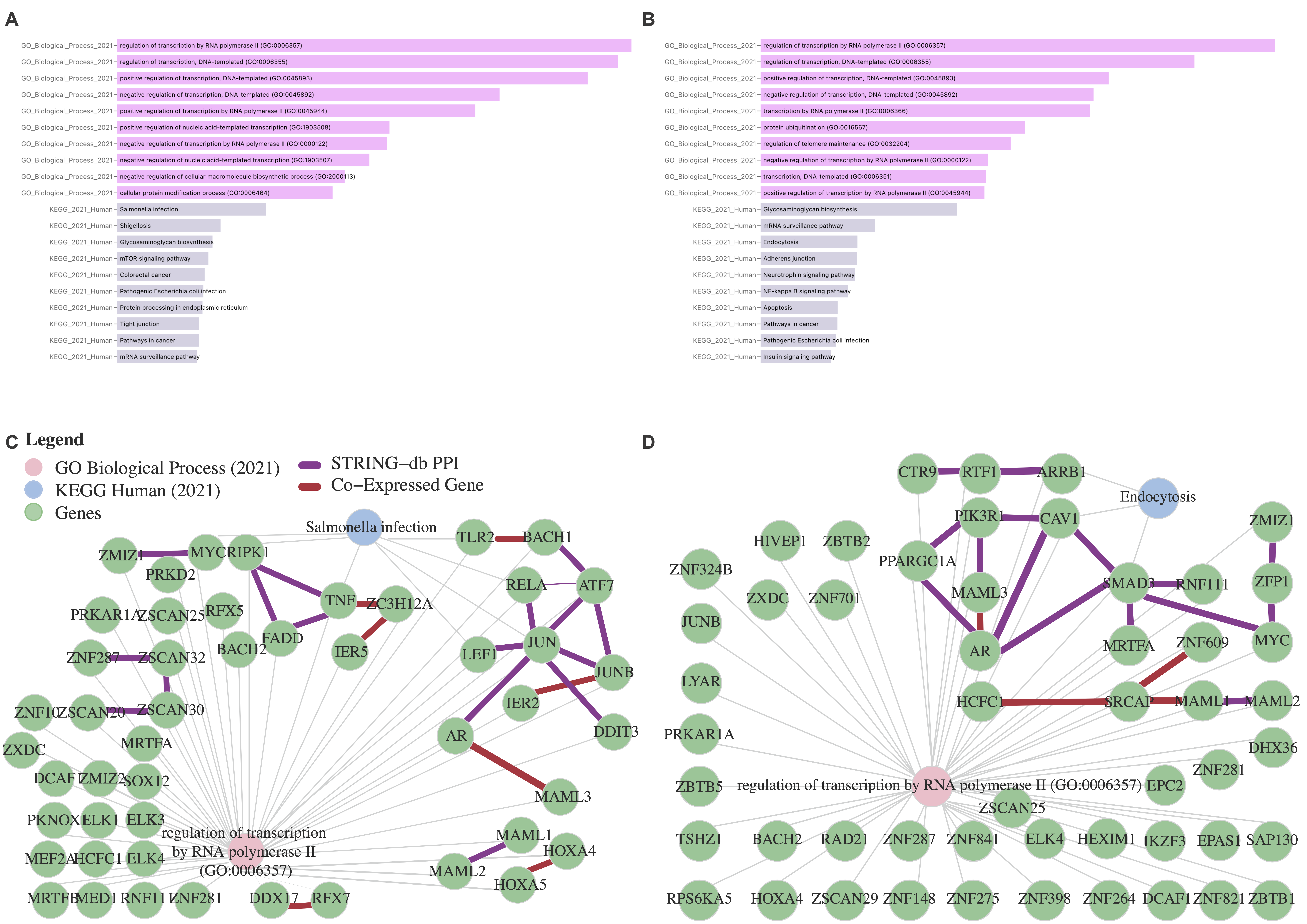

Supplement: Supplementary file 4 — Supplementary Material 4 [file 40478_2025_1966_MOESM4_ESM.png]

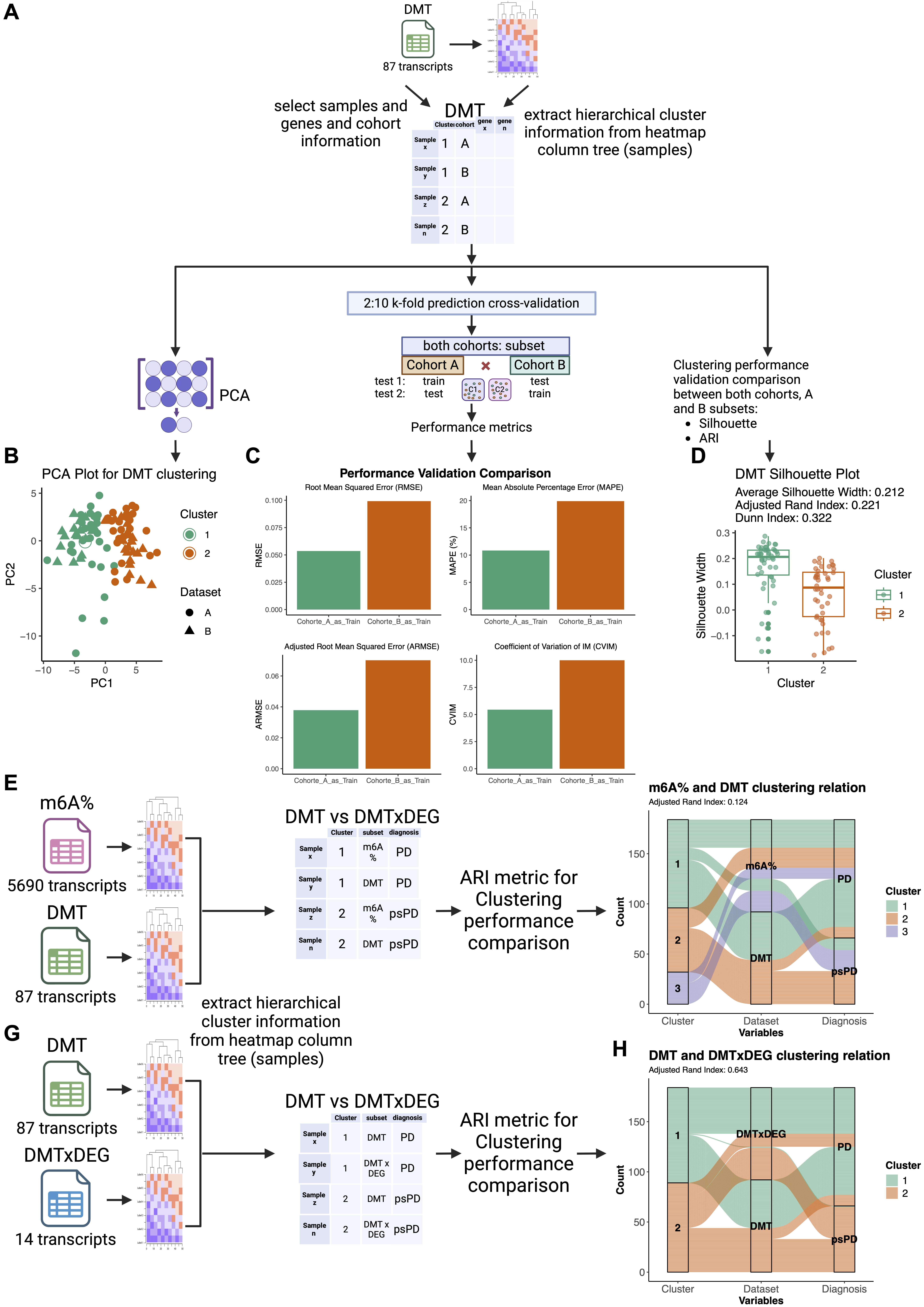

Supplement: Supplementary file 5 — Supplementary Material 5 [file 40478_2025_1966_MOESM5_ESM.png]
