# Supplementary material for: Epitranscriptomic analysis reveals clinical and molecular signatures in glioblastoma
Source: Acta Neuropathol Commun. 2025 Apr 11;13:74. doi: 10.1186/s40478-025-01966-5 (PMC11987271; doi:10.1186/s40478-025-01966-5)
Supplement: Supplementary file 6 — Supplementary Material 6 [file 40478_2025_1966_MOESM6_ESM.pdf]

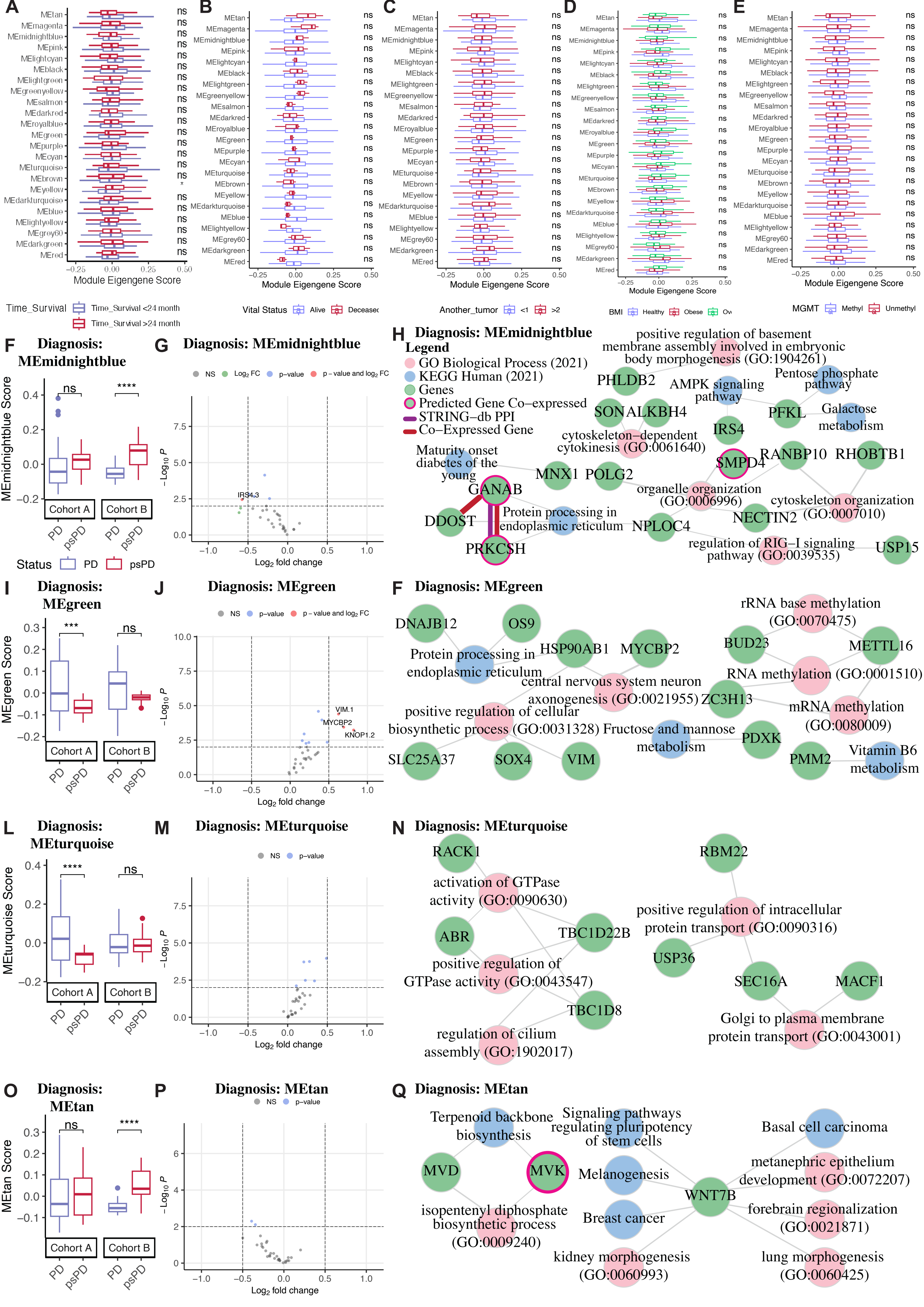

A
MEtan
MEmagenta
MEmidnightblue
MEpink
MElightcyan
MEblack
MElightgreen
MEgreenyellow
MEsalmon
MEdarkred
MEroyalblue
MEgreen
MEpurple
MEcyan
MEturquoise
MEbrown
MEyellow
MEdarkturquoise
MEblue
MElightyellow
MEgrey60
MEdarkgreen
MEred
Module Eigengene Score
Time_Survival
Time_Survival <24 month
Time_Survival >24 month
B
Vital Status
Alive
Deceased
C
Another_tumor
<1
>2
D
BMI
Healthy
Obese
E
MGMT
Methyl
Unmethyl
F
Diagnosis: MEmidnightblue
MEmidnightblue Score
Cohort A
Cohort B
PD
psPD
Status
G
Diagnosis: MEmidnightblue
NS
Log2 FC
p-value
p-value and log2 FC
IRS4.3
Log2 fold change
H
Diagnosis: MEmidnightblue
Legend
GO Biological Process (2021)
KEGG Human (2021)
Genes
Predicted Gene Co-expressed
STRING-db PPI
Co-Expressed Gene
positive regulation of basement membrane assembly involved in embryonic body morphogenesis (GO:1904261)
PHLDB2
AMPK signaling pathway
Pentose phosphate pathway
SON
ALKBH4
PFKL
Galactose metabolism
IRS4
cytoskeleton-dependent cytokinesis (GO:0061640)
Maturity onset diabetes of the young
SMPD4
RANBP10
RHOBTB1
MNX1
POLG2
GANAB
organelle organization (GO:0006996)
cytoskeleton organization (GO:0007010)
DDOST
Protein processing in endoplasmic reticulum
NPLOC4
NECTIN2
PRKCSH
regulation of RIG-I signaling pathway (GO:0039535)
USP15
I
Diagnosis: MEgreen
MEgreen Score
J
Diagnosis: MEgreen
VIM.1
MYCBP2
KNOP1.2
F
Diagnosis: MEgreen
DNAJB12
OS9
Protein processing in endoplasmic reticulum
HSP90AB1
MYCBP2
rRNA base methylation (GO:0070475)
BUD23
METTL16
RNA methylation (GO:0001510)
central nervous system neuron axonogenesis (GO:0021955)
ZC3H13
positive regulation of cellular biosynthetic process (GO:0031328)
mRNA methylation (GO:0080009)
Fructose and mannose metabolism
PDXK
SLC25A37
SOX4
VIM
PMM2
Vitamin B6 metabolism
L
Diagnosis: MEturquoise
MEturquoise Score
M
Diagnosis: MEturquoise
N
Diagnosis: MEturquoise
RACK1
RBM22
activation of GTPase activity (GO:0090630)
positive regulation of intracellular protein transport (GO:0090316)
ABR
TBC1D22B
USP36
positive regulation of GTPase activity (GO:0043547)
SEC16A
MACF1
TBC1D8
Golgi to plasma membrane protein transport (GO:0043001)
regulation of cilium assembly (GO:1902017)
O
Diagnosis: MEtan
MEtan Score
P
Diagnosis: MEtan
Q
Diagnosis: MEtan
Terpenoid backbone biosynthesis
Signaling pathways regulating pluripotency of stem cells
Basal cell carcinoma
MVD
MVK
Melanogenesis
metanephric epithelium development (GO:0072207)
WNT7B
isopentenyl diphosphate biosynthetic process (GO:0009240)
Breast cancer
forebrain regionalization (GO:0021871)
kidney morphogenesis (GO:0060993)
lung morphogenesis (GO:0060425)

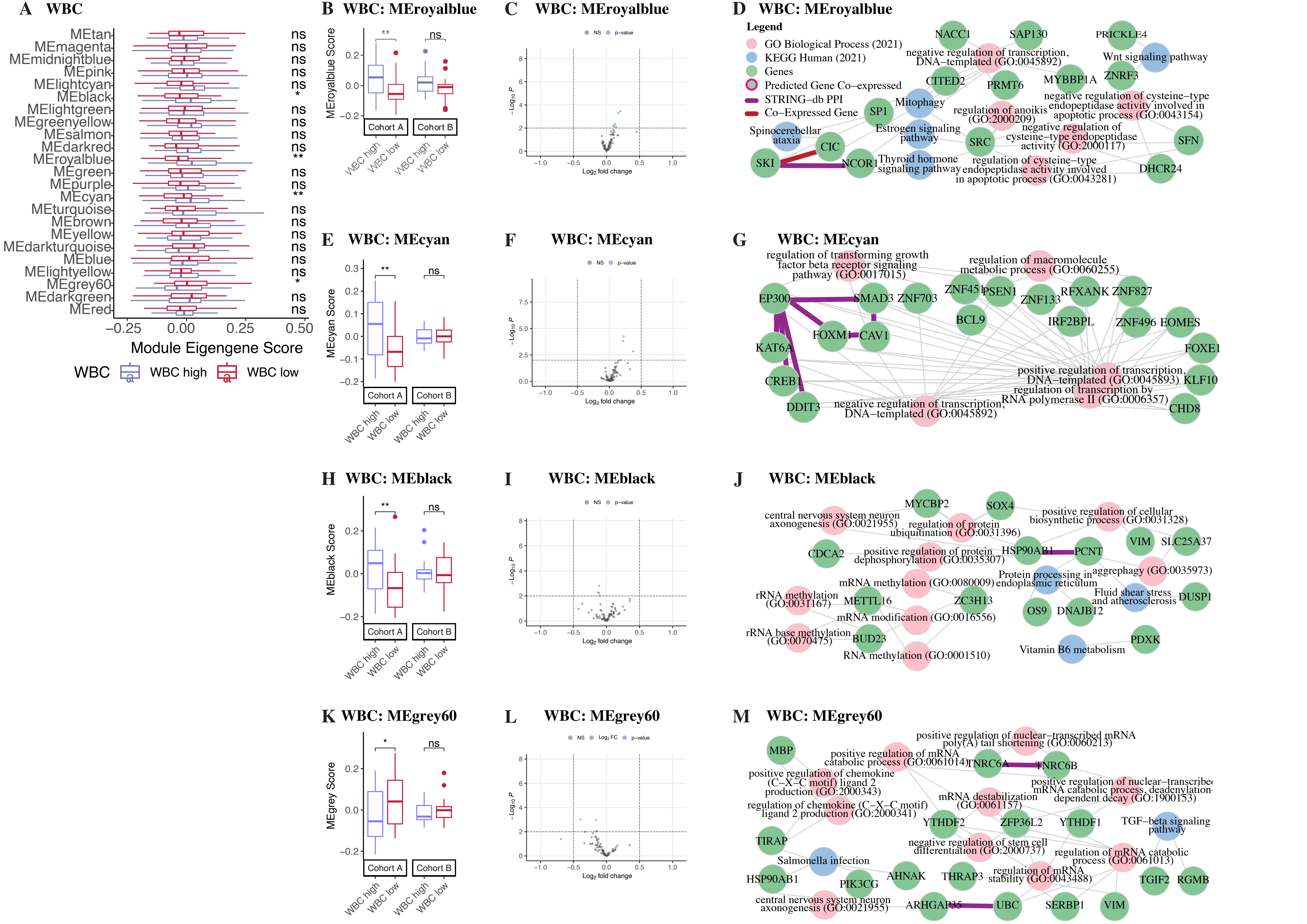

A WBC
B WBC: MEroyalblue
C WBC: MEroyalblue
D WBC: MEroyalblue
E WBC: MEcyan
F WBC: MEcyan
G WBC: MEcyan
H WBC: MEblack
I WBC: MEblack
J WBC: MEblack
K WBC: MEgrey60
L WBC: MEgrey60
M WBC: MEgrey60
Module Eigengene Score
WBC high
WBC low
Cohort A
Cohort B
Legend
GO Biological Process (2021)
KEGG Human (2021)
Genes
Predicted Gene Co-expressed
STRING-db PPI
Co-Expressed Gene

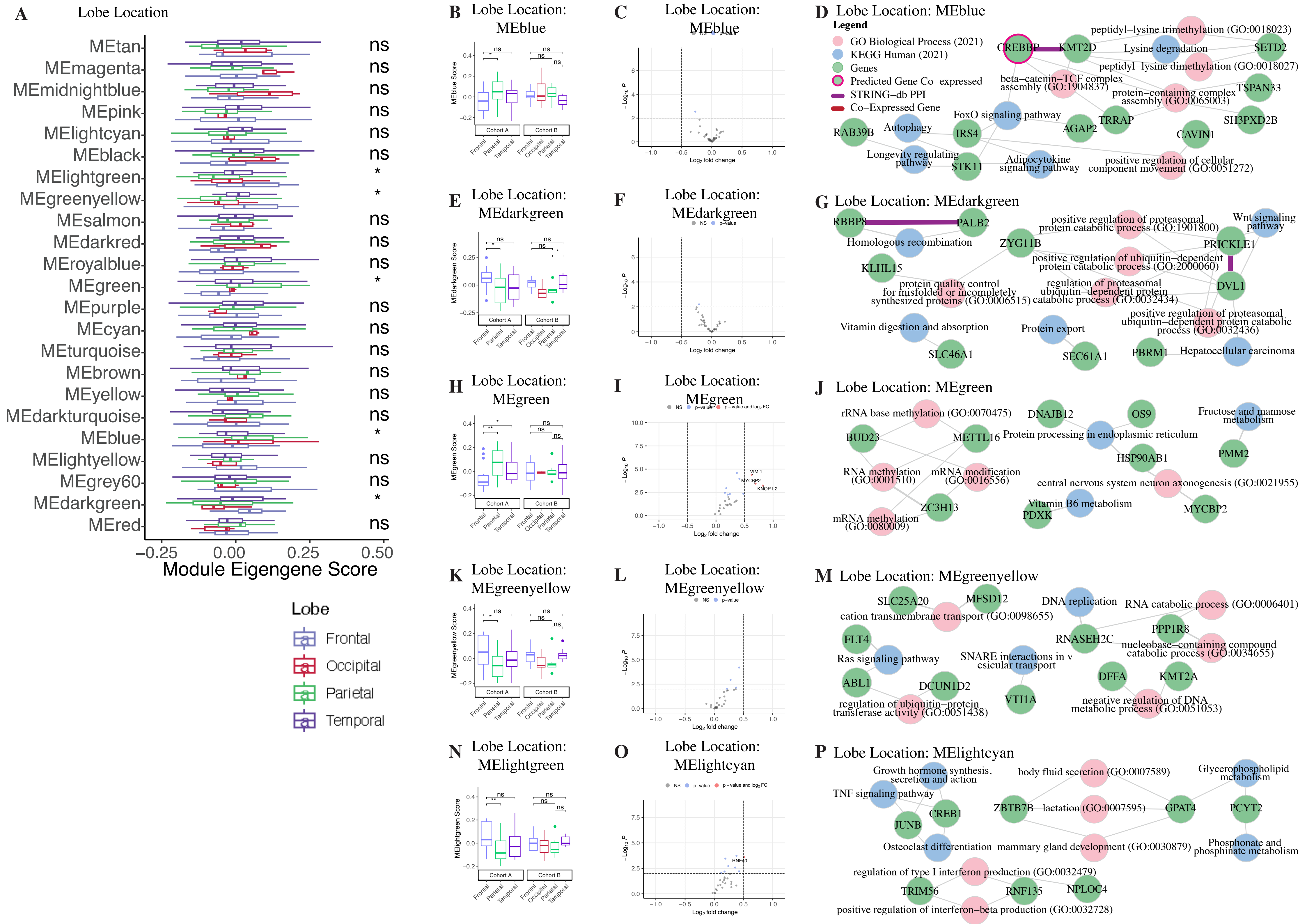
A Lobe Location
MEtan ns
MEmagenta ns
MEmidnightblue ns
MEpink ns
MElightcyan ns
MEblack ns
MElightgreen *
MEgreenyellow *
MEsalmon ns
MEdarkred ns
MEroyalblue ns
MEgreen *
MEpurple ns
MEcyan ns
MEturquoise ns
MEbrown ns
MEyellow ns
MEdarkturquoise ns
MEblue *
MElightyellow ns
MEgrey60 ns
MEdarkgreen *
MEred ns
−0.25 0.00 0.25 0.50
Module Eigengene Score
Lobe
Frontal
Occipital
Parietal
Temporal
B Lobe Location: MEblue
C Lobe Location: MEblue
D Lobe Location: MEblue
Legend
GO Biological Process (2021)
KEGG Human (2021)
Genes
Predicted Gene Co−expressed
STRING-db PPI
Co−Expressed Gene
CREBBP
KMT2D
peptidyl−lysine trimethylation (GO:0018023)
Lysine degradation
SETD2
peptidyl−lysine dimethylation (GO:0018027)
beta−catenin−TCF complex assembly (GO:1904837)
protein−containing complex assembly (GO:0065003)
TSPAN33
FoxO signaling pathway
AGAP2
TRRAP
SH3PXD2B
RAB39B
Autophagy
IRS4
CAVIN1
Longevity regulating pathway
STK11
Adipocytokine signaling pathway
positive regulation of cellular component movement (GO:0051272)
E Lobe Location: MEdarkgreen
F Lobe Location: MEdarkgreen
G Lobe Location: MEdarkgreen
RBBP8
PALB2
Homologous recombination
ZYG11B
positive regulation of proteasomal protein catabolic process (GO:1901800)
Wnt signaling pathway
PRICKLE1
positive regulation of ubiquitin−dependent protein catabolic process (GO:2000060)
KLHL15
protein quality control for misfolded or incompletely synthesized proteins (GO:0006515)
regulation of proteasomal ubiquitin−dependent protein catabolic process (GO:0032434)
DVL1
positive regulation of proteasomal ubiquitin−dependent protein catabolic process (GO:0032436)
Vitamin digestion and absorption
Protein export
SLC46A1
SEC61A1
PBRM1
Hepatocellular carcinoma
H Lobe Location: MEgreen
I Lobe Location: MEgreen
VIM.1
MYCBP2
KNOP1.2
J Lobe Location: MEgreen
rRNA base methylation (GO:0070475)
DNAJB12
OS9
Fructose and mannose metabolism
BUD23
METTL16
Protein processing in endoplasmic reticulum
HSP90AB1
PMM2
RNA methylation (GO:0001510)
mRNA modification (GO:0016556)
central nervous system neuron axonogenesis (GO:0021955)
ZC3H13
Vitamin B6 metabolism
PDXK
MYCBP2
mRNA methylation (GO:0080009)
K Lobe Location: MEgreenyellow
L Lobe Location: MEgreenyellow
M Lobe Location: MEgreenyellow
SLC25A20
MFSD12
DNA replication
RNA catabolic process (GO:0006401)
cation transmembrane transport (GO:0098655)
PPP1R8
FLT4
RNASEH2C
nucleobase−containing compound catabolic process (GO:0034655)
Ras signaling pathway
SNARE interactions in vesicular transport
ABL1
DCUN1D2
DFFA
KMT2A
regulation of ubiquitin−protein transferase activity (GO:0051438)
VTI1A
negative regulation of DNA metabolic process (GO:0051053)
N Lobe Location: MElightgreen
O Lobe Location: MElightcyan
RNF40
P Lobe Location: MElightcyan
Growth hormone synthesis, secretion and action
body fluid secretion (GO:0007589)
Glycerophospholipid metabolism
TNF signaling pathway
CREB1
ZBTB7B
lactation (GO:0007595)
GPAT4
PCYT2
JUNB
Osteoclast differentiation
mammary gland development (GO:0030879)
Phosphonate and phosphinate metabolism
regulation of type I interferon production (GO:0032479)
TRIM56
RNF135
NPLOC4
positive regulation of interferon−beta production (GO:0032728)
Cohort A
Cohort B
Frontal
Occipital
Parietal
Temporal
Log2 fold change
NS p−value p − value and log2 FC

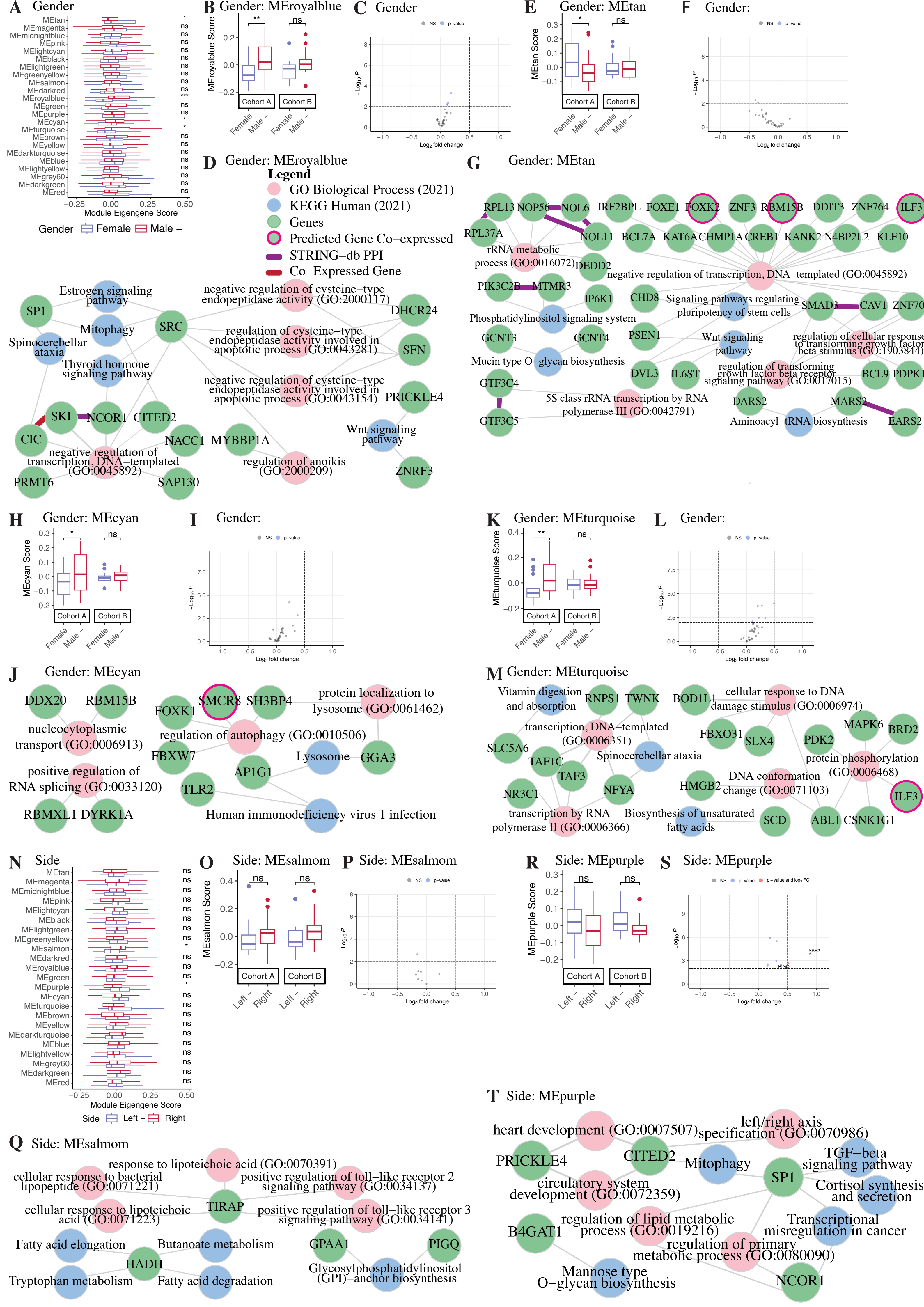

A Gender
MEtan
MEmagenta
MEmidnightblue
MEpink
MElightcyan
MEblack
MElightgreen
MEgreenyellow
MEsalmon
MEdarkred
MEroyalblue
MEgreen
MEpurple
MEcyan
MEturquoise
MEbrown
MEyellow
MEdarkturquoise
MEblue
MElightyellow
MEgrey60
MEdarkgreen
MEred
Module Eigengene Score
Gender Female Male
B Gender: MEroyalblue
MEroyalblue Score
Cohort A
Cohort B
Female
Male
C Gender
NS p−value
Log2 fold change
E Gender: MEtan
MEtan Score
F Gender:
D Gender: MEroyalblue
Legend
GO Biological Process (2021)
KEGG Human (2021)
Genes
Predicted Gene Co−expressed
STRING−db PPI
Co−Expressed Gene
Estrogen signaling pathway
SP1
Mitophagy
SRC
Spinocerebellar ataxia
Thyroid hormone signaling pathway
SKI
NCOR1
CITED2
CIC
NACC1
MYBBP1A
negative regulation of transcription, DNA−templated (GO:0045892)
PRMT6
SAP130
negative regulation of cysteine−type endopeptidase activity (GO:2000117)
DHCR24
regulation of cysteine−type endopeptidase activity involved in apoptotic process (GO:0043281)
SFN
negative regulation of cysteine−type endopeptidase activity involved in apoptotic process (GO:0043154)
PRICKLE4
Wnt signaling pathway
regulation of anoikis (GO:2000209)
ZNRF3
G Gender: MEtan
RPL13
NOP56
NOL6
IRF2BPL
FOXE1
FOXK2
ZNF3
RBM15B
DDIT3
ZNF764
ILF3
RPL37A
NOL11
BCL7A
KAT6A
CHMP1A
CREB1
KANK2
N4BP2L2
KLF10
rRNA metabolic process (GO:0016072)
DEDD2
negative regulation of transcription, DNA−templated (GO:0045892)
PIK3C2B
MTMR3
IP6K1
CHD8
Signaling pathways regulating pluripotency of stem cells
SMAD3
CAV1
ZNF70
Phosphatidylinositol signaling system
GCNT3
GCNT4
PSEN1
Wnt signaling pathway
regulation of cellular response to transforming growth factor beta stimulus (GO:1903844)
Mucin type O−glycan biosynthesis
DVL3
IL6ST
regulation of transforming growth factor beta receptor signaling pathway (GO:0017015)
BCL9
PDPK1
GTF3C4
5S class rRNA transcription by RNA polymerase III (GO:0042791)
DARS2
MARS2
GTF3C5
Aminoacyl−tRNA biosynthesis
EARS2
H Gender: MEcyan
MEcyan Score
I Gender:
K Gender: MEturquoise
MEturquoise Score
L Gender:
J Gender: MEcyan
DDX20
RBM15B
nucleocytoplasmic transport (GO:0006913)
positive regulation of RNA splicing (GO:0033120)
RBMXL1
DYRK1A
FOXK1
SMCR8
SH3BP4
protein localization to lysosome (GO:0061462)
regulation of autophagy (GO:0010506)
FBXW7
Lysosome
GGA3
AP1G1
TLR2
Human immunodeficiency virus 1 infection
M Gender: MEturquoise
Vitamin digestion and absorption
RNPS1
TWNK
BOD1L1
cellular response to DNA damage stimulus (GO:0006974)
transcription, DNA−templated (GO:0006351)
FBXO31
SLX4
MAPK6
BRD2
SLC5A6
PDK2
TAF1C
Spinocerebellar ataxia
protein phosphorylation (GO:0006468)
TAF3
DNA conformation change (GO:0071103)
HMGB2
NR3C1
NFYA
ILF3
transcription by RNA polymerase II (GO:0006366)
Biosynthesis of unsaturated fatty acids
SCD
ABL1
CSNK1G1
N Side
Side Left Right
O Side: MEsalmom
MEsalmon Score
Left
Right
P Side: MEsalmom
R Side: MEpurple
MEpurple Score
S Side: MEpurple
NS p−value p−value and log2 FC
SBF2
PIGQ
Q Side: MEsalmom
response to lipoteichoic acid (GO:0070391)
cellular response to bacterial lipopeptide (GO:0071221)
positive regulation of toll−like receptor 2 signaling pathway (GO:0034137)
TIRAP
cellular response to lipoteichoic acid (GO:0071223)
positive regulation of toll−like receptor 3 signaling pathway (GO:0034141)
Fatty acid elongation
Butanoate metabolism
GPAA1
PIGQ
HADH
Glycosylphosphatidylinositol (GPI)−anchor biosynthesis
Tryptophan metabolism
Fatty acid degradation
T Side: MEpurple
heart development (GO:0007507)
left/right axis specification (GO:0070986)
PRICKLE4
CITED2
Mitophagy
TGF−beta signaling pathway
SP1
circulatory system development (GO:0072359)
Cortisol synthesis and secretion
B4GAT1
regulation of lipid metabolic process (GO:0019216)
Transcriptional misregulation in cancer
regulation of primary metabolic process (GO:0080090)
Mannose type O−glycan biosynthesis
NCOR1

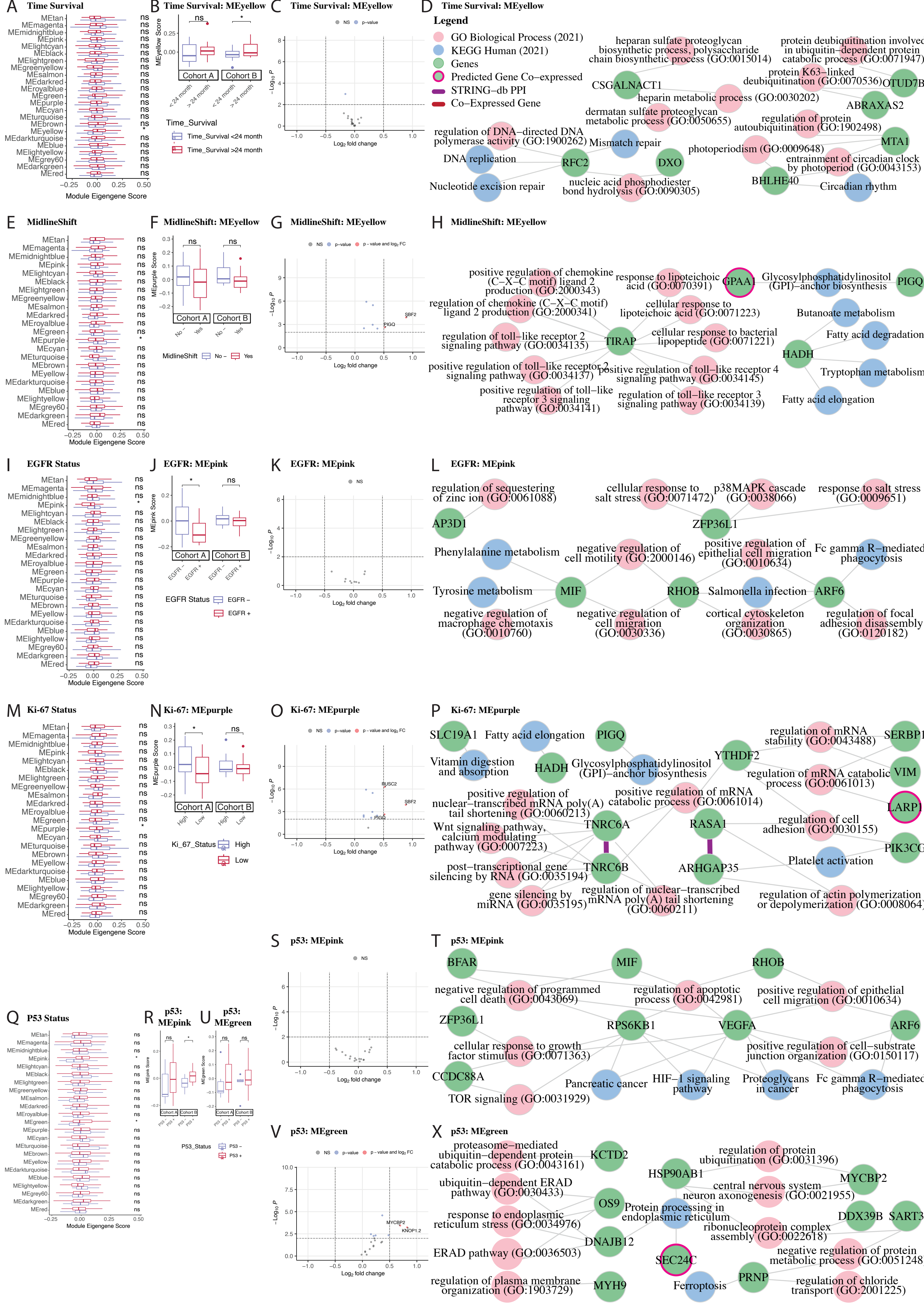
A Time Survival
B Time Survival: MEyellow
C Time Survival: MEyellow
D Time Survival: MEyellow
Module Eigengene Score
MEyellow Score
Cohort A
Cohort B
<24 month
>24 month
Time_Survival
Time_Survival <24 month
Time_Survival >24 month
Log2 fold change
NS
p−value
p − value and log2 FC
Legend
GO Biological Process (2021)
KEGG Human (2021)
Genes
Predicted Gene Co−expressed
STRING−db PPI
Co−Expressed Gene
heparan sulfate proteoglycan biosynthetic process, polysaccharide chain biosynthetic process (GO:0015014)
protein deubiquitination involved in ubiquitin−dependent protein catabolic process (GO:0071947)
CSGALNACT1
protein K63−linked deubiquitination (GO:0070536)
OTUD7B
heparin metabolic process (GO:0030202)
ABRAXAS2
dermatan sulfate proteoglycan metabolic process (GO:0050655)
regulation of protein autoubiquitination (GO:1902498)
regulation of DNA−directed DNA polymerase activity (GO:1900262)
Mismatch repair
MTA1
DNA replication
RFC2
DXO
photoperiodism (GO:0009648)
entrainment of circadian clock by photoperiod (GO:0043153)
Nucleotide excision repair
nucleic acid phosphodiester bond hydrolysis (GO:0090305)
BHLHE40
Circadian rhythm
E MidlineShift
F MidlineShift: MEyellow
G MidlineShift: MEyellow
H MidlineShift: MEyellow
MEpurple Score
MidlineShift
No −
Yes
SBF2
PIGQ
positive regulation of chemokine (C−X−C motif) ligand 2 production (GO:2000343)
response to lipoteichoic acid (GO:0070391)
GPAA1
Glycosylphosphatidylinositol (GPI)−anchor biosynthesis
PIGQ
regulation of chemokine (C−X−C motif) ligand 2 production (GO:2000341)
cellular response to lipoteichoic acid (GO:0071223)
Butanoate metabolism
regulation of toll−like receptor 2 signaling pathway (GO:0034135)
TIRAP
cellular response to bacterial lipopeptide (GO:0071221)
Fatty acid degradation
HADH
positive regulation of toll−like receptor 2 signaling pathway (GO:0034137)
positive regulation of toll−like receptor 4 signaling pathway (GO:0034145)
Tryptophan metabolism
positive regulation of toll−like receptor 3 signaling pathway (GO:0034141)
regulation of toll−like receptor 3 signaling pathway (GO:0034139)
Fatty acid elongation
I EGFR Status
J EGFR: MEpink
K EGFR: MEpink
L EGFR: MEpink
MEpink Score
EGFR −
EGFR +
EGFR Status
regulation of sequestering of zinc ion (GO:0061088)
cellular response to salt stress (GO:0071472)
p38MAPK cascade (GO:0038066)
response to salt stress (GO:0009651)
AP3D1
ZFP36L1
Phenylalanine metabolism
negative regulation of cell motility (GO:2000146)
positive regulation of epithelial cell migration (GO:0010634)
Fc gamma R−mediated phagocytosis
Tyrosine metabolism
MIF
RHOB
Salmonella infection
ARF6
negative regulation of macrophage chemotaxis (GO:0010760)
negative regulation of cell migration (GO:0030336)
cortical cytoskeleton organization (GO:0030865)
regulation of focal adhesion disassembly (GO:0120182)
M Ki-67 Status
N Ki-67: MEpurple
O Ki-67: MEpurple
P Ki-67: MEpurple
High
Low
Ki_67_Status
RUSC2
SLC19A1
Fatty acid elongation
PIGQ
regulation of mRNA stability (GO:0043488)
SERBP1
YTHDF2
Vitamin digestion and absorption
HADH
Glycosylphosphatidylinositol (GPI)−anchor biosynthesis
regulation of mRNA catabolic process (GO:0061013)
VIM
positive regulation of nuclear−transcribed mRNA poly(A) tail shortening (GO:0060213)
positive regulation of mRNA catabolic process (GO:0061014)
LARP1
Wnt signaling pathway, calcium modulating pathway (GO:0007223)
TNRC6A
RASA1
regulation of cell adhesion (GO:0030155)
PIK3CG
post−transcriptional gene silencing by RNA (GO:0035194)
TNRC6B
ARHGAP35
Platelet activation
gene silencing by miRNA (GO:0035195)
regulation of nuclear−transcribed mRNA poly(A) tail shortening (GO:0060211)
regulation of actin polymerization or depolymerization (GO:0008064)
Q P53 Status
R p53: MEpink
U p53: MEgreen
S p53: MEpink
T p53: MEpink
MEgreen Score
P53_Status
P53 −
P53 +
BFAR
MIF
RHOB
negative regulation of programmed cell death (GO:0043069)
regulation of apoptotic process (GO:0042981)
positive regulation of epithelial cell migration (GO:0010634)
ZFP36L1
RPS6KB1
VEGFA
ARF6
cellular response to growth factor stimulus (GO:0071363)
positive regulation of cell−substrate junction organization (GO:0150117)
CCDC88A
Pancreatic cancer
HIF−1 signaling pathway
Proteoglycans in cancer
Fc gamma R−mediated phagocytosis
TOR signaling (GO:0031929)
V p53: MEgreen
X p53: MEgreen
MYCBP2
KNOP1.2
proteasome−mediated ubiquitin−dependent protein catabolic process (GO:0043161)
KCTD2
regulation of protein ubiquitination (GO:0031396)
HSP90AB1
MYCBP2
ubiquitin−dependent ERAD pathway (GO:0030433)
central nervous system neuron axonogenesis (GO:0021955)
OS9
Protein processing in endoplasmic reticulum
response to endoplasmic reticulum stress (GO:0034976)
ribonucleoprotein complex assembly (GO:0022618)
DDX39B
SART3
DNAJB12
ERAD pathway (GO:0036503)
SEC24C
negative regulation of protein metabolic process (GO:0051248)
regulation of plasma membrane organization (GO:1903729)
MYH9
Ferroptosis
PRNP
regulation of chloride transport (GO:2001225)

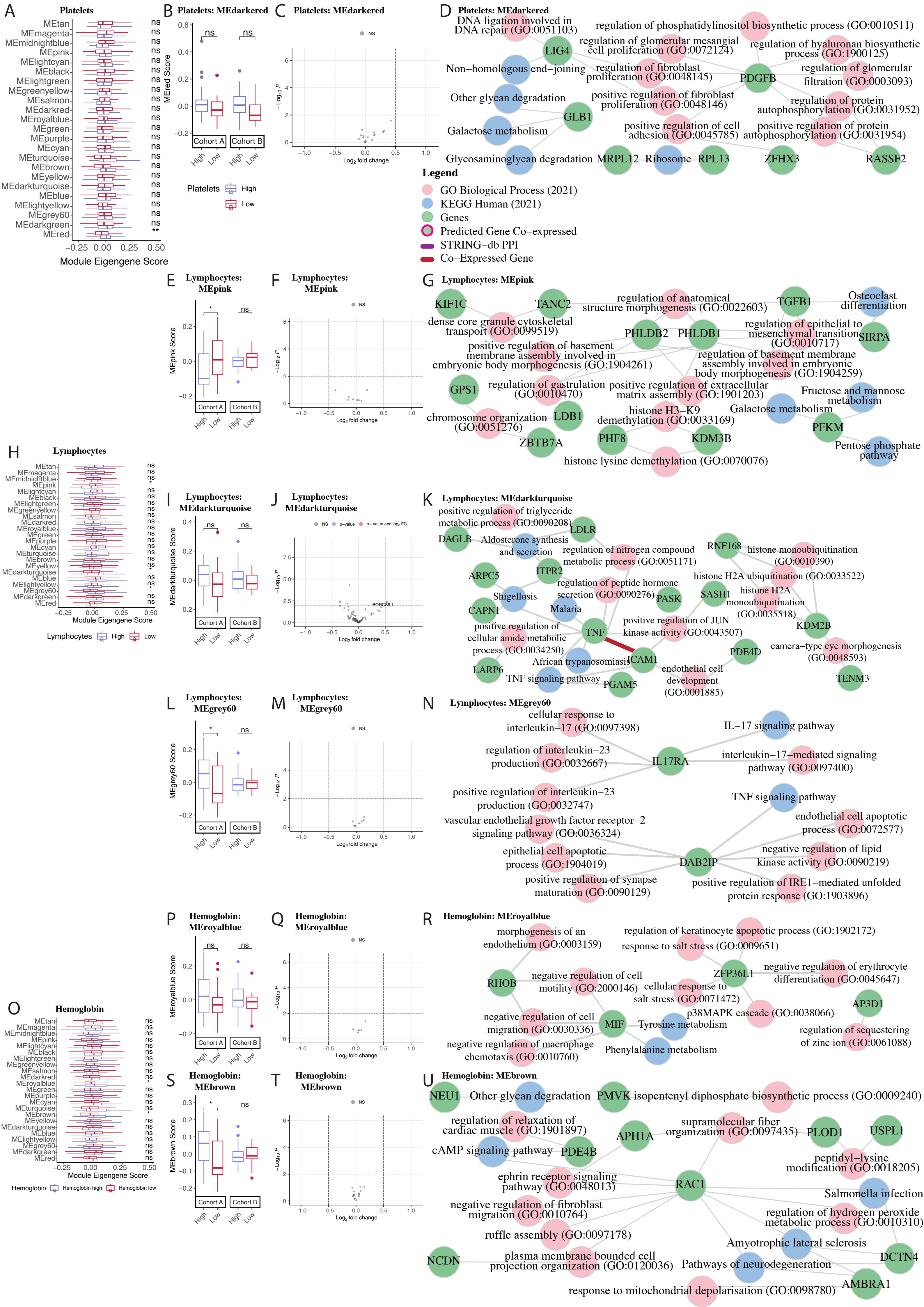
A
Platelets
MEtan
MEmagenta
MEmidnightblue
MEpink
MElightcyan
MEblack
MElightgreen
MEgreenyellow
MEsalmon
MEdarkred
MEroyalblue
MEgreen
MEpurple
MEcyan
MEturquoise
MEbrown
MEyellow
MEdarkturquoise
MEblue
MElightyellow
MEgrey60
MEdarkgreen
MEred
ns
**
−0.25 0.00 0.25 0.50
Module Eigengene Score
B Platelets: MEdarkered
MEred Score
Cohort A
Cohort B
High
Low
Platelets
High
Low
C Platelets: MEdarkered
NS
Log2 fold change
D Platelets: MEdarkered
DNA ligation involved in DNA repair (GO:0051103)
regulation of phosphatidylinositol biosynthetic process (GO:0010511)
LIG4
regulation of glomerular mesangial cell proliferation (GO:0072124)
regulation of hyaluronan biosynthetic process (GO:1900125)
Non−homologous end−joining
regulation of fibroblast proliferation (GO:0048145)
PDGFB
regulation of glomerular filtration (GO:0003093)
Other glycan degradation
positive regulation of fibroblast proliferation (GO:0048146)
regulation of protein autophosphorylation (GO:0031952)
GLB1
Galactose metabolism
positive regulation of cell adhesion (GO:0045785)
positive regulation of protein autophosphorylation (GO:0031954)
Glycosaminoglycan degradation
MRPL12
Ribosome
RPL13
ZFHX3
RASSF2
Legend
GO Biological Process (2021)
KEGG Human (2021)
Genes
Predicted Gene Co−expressed
STRING−db PPI
Co−Expressed Gene
E Lymphocytes: MEpink
MEpink Score
F Lymphocytes: MEpink
G Lymphocytes: MEpink
KIF1C
TANC2
regulation of anatomical structure morphogenesis (GO:0022603)
TGFB1
Osteoclast differentiation
dense core granule cytoskeletal transport (GO:0099519)
PHLDB2
PHLDB1
regulation of epithelial to mesenchymal transition (GO:0010717)
SIRPA
positive regulation of basement membrane assembly involved in embryonic body morphogenesis (GO:1904261)
regulation of basement membrane assembly involved in embryonic body morphogenesis (GO:1904259)
GPS1
regulation of gastrulation (GO:0010470)
positive regulation of extracellular matrix assembly (GO:1901203)
Fructose and mannose metabolism
chromosome organization (GO:0051276)
LDB1
histone H3−K9 demethylation (GO:0033169)
Galactose metabolism
PFKM
ZBTB7A
PHF8
KDM3B
Pentose phosphate pathway
histone lysine demethylation (GO:0070076)
H
Lymphocytes
Lymphocytes
High
Low
I Lymphocytes: MEdarkturquoise
MEdarkturquoise Score
J Lymphocytes: MEdarkturquoise
NS
p−value
p − value and log2 FC
BOPCS6.1
K Lymphocytes: MEdarkturquoise
positive regulation of triglyceride metabolic process (GO:0090208)
DAGLB
Aldosterone synthesis and secretion
LDLR
regulation of nitrogen compound metabolic process (GO:0051171)
RNF168
histone monoubiquitination (GO:0010390)
ITPR2
ARPC5
regulation of peptide hormone secretion (GO:0090276)
histone H2A ubiquitination (GO:0033522)
Shigellosis
PASK
SASH1
histone H2A monoubiquitination (GO:0035518)
CAPN1
Malaria
positive regulation of cellular amide metabolic process (GO:0034250)
TNF
positive regulation of JUN kinase activity (GO:0043507)
KDM2B
ICAM1
PDE4D
camera−type eye morphogenesis (GO:0048593)
African trypanosomiasis
LARP6
TNF signaling pathway
PGAM5
endothelial cell development (GO:0001885)
TENM3
L Lymphocytes: MEgrey60
MEgrey60 Score
M Lymphocytes: MEgrey60
N Lymphocytes: MEgrey60
cellular response to interleukin−17 (GO:0097398)
IL−17 signaling pathway
regulation of interleukin−23 production (GO:0032667)
IL17RA
interleukin−17−mediated signaling pathway (GO:0097400)
positive regulation of interleukin−23 production (GO:0032747)
TNF signaling pathway
vascular endothelial growth factor receptor−2 signaling pathway (GO:0036324)
endothelial cell apoptotic process (GO:0072577)
epithelial cell apoptotic process (GO:1904019)
DAB2IP
negative regulation of lipid kinase activity (GO:0090219)
positive regulation of synapse maturation (GO:0090129)
positive regulation of IRE1−mediated unfolded protein response (GO:1903896)
O
Hemoglobin
Hemoglobin
Hemoglobin high
Hemoglobin low
P Hemoglobin: MEroyalblue
MEroyalblue Score
Q Hemoglobin: MEroyalblue
R Hemoglobin: MEroyalblue
morphogenesis of an endothelium (GO:0003159)
regulation of keratinocyte apoptotic process (GO:1902172)
response to salt stress (GO:0009651)
ZFP36L1
negative regulation of erythrocyte differentiation (GO:0045647)
RHOB
negative regulation of cell motility (GO:2000146)
cellular response to salt stress (GO:0071472)
AP3D1
negative regulation of cell migration (GO:0030336)
MIF
Tyrosine metabolism
p38MAPK cascade (GO:0038066)
regulation of sequestering of zinc ion (GO:0061088)
negative regulation of macrophage chemotaxis (GO:0010760)
Phenylalanine metabolism
S Hemoglobin: MEbrown
MEbrown Score
T Hemoglobin: MEbrown
U Hemoglobin: MEbrown
NEU1
Other glycan degradation
PMVK
isopentenyl diphosphate biosynthetic process (GO:0009240)
regulation of relaxation of cardiac muscle (GO:1901897)
APH1A
supramolecular fiber organization (GO:0097435)
PLOD1
USPL1
cAMP signaling pathway
PDE4B
peptidyl−lysine modification (GO:0018205)
ephrin receptor signaling pathway (GO:0048013)
RAC1
Salmonella infection
negative regulation of fibroblast migration (GO:0010764)
regulation of hydrogen peroxide metabolic process (GO:0010310)
ruffle assembly (GO:0097178)
Amyotrophic lateral sclerosis
NCDN
plasma membrane bounded cell projection organization (GO:0120036)
Pathways of neurodegeneration
DCTN4
response to mitochondrial depolarisation (GO:0098780)
AMBRA1

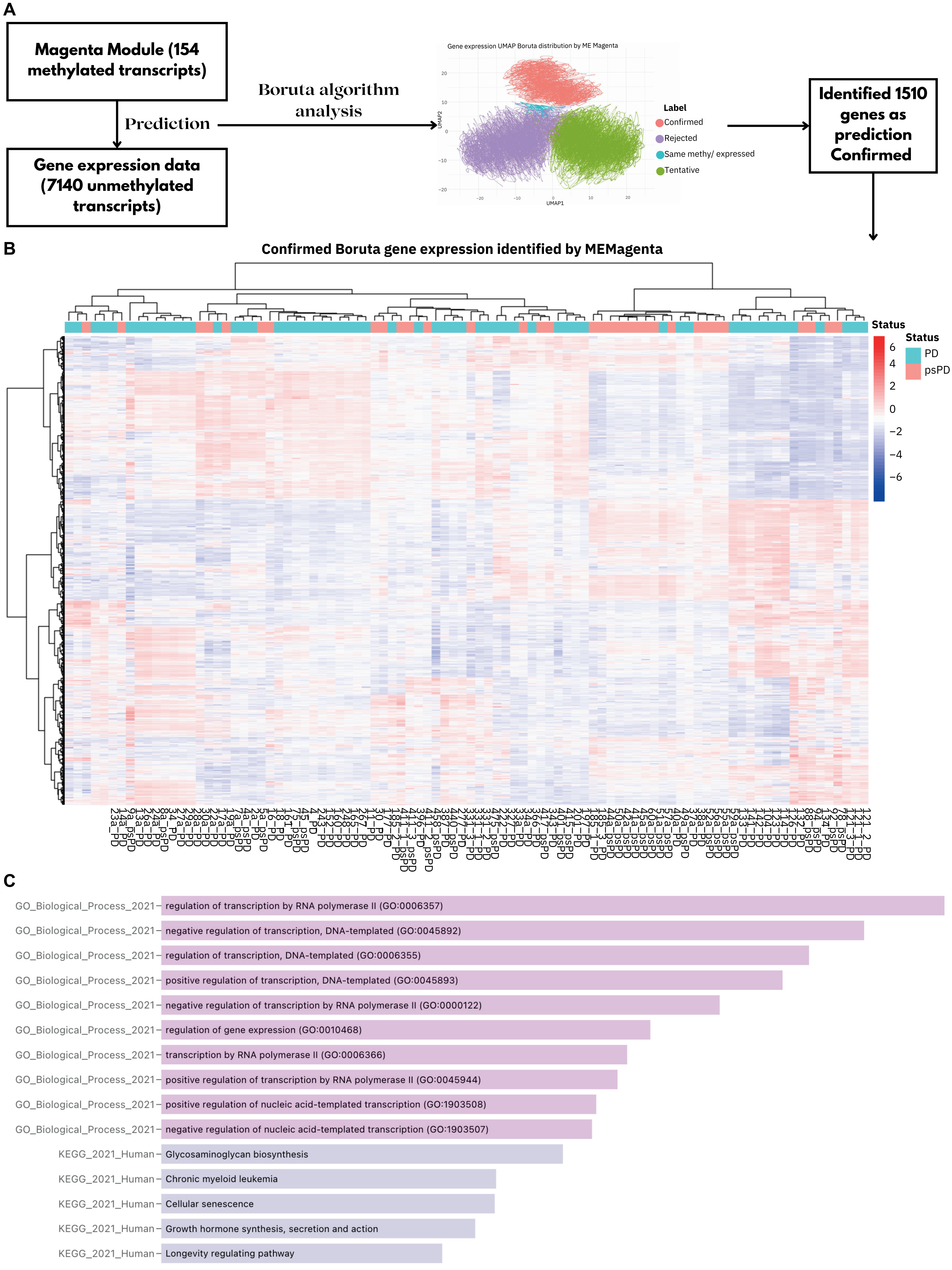
A
Magenta Module (154 methylated transcripts)
Prediction
Gene expression data (7140 unmethylated transcripts)
Boruta algorithm analysis
Gene expression UMAP Boruta distribution by ME Magenta
UMAP2
UMAP1
Label
Confirmed
Rejected
Same methy/ expressed
Tentative
Identified 1510 genes as prediction Confirmed
B
Confirmed Boruta gene expression identified by MEMagenta
Status
PD
psPD
C
GO_Biological_Process_2021 – regulation of transcription by RNA polymerase II (GO:0006357)
GO_Biological_Process_2021 – negative regulation of transcription, DNA-templated (GO:0045892)
GO_Biological_Process_2021 – regulation of transcription, DNA-templated (GO:0006355)
GO_Biological_Process_2021 – positive regulation of transcription, DNA-templated (GO:0045893)
GO_Biological_Process_2021 – negative regulation of transcription by RNA polymerase II (GO:0000122)
GO_Biological_Process_2021 – regulation of gene expression (GO:0010468)
GO_Biological_Process_2021 – transcription by RNA polymerase II (GO:0006366)
GO_Biological_Process_2021 – positive regulation of transcription by RNA polymerase II (GO:0045944)
GO_Biological_Process_2021 – positive regulation of nucleic acid-templated transcription (GO:1903508)
GO_Biological_Process_2021 – negative regulation of nucleic acid-templated transcription (GO:1903507)
KEGG_2021_Human – Glycosaminoglycan biosynthesis
KEGG_2021_Human – Chronic myeloid leukemia
KEGG_2021_Human – Cellular senescence
KEGG_2021_Human – Growth hormone synthesis, secretion and action
KEGG_2021_Human – Longevity regulating pathway
