# Supplementary material for: Epitranscriptomic analysis reveals clinical and molecular signatures in glioblastoma
Source: Acta Neuropathol Commun. 2025 Apr 11;13:74. doi: 10.1186/s40478-025-01966-5 (PMC11987271; doi:10.1186/s40478-025-01966-5)
Supplement: Supplementary file 7 — Supplementary Material 7 [file 40478_2025_1966_MOESM7_ESM.docx]

**Additional file 2: Figure S1: portrays essential analyses in the field of RNA methylation, specifically focusing on the % m6A and its influence on gene expression.** (A) workflow to choose the best approach by comparing the batch effect removal methods. (B-C) Heatmap delineate the distribution of m6A percentages in both the Cohort A and Cohort B groups, respectively. (D-H) RLE plots, while (J-O) showcase PCA plots, illustrating the implementation of batch normalization strategies. (D and J) display the data in its unnormalized state; (E and M) exhibit QN; (F and K) exemplify the utilization of the PamR package for normalization; (G and N) depict the combined implementation of QN and the PamR package; (H and L) demonstrate the application of Combat adjustment; (I and O) present the utilization of combined QN and Combat adjustment. (P) Heatmap post batch effect removal using the combined QN and Combat methodology.

**Additional file 2: Figure S2: portrays essential analyses in the field of gene expression.** (A) workflow to choose the best approach by comparing the batch effect removal methods. (B-C) Heatmap delineate the distribution of expression abundance in both the Cohort A and Cohort B groups, respectively. (D-H) RLE plots, while (J-O) showcase PCA plots, illustrating the implementation of batch normalization strategies. (D and J) display the data in its unnormalized state; (E and M) exhibit QN; (F and K) exemplify the utilization of the PamR package for normalization; (G and N) depict the combined implementation of QN and the PamR package; (H and L) demonstrate the application of Combat adjustment; (I and O) present the utilization of combined QN and Combat adjustment. (P) Heatmap post batch effect removal using the combined QN and Combat methodology.

**Additional file 3: Figure S3:** **Validating the Purity of Hierarchical Cluster Labeling of Clinical and Histopathological Variables.** (A) heatmap showcasing the comprehensive array of clinical and histopathological variables among the subjects under investigation. (B-W) Mosaic plot compare cluster formation across each clinical and histopathological variable within the studied patient cohort by T test. P < 0.05 was consider statistically significant.

**Additional file 4: Figure S4: Analysis of Methylated Transcript Variability and Biological Associations.** (A) Barplot illustrating variability among 5690 methylated transcripts, while (B) represents variability in the top 30% (1720 transcripts) with highest variance, showcasing the top 10 significantly enriched Gene Ontology (GO) and KEGG pathways ordered by p-values (p < 0.05). (C) Depicts variability across 49 representative genes of all methylated transcripts, and (D) displays 52 representative genes of the top 30% with higher variance, forming a subnetwork highlighting key genes and major GO and KEGG pathways associated with methylated transcripts.

**Additional file 5: Figure S5: Comparative Analysis of Clinical Factors and Module-Specific Gene Expression in Glioblastoma.** (A-E) Comparative analyses between clinical and histopathological factors such as Age, Life Status, Other Tumors, BMI, and MGMT. (F, I, L, and O) Barplot from Diagnosis (PD vs. psPD) comparisons across Cohort A and Cohort B batches within midnightblue, green, turquoise, and tan modules. (G, J, M, and P) Volcano plots delineate differentially expressed genes within methylated regions of each module. (H, F, N, and Q) present enrichment-augmented subnetworks showing gene-gene interactions and associated sub-products linked to primary GO processes and KEGG pathways. All depicted associations in subnetworks maintain significant p-values (p < 0.05). Initial comparisons were ANOVA screened, with specific groupings tested via t test. Statistically significant effects are annotated as *p < 0.05; **p < 0.01; ***p < 0.001; ****p < 0.0001.

**Additional file 5: Figure S6: Comparative Analysis of Clinical Factors and Module-Specific Gene Expression in Glioblastoma.** Comparative analyses between clinical such as (A) Gender and (N) Side Location. Barplot from Gender (Male and Female) comparisons across Cohort A and Cohort B batches within (B) MEsalmon, (E) MEmagenta, (H) Mlightgreen, and (K) MEblack modules and Side Location (Left and Right) within (O) MEsalmon and (R) MEpurple modules. Volcano plots delineate DEG within methylated regions of each (C) MEsalmon, (F) MEmagenta, (I) MElightgreen, and (L) MEblack module in Gender, and (P) MEsalmon and (S) MEpurple in Side Location. Enrichment-augmented subnetworks showing gene-gene interactions and associated sub-products linked to mainly GO processes and KEGG pathways of each (D) MEsalmon, (G) MEmagenta, (J) MElightgreen, and (K) MEblack modules in Gender, and (Q) MEsalmon and (T) MEpurple modules in Side Location. All depicted associations in subnetworks maintain significant p-values (p < 0.05). Initial comparisons were ANOVA screened, with specific groupings tested via t test. Statistically significant effects are annotated as *p < 0.05; **p < 0.01; ***p < 0.001; ****p < 0.0001.

**Additional file 5: Figure S7: Comparative Analysis of Clinical Factors and Module-Specific Gene Expression in Glioblastoma.** Comparative analyses between clinical such as (A) Time Survival, (E) Midshift Line, (I) EGFR, (M) Ki-67 and (Q) p53 Status. Barplot from comparisons across Cohort A and Cohort B batches within (B) Time Survival and MEyellow, (F) Midshift Line and MEpurple, (J) EGFR and MEpink, (N) Ki-67 and MEpurple and p53 Status and (R) MEpink and (U) MEgreen modules, respectively. Volcano plots delineate DEG within methylated regions of (C) Time Survival and MEyellow, (G) Midshift Line and MEpurple, (K) EGFR and MEpink, (O) Ki-67 and MEpurple and p53 Status and (S) MEpink and (V) MEgreen modules. Enrichment-augmented subnetworks showing gene-gene interactions and associated sub-products linked to mainly GO processes and KEGG pathways of (D) Time Survival and MEyellow, (H) Midshift Line and MEpurple, (L) EGFR and MEpink, (P) Ki-67 and MEpurple and p53 Status and (T) MEpink and (X) MEgreen modules. All depicted associations in subnetworks maintain significant p-values (p < 0.05). Initial comparisons were ANOVA screened, with specific groupings tested via t test. Statistically significant effects are annotated as *p < 0.05; **p < 0.01; ***p < 0.001; ****p < 0.0001.

**Additional file 5: Figure S8: Comparative Analysis of Clinical Factors and Module-Specific Gene Expression in Glioblastoma.** Comparative analyses between clinical features such as (A) Platelets, (H) Lymphocytes and (O) Hemoglobin. Barplot from comparisons across Cohort A and Cohort B batches within (B) Platelets and MEdarkred; Lymphocytes and (E) MEgreen, (I) MEyoellow, and (L) MEpruple; Hemoglobin and (P) MEsalmon, and (S) MEbrown modules. Volcano plots delineate DEG within methylated regions of (C) Platelets and MEdarkred; Lymphocytes and (F) MEgreen, (J) MEyoellow, and (M) MEpruple; Hemoglobin and (Q) MEsalmon, and (T) MEbrown modules. Enrichment-augmented subnetworks showing gene-gene interactions and associated sub-products linked to mainly GO processes and KEGG pathways of (D) Platelets and MEdarkred; Lymphocytes and (G) MEgreen, (K) MEyoellow, and (N) MEpruple; Hemoglobin and (R) MEsalmon, and (U) MEbrown modules. All depicted associations in subnetworks maintain significant p-values (p < 0.05). Initial comparisons were ANOVA screened, with specific groupings tested via t test. Statistically significant effects are annotated as *p < 0.05; **p < 0.01; ***p < 0.001; ****p < 0.0001.

**Additional file 5: Figure S9: Comparative Analysis of White Blood Cells and Module-Specific Gene Expression in Glioblastoma.** Comparative analyses between clinical features such as (A) WBC. Barplot from comparisons across Cohort A and Cohort B batches within WBC and (B) MEsalmon, (R) MEmagenta, (H) MEgreeb, and (K) MEpruple. Volcano plots delineate DEG within methylated regions of WBC and (C) MEsalmon, (F) MEmagenta, (I) MEgreen, and (L) MEpruple. Enrichment-augmented subnetworks showing gene-gene interactions and associated sub-products linked to mainly GO processes and KEGG pathways of WBC and (D) MEsalmon, (G) MEmagenta, (J) MEgreeb, and (M) MEpruple. All depicted associations in subnetworks maintain significant p-values (p < 0.05). Initial comparisons were ANOVA screened, with specific groupings tested via t test. Statistically significant effects are annotated as *p < 0.05; **p < 0.01; ***p < 0.001; ****p < 0.0001.

**Additional file 5: Figure S10: Comparative Analysis of Lobe Location and Module-Specific Gene Expression in Glioblastoma.** Comparative analyses between clinical features such as (A) Lobe location. Barplot from comparisons across Cohort A and Cohort B batches within Lobe location and (B) MEblue, (E) MEdarckgreen, (H) MEgreen, (K) greenyellow and (N) MElightcyan modules. Volcano plots delineate DEG within methylated regions of Lobe location and (C) MEblue, (F) MEdarckgreen, (I) MEgreen, (L) greenyellow and (O) MElightcyan modules. Enrichment-augmented subnetworks showing gene-gene interactions and associated sub-products linked to mainly GO processes and KEGG pathways of Lobe location and (D) MEblue, (G) MEdarckgreen, (J) MEgreen, (M) greenyellow and (P) MElightcyan modules. All depicted associations in subnetworks maintain significant p-values (p < 0.05). Initial comparisons were ANOVA screened, with specific groupings tested via t test. Statistically significant effects are annotated as *p < 0.05; **p < 0.01; ***p < 0.001; ****p < 0.0001.

**Additional file 5: Figure S11: WGCNA module validation.** (A) Machine learning workflow to validate the magenta module with the gene expression. (B) Heatmap showing the gene expression and (C) GO processes and KEGG pathways of the confirmed genes identified by the Boruta analysis related with the magenta module.

**Additional file 6: Figure S12: Validating the Purity of Hierarchical Cluster Labeling.** (A) Workflow outlines the sequential steps involved in the comparison of label purity in clinical and histopathological groups. (B) Heatmap showcasing the comprehensive array of Diagnosis variable among the PD/psPD patients with glioblastoma. (C) Mosaic plot compares cluster formation across the Diagnosis variable within the studied PD/psPD patient cohort by Fisher's Exact Test. (A) Workflow of the performance and validation of the HC. (B) Distribution plot of the three clusters, showing the two most relevant principal component. (C) Barplot showing RMSE, ARMSE, MAPE and CVIM metrics comparing Cohort A as a training and Cohort B B as test and the inverse. (D) Boxplot showing silhouette and ARI metric. (E) Workflow for m6A% and DMT clustering formation relation and (F) Alluvial plot comparison with ARI results. (G) Workflow for DMT and DMT with DEG overlapping clustering formation relation and (H) Alluvial plot comparison with ARI results.
